# TGF-β1 Induces Mucosal Mast Cell Genes and is Negatively Regulated by the IL-3/ERK1/2 Axis

**DOI:** 10.1186/s12964-025-02048-8

**Published:** 2025-02-11

**Authors:** Steffen K. Meurer, Gina Bronneberg, Christian Penners, Marlies Kauffmann, Till Braunschweig, Christian Liedtke, Michael Huber, Ralf Weiskirchen

**Affiliations:** 1 Institute of Molecular Pathobiochemistry, Experimental Gene Therapy and Clinical Chemistry (IFMPEGKC), Medical Faculty, RWTH Aachen University, 52074 Aachen, Germany; 2 Institute of Biochemistry and Molecular Immunology, Medical Faculty, RWTH Aachen University, Aachen, Germany; 3 Department of Internal Medicine III, Medical Faculty, RWTH Aachen University, Aachen, Germany; 4 Institute of Pathology, Ludwig-Maximilians-University, Munich, Germany

**Keywords:** TGF-β-receptor, TGF-β-signaling, SMADs, Mast cell differentiation, MCPT1, IL-3, ERK1/2

## Abstract

Mast cells develop from the myeloid lineage and are released from the bone marrow as immature cells, which then differentiate at the destination tissue based on cues from the local environment. In the liver, mast cells are recruited in diseased states to fibrogenic surroundings rich in TGF-β1. The aim of this study was to investigate TGF-β1 signaling in primary and permanent mast cells to identify common and unique mechanisms. The TGF-β receptor repertoire is similar among mast cells, with high expression of type I and type II receptors and very low expression of type III receptors (Betaglycan and Endoglin). Downstream, TGF-β1 activates the SMAD2/3 signaling axis and also SMAD1/5 with target genes *Smad6* and *Id1* in a transient manner. Initially, TGF-β1 upregulates the transcription of mucosal mast cell effectors *Mcpt1* and *Mcpt2* in all analyzed mast cells. This upregulation is reduced in the presence of IL-3, which promotes proliferation. Inhibition of ERK1/2 activation reduces proliferation and mitigates the negative effect of IL-3 on *Mcpt1* mRNA and protein expression in the immortalized mast cell line PMC-306 but not in bone marrow-derived mast cells. Therefore, extracellular signal-regulated kinases ERK1/2 are identified as a mutual switch between IL-3-driven proliferation and TGF-β1-promoted mucosal mast cell differentiation in PMC-306. In conclusion, TGF-β1 promotes a mucosal gene signature and inhibits proliferation in mast cells, with these effects being counter-regulated by IL-3/ERK1/2.

**Graphical Abstract:**

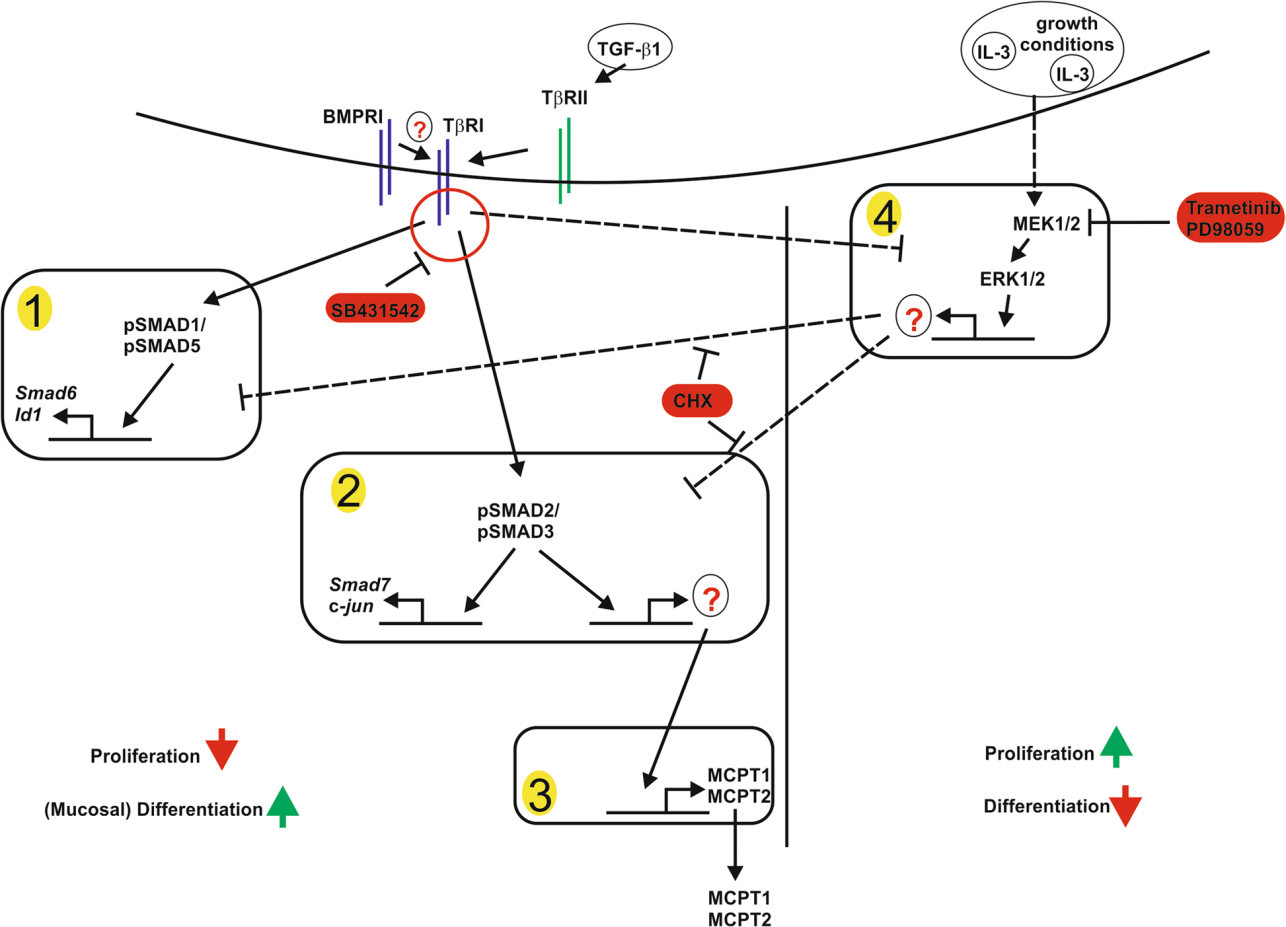

**Supplementary Information:**

The online version contains supplementary material available at 10.1186/s12964-025-02048-8.

## Summary of findings

TGF-β1 binds to the core receptors type II (TβRII) and type I (TβRI), which are highly expressed in mast cells (MC). Although MC do not express ALK1, SMAD2/3 and Smad1/5 are activated (1, 2). All responses elicited by TGF-β1 analyzed herein are dependent on ALK5 as they are blocked by the application of SB431542, an inhibitor of ALK5. Activation of SMAD1/5 can be monitored by target gene expression of *Smad6/Id1,* which are up-regulated by cycloheximide (CHX) and reduced by IL-3 (1). SMAD1/5 activation is transient, starting at 30 min and turning off at 24 h, as is the expression of target genes *Smad6* and *Id1* (1). The SMAD2/3 activation is persistent, as indicated by phosphorylation of SMAD2 and *c-jun/Smad7* gene expression, both immediate early and late (2). Similar to SMAD1/5 signaling, SMAD2 responses are enhanced by CHX (2). TGF-β1/SMAD2 signaling leads to the induction of several MC effectors, mainly of mucosal type markers including *Mcpt1*and *Mcpt2* (3). In PMC-306 cells, first-line induction is detectable after 90 min. The addition of CHX during TGF-β1 stimulation reduces the *Mcpt1* up-regulation. This is most likely due to the fact that TGF-β1 engages a secondary response (X) to induce *Mcpt1* and *Mcpt2* after 6 h (3). As a putative mediator of this inhibitory effect on TGF-β1 signaling (uncovered by CHX) in PMC-306 cells, we identified IL-3-mediated ERK1/2 activation (1,2,4). Blocking of ERK1/2 activation by Trametinib increases TGF-β1 regulated *Mcpt1* expression in the presence of IL-3 (4).

## Introduction

Mast cells (MCs) are immune cells that play a central role in type 2 inflammatory reactions [1]. MCs originate from the myelogenic hematopoietic lineage [2]. In adults, immature MCs develop in the bone marrow and, upon release they migrate to and enter their target tissues. Importantly, MCs mature and differentiate at their destination site in the periphery [3]. Additionally, long-lived connective tissue-like MCs are present in organ systems during organ development [4]. In pathological conditions, short-lived MCs with a mucosal phenotype can be recruited to affected organs [5]. The cues for the final differentiation of MCs come from the local environment, where the precursors are directed [6]. Since MCs are found in various tissues throughout the body [7], their differentiation occurs in different individual microenvironments, resulting in highly versatile, phenotypically variable cells shaped by their tissue of residence [8, 9]. Therefore, MCs exhibit phenotypic and functional diversity [6, 10]. The quantities of MCs in different organ systems under normal conditions vary significantly, with high levels in the skin, tongue and stomach, but low levels in the spleen, thymus and liver [7].

Although MCs are a heterogeneous cell population, in mice and rats, they can be categorized into two main groups: T-cell independent connective tissue MCs (CTMCs) mainly found in the skin and peritoneal cavity, and T-cell dependent mucosal MCs primarily found in the gut mucosa and lungs [10]. The distinction between these groups is based on their granule content, which includes different protease repertoires, bioactive compounds such as histamine and heparin, and the presence of different receptors. CTMCs typically contain tryptase, chymases, carboxypeptidase A3 (CPA3), histamine, and heparin proteoglycans [11, 12]. These CTMCs are predominantly present in the skin and peritoneum and can be isolated and cultured from the peritoneal cavity as peritoneum-derived MCs (PMCs) [13]. On the other hand, mucosal MCs (MMCs) lack tryptase and instead contain primarily chondroitin sulphate, along with other granule components [11]. In mice, “mucosal-like” MCs can be differentiated in culture from multipotent bone marrow-derived precursor cells in the presence of IL-3 and, optionally stem cell factor (SCF) [14]. However, differentiated cultures of murine bone marrow-derived MCs (BMMCs) often result in an incompletely differentiated population of MCs that do not fully resemble end-differentiated MCs observed in mouse tissue in terms of morphology and histochemistry [15]. The mixed phenotype of BMMCs was highlighted in a recent publication that compared the transcriptome of BMMCs with that of primary PMCs [16].

The primary function of MCs is to maintain organ homeostasis in response to changing environmental conditions. They are located at the internal and external interfaces of the body in mucosal tissues, serving as the first line of defense against intruders [17]. MCs are equipped with effector molecules stored in their granules, which are released upon activation by harmful agents. In addition to their immune-related defense functions, it has been recognized that MCs play a role in fibrogenic processes in various organs [18]. Recently, the role of MCs in liver fibrosis has gained attention [19]. While MC counts are very low in healthy mouse livers [7, 20, 21], MCs have been found to accumulate in livers affected by cholangiopathies [22, 23]. One factor that may contribute to the recruitment of MCs to the liver is stem cell factor (SCF), which is released by activated hepatic stellate cells (HSC) [24]. In rats, but not in mice, infection with the bacterial strain *Schistosoma mansoni* has been shown to cause an accumulation of mucosal MCs [25]. Conversely, bile duct ligation in rats leads to the accumulation of toluidine blue-positive MCs in the liver, which can be isolated and characterized [26]. These findings suggest that there may be different subtypes of MCs recruited to and detected in the liver. Cholangiopathies have primarily been studied in humans, as well as in rat and mouse models. In cases of cholestasis, MCs infiltrate the liver and can be found in the periportal fields near proliferating bile ducts (ductular reaction) and deposits of extracellular matrix, as well as in sinusoids using toluidine blue or tryptase/chymase immunodetection [21, 27]. However, it has been noted that the immunodetection of tryptase is more sensitive than the conventional histochemical stain with toluidine blue [21]. Nonetheless, MCs can be easily identified using toluidine blue stain in human livers. However, this histochemical approach is not applicable for detecting MCs in mouse livers, although the increase of tryptase in the affected mouse liver at the mRNA and protein level suggests the presence of connective tissue MCs (CTMCs) [27, 28].

As mentioned earlier, it is essential to understand the behaviour of immature infiltrating MCs in the presence of TGF-β1, which is known to be abundant in liver fibrosis. This understanding encompasses their differentiation and biological functions. TGF-β1 plays a role in regulating various aspects of MC biology, including proliferation/apoptosis, migration, degranulation and marker expression [29–37]. Regarding differentiation/maturation, most studies suggest that TGF-β1 has a positive influence on MCs, leading to increased expression of markers specific to CTMCs like MCPT6/MCPT7, and mucosal MCs like MCPT1/MCPT2/ITGAE. However, there are conflicting findings regarding the impact of TGF-β1 on MC maturation [38].

Finally, mature MCs themselves have been shown to produce large amounts of TGF-β1 [39]. This is further supported by the finding that TGF-β1, released by mature MCs injected into wild-type (WT) or MC-depleted mice, induces liver fibrosis. This effect can be blocked by pre-treating transferred MCs with a TGF-β receptor inhibitor [40].

TGF-β1 signaling is initiated by enzymatic (e.g., chymase) or integrin-mediated (e.g., αVβ6) liberation of the ligand from the large latent TGF-β complex deposited in the extracellular matrix [41, 42]. Activation of the ligand allows it to bind to a typeII receptor (TβRII) dimer of the core receptor complex. This leads to the recruitment of a type I receptor (ALK5) dimer and subsequent phosphorylation of the transcription factors SMAD2/SMAD3 by ALK5. The activated SMADs bind to the common SMAD4, translocate to the nucleus and regulate the transcription of target genes in association with other positive and negative transcription factors. One of the target genes is *Smad7* which functions as a negative feedback regulator, interfering with TGF-β-signaling at multiple steps [43]. In several cells it has been shown that TGF-β1 activates, in addition to SMAD2/SMAD3, the “BMP”-SMADs 1/5/8 via the type I receptor ALK1. The balance between TGF-β-mediated activation of ALK5 and ALK1 is facilitated by the type III receptor Endoglin [44].

Since MCs express TGF-β receptors, TGF-β1 directly affects and regulates MC responses [35, 45]. These responses include proliferation, apoptosis, and a direct transcriptional induction of *Mcpt1* and *Mcpt6/7* by the transcription factors SMAD2/SMAD3 [33, 46, 47]. SMAD2 has been shown to regulate MCPT1/MCPT2 expression and SMAD3 regulates *Mcpt6*/*Mcpt7* transcription, suggesting a specification of SMAD2 and SMAD3 in MC differentiation [46, 47]. Released chymase and tryptase can activate matrix-stored TGF-β1, and hepatic stellate cells via the tryptase-sensitive PAR-2 receptor, mutually fuelling liver fibrosis [48].

In this study, we analyse TGF-β signalling components and modes of signalling by comparing established MCs like primary BMMCs and immortalized L138.8A MCs with the recently generated PMC cell line PMC-306. We demonstrate that MCs generally express high amounts of the core receptors but almost no type III accessory receptors, Betaglycan and Endoglin. We show for the first time a transient activation of SMAD1/SMAD5 and expression of the target genes *Smad6* and *Id1,* in addition to the SMAD2/SMAD3 signalling branch. TGF-β prominently induces mucosal MC effectors *Mcpt1* and *Mcpt2*, and these effects are diminished in growth conditions that promote proliferation. Blocking IL-3-mediated ERK1/2 activation decreases proliferation but enhances TGF-β-mediated *Mcpt1* mRNA and MCPT1 protein expression. Therefore, TGF-β1/ALK5/SMAD2 and IL-3/ERK1/2 pathways directly converge to balance differentiation and proliferation, however the exact molecular mechanisms are yet to be elucidated.

## Materials and Methods

### Reagents

Sources of special reagents (cytokines, inhibitors, protease inhibitors, dyes, transfection reagents, and others) used in this study are listed in Suppl. Table 1.

### Animals and Isolation of Primary Mast Cells

The isolation of primary cells and organs from donor mice was conducted in accordance with German legal requirements and animal protection laws. This process was approved by the authority for environment conservation and consumer protection of the state North Rhine-Westphalia (LANUV). Animals were housed in a temperature-controlled room with 12-h light/dark cycles and had free access to food and water. Murine PMCs and BMMCs were isolated and cultured as previously described [49]. Primary WT PMCs and BMMCs were cultured in RPMI1640 cell culture medium (Invitrogen, #21,875–0991), with 15% FCS (Capricorn, #FBS-12A), 10 mM HEPES (pH 7.4), 50 units/ml penicillin, 50 mg/ml streptomycin, 100 µM β-mercaptoethanol, 30 ng/ml IL-3 from X63-Ag8-653 conditioned medium [50]. PMC cultures were supplemented in addition with approximately 20 ng/ml SCF from CHO culture supernatant, depending on its biological activity [49]. 

### Immortalized Mast Cell Lines

The PMC-derived MC cell line PMC-306 has been thoroughly described by Capellmann et al. [51]. Additionally, we used a well-established subclone of the human MC leukemia cell line HMC-1, known as HMC-1.1 and the murine bone marrow-derived MC line L138.8A. Information about the characteristics of these cell lines can be found elsewhere [52, 53]. 

### Primary and Immortalized Murine Hepatic Stellate Cells

Primary murine hepatic stellate cells (HSCs) from healthy WT mice aged 8–12 months were isolated and cultured using a conventional pronase/collagenase protocol as previously described [54]. The cellular and genetic characteristics of the immortalized murine hepatic stellate cell lines HSC-Col-GFP and GRX were published previously [55–57]. The cells were cultured in Dulbecco’s modified Eagle’s medium (DMEM) with high glucose (4.5 g/L, #D6171) supplemented with 10% FBS, 5 mM sodium pyruvate, 4 mM L-Glutamine solution, and 1 × penicillin/streptomycin solution. Cell passaging was performed using Accutase cell detachment solution (#A6964) from Sigma-Aldrich.

### Flow Cytometry

Approximately 0.5 × 10^6^ cells were washed in FACS buffer (PBS + 3% FCS + 0.1% sodium azide) and then stained with FITC-coupled anti-FcεRI (1:100, clone MAR-1, BioLegend, #134,306) or APC-coupled anti-CD117 (1:200, clone 2B8, BioLegend, #105,808), respectively, for 1 h at 4 °C in the dark. Afterwards, the cells were washed again in FACS buffer, resuspended in 150 µl of FACS buffer, and analyzed using a FACSCanto II (BD Biosciences).

### Transient Transfection of HMC-1.1 and PMC-306 Cells

HMC-1.1 cells were transfected at a density of 1.5 × 10^6^ cells/mL using Lipofectamine™ 2000 and 2 µg of plasmid DNA in 6-well plates according to the manufacturer’s instructions. For transient transfection of PMC-306 cells, cells were cultured in growth medium at a density of 0.5 × 10^6^ cells per mL in 2 mL medium per 6-well plate. After exchanging the medium (growth medium) and seeding at the desired density, the cells were pre-incubated for 1 h. Then, the pre-assembled transfection complexes in Opti-MEM, containing 12 µL Lipofectamine™ 2000 and 1 µg DNA, were added dropwise to the 2 mL cell suspension and incubated for 48 h.

### Luciferase Measurement

For luciferase measurement, cells were transiently transfected with (CAGA)_12_-MLP-Luc [58] (kindly provided by Dr. Peter ten Dijke, Department of Cell and Chemical Biology, Oncode Institute, Leiden University Medical Center, Leiden, The Netherlands) and (SBE)_2_-MLP-Luc [59] (kindly provided by Dr. Andreas Lux, formerly working at the Medical Faculty Mannheim, Heidelberg University, Heidelberg, Germany; Q-bios GmbH Biotechnology, Mannheim, Germany). After 24 h, the medium was exchanged with 0.1% BSA medium and cells were pre-incubated for 2 h. Then, TGF-β1 (1 ng/mL) or IL-3 medium supplement was added, and cells were further incubated for 20 h. Cells were pelleted (1,200 rpm, 5 min, 4 °C), washed once with ice-cold 1xPBS, resuspended in 100 µL of passive lysis buffer (Promega). 20 µL of lysed cells were added to 100 µL of luciferase substrate and luciferase measurement was performed according to the manufacturer’s instructions using a Victor Plate reader (Perkin Elmer). Luciferase activity was normalized to the protein content (DC kit, BioRad) of the corresponding samples and displayed as fold induction relative to the untreated samples.

### Staining of Mast Cells in Tissue

 CD117 in tissue sections was done following standard procedures. For toluidine blue , the tissue was fixed under different conditions as indicated. They were then dehydrated in an automatic device and embedded in paraffin. From these paraffin-embedded tissue blocks, 10 µm thick specimens were cut and deparaffinized/rehydrated using standard protocols. Manual staining of tissue slices using Toluidine Blue was performed for 5 min in a 1% Toluidine Blue stock solution diluted in a 1% NaCl solution at a pH of 2.0. Afterward, the tissue slices were washed, dehydrated, and embedded in DPX. Giemsa staining were done in the pathology department using routine procedures. After staining, selected sections were digitized using a NanoZoomer SQ digital slide scanner (C13140-21, Hamamatsu Photonics, Hamamatsu, Japan) and visualized using the NDP.view 2 software (U12388-01, Hamamatsu Photonics). 

### Concanavalin A Precipitation

Glycosylated proteins were precipitated using beads that were coupled to the lectin Concanavalin A (ConA, *Canavalia ensiformis*, cat. no. 234568, Calbiochem). Initially, the beads were washed three times with RIPA buffer. Subsequently, 100–200 µg of cellular proteins were added to the beads in a total volume of 500 μL and left to rotate overhead overnight at 4 °C. The next day, the complexes were washed three times with RIPA buffer, each for 15 min while rotating overhead at 4 °C. Finally, the precipitated proteins were resolved in a mixture of 20 μL RIPA buffer and 20 μL 4 × LDS sample buffer (ThermoFisher Scientific, #NP0007) along with 2 μL (1 M) dithiothreitol (DTT). The resolved proteins were then analyzed by Western blot analysis.

### RNA Isolation and RT-qPCR

RNA isolation and RT-qPCR were conducted as described previously [60]. The PCR conditions were set to 50 °C for 2 min, followed by 40 cycles of 95 °C for 15 s, and 60 °C for 1 min. All primer pairs used in this study are listed in Suppl. Table 2.

### Stimulation of Mast Cells

To stimulate MCs, cells were adjusted to a cell density of 1 × 10^6^ cells per mL and plated in six-well dishes. The cells were then centrifuged (1,200 g, 5 min, 4 °C) and resuspended in growth medium, starvation medium (growth medium without IL-3/SCF and with 10% FBS) or 0.1% BSA medium as indicated. If the experiment was performed in growth medium, cells were pre-incubated for 1 h after the medium exchange. If the experiment was performed in starvation/0.1% BSA medium, the cells were pre-incubated for 2 h. If cells were cultured with inhibitors, a pre-incubation of at least 30 min was performed before applying the indicated ligands. mTGF-β1 was used at a concentration of 1 ng/ mL for all applications. In experiments where IL-3 was used to stimulate cells, the serum supplement IL-3 was added to the culture at a concentration typically used for regular culture (as described above). After long term stimulations (24 h) an aliquot of cells was diluted (1:5), stained with Trypan Blue and manually counted. For harvesting, cells were washed with ice-cold PBS and lysed in the corresponding buffer for subsequent analysis on ice. All buffers contained protease and phosphatase inhibitors.

### Degranulation of Mast Cells

To induce the release of mast cell granule content, PMC-306 and BMMC were centrifuged (1,200 rpm, 5 min, 4 °C), plated at a density of 1 × 10^6^ cells per mL in growth medium, and loaded or not with IgE anti DNP (0.15 µg/mL) in the presence or absence of TGF-β1 (1 ng/mL) over night. Thereafter, cells were centrifuged (1,200 rpm, 5 min, 4 °C), washed once in modified Tyrode’s buffer [130 mM NaCl, 5 mM KCl, 1.4 mM CaCl_2_, 1 mM MgCl_2_, 5.56 mM Glucose, 0.1% BSA, 10 mM Hepes pH 7.4] resuspended in modified Tyrode’s buffer again and incubated for 15 min (37 °C). Then, DNP-HSA was added (40 ng/mL) and cells were incubated for 30 min (37 °C). Cells were pelleted (1,200 rpm, 5 min, 4 °C), washed with ice cold PBS and lysed using RIPA buffer. The supernatants were collected as well, and stored with cell lysates for Western blot analysis.

### Acetone Precipitation of Cell Culture Supernatants

To precipitate the proteins in cell culture supernatants, 500 µL of the supernatant was mixed with 2 mL of ice cold acetone and incubated for 1 h at −20 °C. The samples were then centrifuged at 10,000 rpm for 10 min at 4 °C, and the pellets were air-dried at 37 °C until the white pellet became transparent. The proteins were resuspended in 20 µL of RIPA buffer and 20 µL of LDS loading buffer with or without DTT (50 mM). Finally, the samples were analysed by Western blot.

### Separation of Nuclear and Cytosolic Fractions

After stimulation, the cells were sedimented (300 g, 5 min, 4 °C) and resuspended in 200 µL of PI (10 mM HEPES, 10 mM KCl, 0.1 mM EDTA, 1 mM DTT, 0.5% NP-40, pH7.4, including protease and phosphatase inhibitors). Samples were left on ice for 5 min and centrifuged (3,000 × g, 10 min, 4 °C). The supernatant (cytosolic fraction) was stored on ice and the pellet (nuclear fraction) was washed twice with ice-cold phosphate-buffered saline (PBS). Subsequently, the pellet was resuspended in 50 µL of PII [20 mM Hepes, 400 mM NaCl, 1 mM EDTA, 1 mM DTT, pH7.4, including protease and phosphatase inhibitors]. It was then sonicated (using an ultrasonic disintegrator, model UP100H from Hielscher Ultrasound Technology, Teltow, Germany) with three 15-s bursts on ice and left on ice for an additional 30 min. The lysate was centrifuged (15,000 × g, 15 min, 4 °C) and the resulting supernatant (nuclear fraction) was kept on ice. For Western blot analysis 20 µL of the cytosol fraction and 5 µL of the nuclear fraction were analysed.

### Sucrose Gradient Analysis

Sucrose density gradient analysis was conducted at 4 °C. Cells, either MCs or mouse HSC, were cultured in growth medium. MCs were centrifuged at 1,200 rpm for 5 min at 4 °C. The resulting cell pellets, as well as adherent HSC, were washed three times with ice-cold phosphate-buffered saline (PBS). For the MC pellets, 0.5 mL of sodium carbonate buffer (500 mM, pH 11.0) was added. As for the adherent cells, they were scraped into 0.5 mL of sodium carbonate buffer. The resuspended cells were homogenized in a Hielscher ultrasonic disintegrator as described above. Then, 1.5 mL of sodium carbonate buffer was added and the homogenates were adjusted to 45% sucrose using 2 mL of 90% sucrose in MES-buffered saline (25 mM MES, 150 mM NaCl, pH 6.5). The homogenates were placed at the bottom of an ultracentrifuge tube (Beckman Coulter). Two solutions of 35% and 5% sucrose (4 mL each) were carefully layered sequentially on top of the 45% sucrose solution. The tubes were then subjected to ultracentrifugation at 39,000 rpm in a Beckman OptimaTM L-70 K centrifuge using a SW 40 Ti rotor for 20 h. After centrifugation, twelve individual 1-mL fractions were collected from the top of the tubes. 50 μL of each fraction was analyzed by Western blot in reducing conditions (50 mM DTT).

### Western Blot Analysis

Western blot analysis was conducted as previously described [61]. After blotting, Ponceau S stain was used to confirm the proper transfer of proteins to the membrane, and nonspecific binding sites were blocked with 5% (w/v) non-fat milk powder in Tris-buffered saline containing 0.1% Tween 20 (TBST). For the detection of individual proteins, the primary antibodies were diluted in 2.5% (w/v) non-fat milk powder in TBST. All antibodies used in this study are listed in Suppl. Table 3. Primary antibodies were visualized using anti-mouse, anti-rabbit, anti-rat or anti-goat IgG secondary antibodies (all from Santa Cruz Biotech., Santa Cruz, CA, USA) with the SuperSignal chemiluminescent substrate (Pierce, Bonn, Germany).

### Human Liver Samples

Human liver samples were obtained from the RWTH centralized Biomaterial Bank (RWTH cBMB; https://www.cbmb.ukaachen.de/) at the Medical Faculty in Aachen. The use of these samples for our purposes was approved by the scientific management of the RWTH cBMB and the Ethics Committee of the Medical Faculty of Medicine (EK206/09).

### Data Analysis and Statistical Analysis

Unless stated otherwise, the results obtained from a minimum of three independent experiments were expressed as the mean of the group plus standard deviation. This was calculated using Microsoft Excel (Microsoft Corporation). A Student’s *t*-test was used to compare two groups, while one-way analysis of variance (ANOVA) was used to compare means of more than two groups, depending on whether the data followed a parametric or non-parametric distribution. *p*-values less than 0.05 were considered statistically significant (* *p* < 0.05, ** *p* < 0.01, *** *p* < 0.001, ns: > 0.5). The number of independent biological replicates per experiment is specified in the figure legends.

## Results

### Mast Cells are Located in Fibrotic Liver Areas

**Fig. 1:**
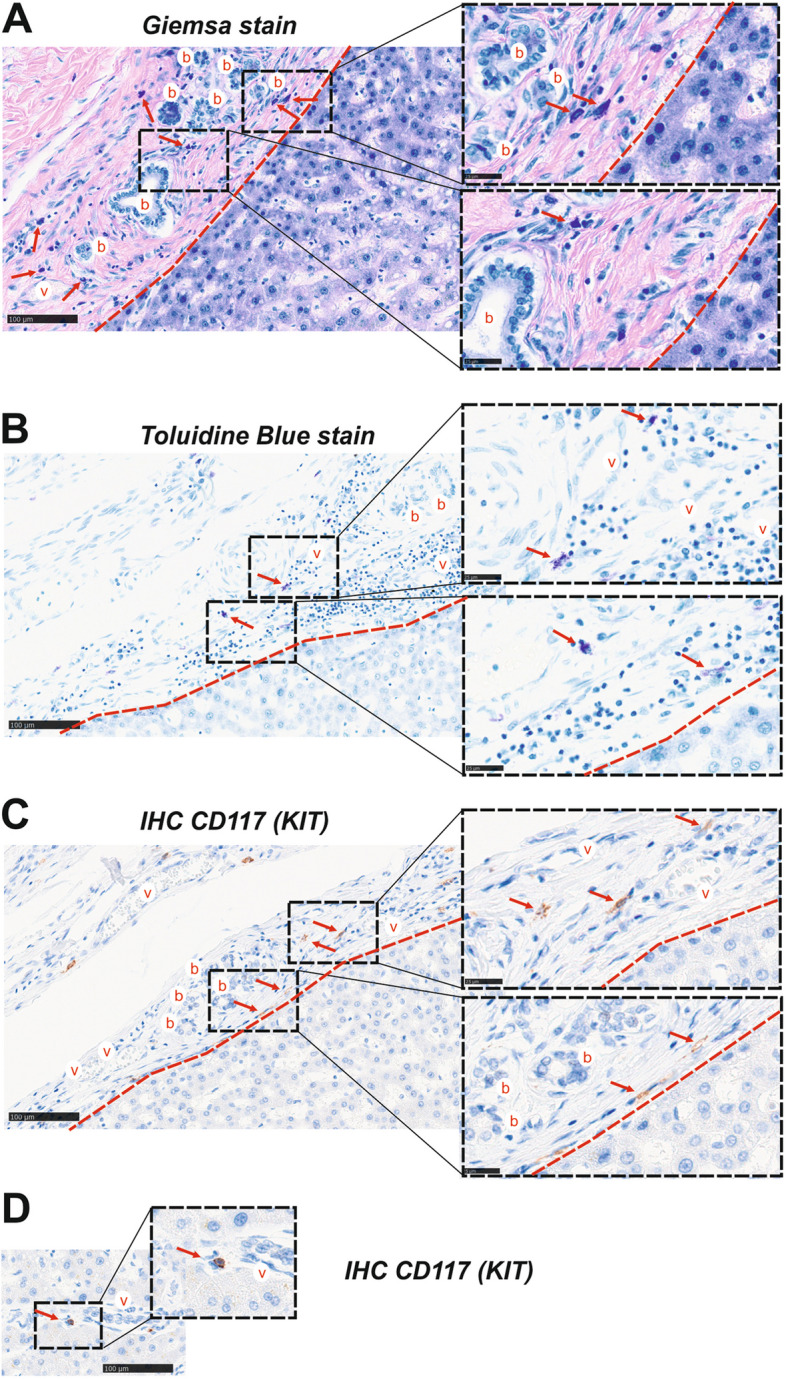
MCs are located in fibrotic areas in biopsies of patients suffering from primary sclerosing cholangitis (PSC). Formalin fixed, paraffin embedded tissue slices (10 µm) of liver biopsies from patients admitted to the hospital for liver transplantation and diagnosed with PSC, were stained for MC detection. Three methods were used: (**A**) Giemsa stain, (**B**) Toluidine Blue, and (**C**) immunohistochemical detection of CD117 (KIT). A total of three patients with PSC were included in this study. It is important to note that MCs were found in the rearranged fibrotic areas near the bile ducts, adjacent to the residual liver tissue, using all three detection methods. The dashed red line indicates the boundary between the fibrotic tissue and parenchyma. **D** Rarely occurring MCs in the parenchyma found in close proximity to a vessel. b: bile duct; v: vessel

It has been shown that MCs infiltrate the liver following injury caused by various conditions, including primary sclerosing cholangitis (PSC), PSC associated with inflammatory bowel disease (PSC-IBD), primary biliary cholangitis (PBC), non-alcoholic steatohepatitis (NASH), and biliary atresia [27, 62, 63]. This study focuses on the presence of MCs in human biopsies from patients with advanced PSC undergoing liver transplantation (Fig. 1). MCs were found in fibrotic areas, such as portal fields, using Giemsa, Toluidine Blue stain, and immunohistochemical detection of KIT (CD117). This indicates that MCs are typically found in fibrotic areas within the portal fields and bridging septae of fibrotic human liver tissue. These findings confirm that MCs can be identified in the human liver both histochemically through heparin (Giemsa, Toluidine Blue) and immunohistochemically using a CD117-specific antibody (Fig. 1A-D). This suggests that MCs in the diseased human liver are recruited to fibrogenic areas, which are typically characterized by enhanced TGF-β1 activity [64].

### Mast Cells are Characterized by a High Type I/Type II Receptor Ratio and Low Type III TGF-β Receptor Expression

The localization of MCs in fibrotic areas in a TGF-β1 rich environment implies their involvement in TGF-β1 signalling events driving fibrogenesis. Therefore, we aimed to determine the modes of responses and their regulation in MC. To do so we conducted a comparative analysis using different MC cell lines and primary BMMC, which are commonly used for analysing TGF-β1 signal transduction. In previous studies the analysis of TGF-β receptor expression in different types of MCs, resulted in contradictory findings for individual receptors [32, 34, 45]. Since the receptor expression pattern determines the signalling capabilities of cells, we analysed TGF-β receptor expression in primary BMMCs as well as L138.8A and PMC-306 MC lines. As a control, we also analysed receptor expression in primary murine HSCs and the immortalized HSC lines HSC-Col-GFP and GRX (Suppl. Figure 1A,B).

**Fig. 2:**
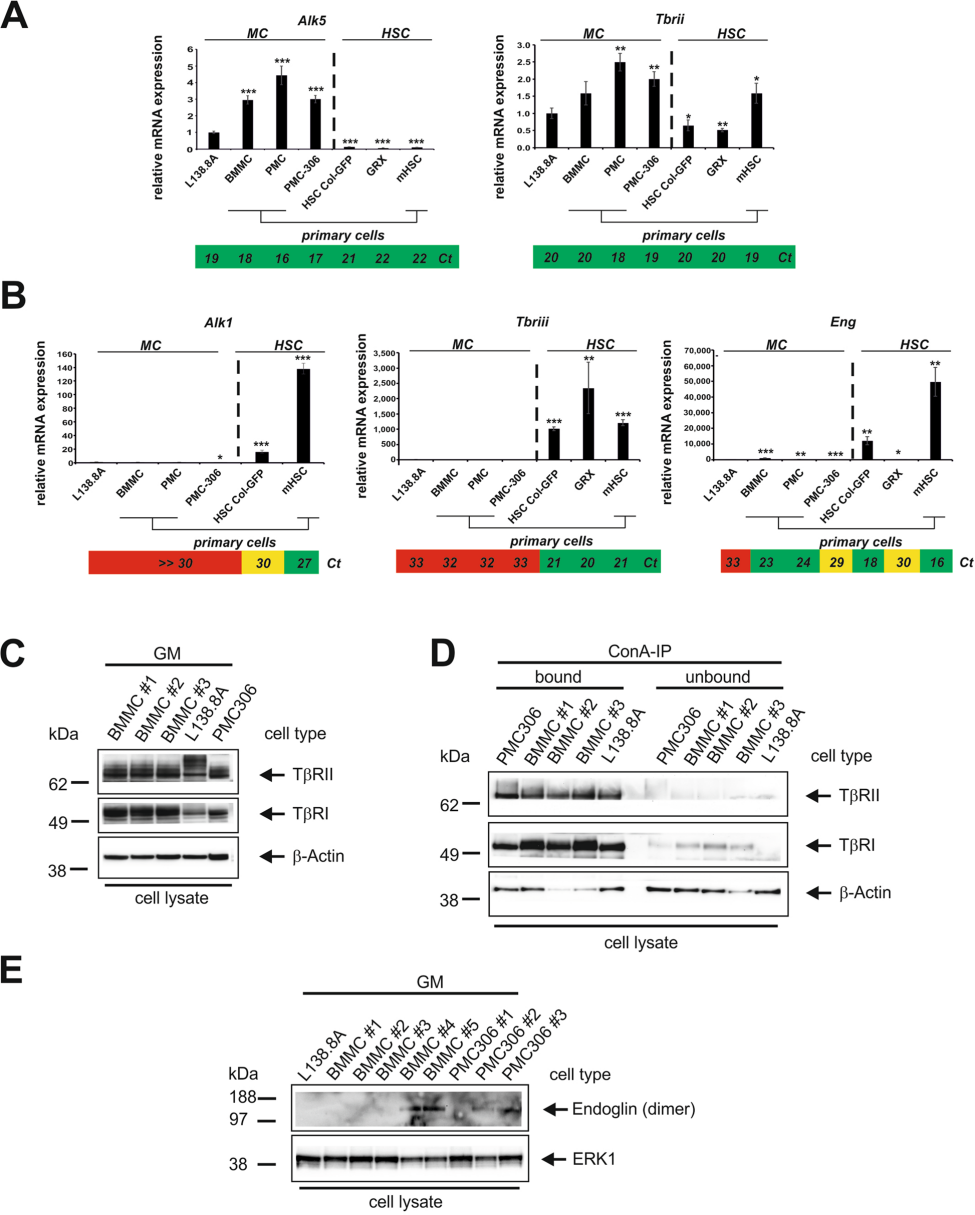
MCs express a high ratio of TGF-β core receptors type I/II and nearly no co-receptors. MCs and hepatic stellate cells (HSC) were cultured under standard growth conditions.** A**,**B** RNA was isolated and subjected to qPCR analysis. **A** Determination of TGF-β type I receptor (*Alk5*, *left panel*) and the TGF-β type II receptor (*Tbrii, right panel*). **B** Measurement of the „alternative “ TGF-β type I receptor *Alk1* (*left panel*), the TGF-β type III co-receptors *Betaglycan* (*Tbriii, middle panel*), and the long splice variant of the TGF-β type III co-receptor *Endoglin* (*Eng*, *right panel*), The mean *C*_*t*_ values are indicated below the qPCRs to provide a rough estimate of the overall expression level. Expression of each receptor was normalized to β-actin and displayed as fold induction relative to the expression level of L138.8A. (n = 3; ± SD; *p < 0.05; ** p < 0.01; *** p < 0.001). **C-E** Proteins of the indicated cells grown in steady state conditions were extracted and subjected to immunoblot analysis. **C** Protein expression of TGF-β type I (ALK5, TβRI) and type II (TβRII) receptor under reducing conditions. **D** Precipitation with concanavalin A (ConA) and Western blot were used to detect the TGF-β type I (ALK5, TβRI) and type II (TβRII) receptor-protein expression exploiting the *N*-glycosylation of the mature proteins. **E** Western blot detection of the dimeric TGF-β type III co-receptor Endoglin under non-reducing conditions. Equal loading of proteins was confirmed by re-probing the membranes with a β-Actin-specific antibody (**C**,**D**), or an ERK1/2-specific antibody (**E**). GM: growth medium

Using RT-qPCR we detected mRNA expression of the core receptors type I (*Alk5*) and type II (*Tbrii*) in the tested primary and immortalized MCs (Fig. 2A) showing substantially elevated *Alk5* mRNA expression in all tested MCs compared to HSCs. We also analysed the expression of the “alternative” type I TGF-β1 receptor, *Alk1* [65], which mediates SMAD1/SMAD5 activation and ID protein expression in endothelial cells [66]. *Alk1* expression was undetectable in MCs, but clearly detectable in HSCs as previously published [67] (Fig. 2B, *left panel*). The mRNA expression of receptor II (*Tbrii*) was comparable among the analysed cell types. In contrast to the core receptors, the co-receptors type III, *Betaglycan*, and *Endoglin* (*Eng, CD105*) were differentially expressed (Fig. 2B). MCs did not show detectable transcripts for *Betaglycan*, which was highly expressed in HSCs (Fig. 2B, *middle panel*). *Endoglin* was expressed in primary MCs but not in the corresponding cell lines. In contrast, *Endoglin* mRNA was highly expressed in HSCs, except in the GRX cell line (Fig. 2B, *right panel*). As expected from the transcript analysis, the core receptor proteins were expressed in primary MCs (BMMC) and MC lines (L138.8A, PMC-306) (Fig. 2C). The presence of both discrete and smeared bands indicated the expected post-translational glycosylation of receptors with significant differences among the analysed cells. ConA precipitation confirmed the presence of *N*-linked oligosaccharides, as the receptor proteins are bound and precipitated by this lectin (Fig. 2D). Proof of Endoglin expression was challenging. However, we detected Endoglin in both in BMMC and PMC306 (shown here for the dimer, in the absence of DTT) (Fig. 2E).

In contrast to the ConA-precipitated receptors, which displayed a very homogeneous migration pattern, the total receptors in the cell lysate showed a more heterogeneous migration pattern, particularly in the cell lines. This suggests a different glycosylation pattern in primary cells and cell lines (Suppl. Figure 1B). This difference was also observed for the stem cell factor receptor KIT (Suppl. Figure 1B). Additionally, the difference in mRNA expression of *Alk5* and *Endoglin* in MCs compared to HSCs was confirmed at the protein level (Suppl. Figure 1B). Based on the receptor expression we concluded that: i) the core receptors are expressed at high levels, ii) there is a high ratio of type I to type II receptors, different from that found in HSCs, iii) the accessory receptors necessary for fine-tuning responses are either not expressed (*Betaglycan*) or expressed only in primary cells (*Endoglin*), and iv) there is no indication of an alternative signalling pathway via* Alk1*.

### TGF-β Receptors are Localized to Low and High Density Membrane Compartments

**Fig. 3:**
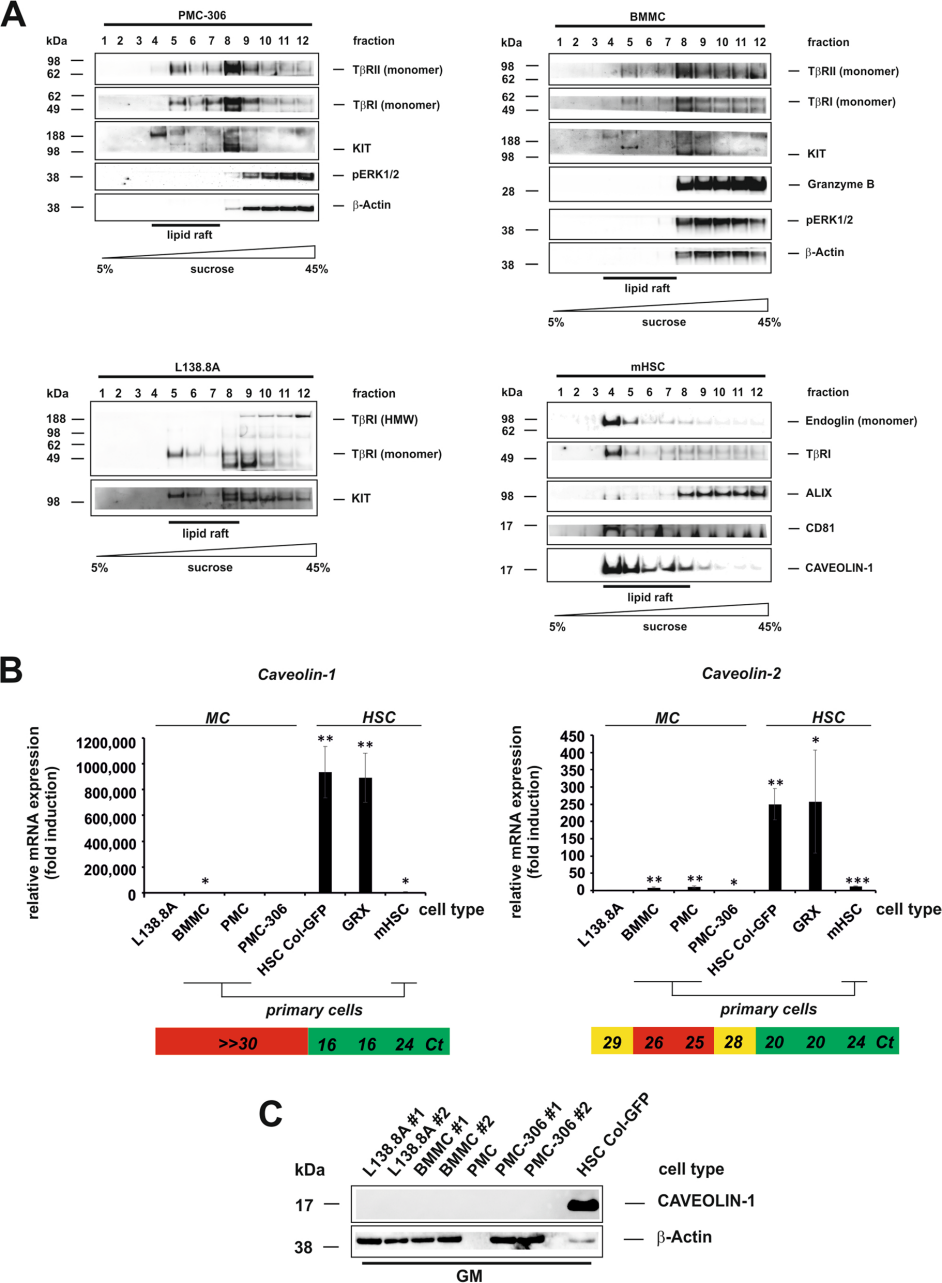
TGF-β receptors are located in high and low density membrane microdomains. **A** To differentiate between cholesterol-rich/low-density (caveolae) and high density (clathrin-coated pits) microdomains, sucrose density gradient centrifugation was performed. Cells were cultured in growth medium, harvested, sheared, sonicated and subjected to a sucrose gradient (5% to 45%) centrifugation for 20 h. Samples of PMC-306 (*upper left*), BMMC (*upper right*), L138.8A (*lower left*) and primary HSC (*lower right*), taken from the individual 12 fractions were analyzed by Western blot using the indicated marker proteins. **B**,**C** mRNA (**B**) and protein (**C**) expression analysis of *Caveolins* in MCs and hepatic stellate cells as a control, cultured in basal conditions were performed. **B** In RT-qPCR experiments specific primers for *Caveolin-1* (*left panel*) and *Caveolin-2* (*right panel*) were used. Expression of *Caveolins* was normalized to *β-actin* and displayed as fold induction relative to the expression level of L138.8A. (n = 3, ± SD; * *p* < 0.05, ** p < 0.01, *** p < 0.001). The mean *Ct* values are indicated below the RT-qPCRs to roughly estimate the overall expression level.** C** Analysis of cellular proteins by Western blot analysis using a CaveolinAVEOLIN-1 specific antibody. To demonstrate equal protein loading, the membrane was re-probed with a β-Actin specific antibody. GM: growth medium

The distribution of receptors to membrane compartments determines their mode of signalling [68]. Therefore, we conducted sucrose gradient analysis (Fig. 3). Western blot analysis of gradient fractionated proteins revealed that type I and II receptors are primarily distributed in high-density fractions (Clathrin-coated pits), but also in low-density fractions (Caveolae) in all analysed MCs and HSCs (Fig. 3A). In MCs, KIT exhibited the same distribution in both microdomains, while the effector Granzyme B, activated ERK1/2, and β-Actin exclusively resided in the high density Clathrin fractions. Surprisingly, CAVEOLIN-1 was not detected in MC fractions, despite its prominent presence in the low-density (Caveolin) fractions of HSCs which contain Endoglin (sample Nr. 4–8, Fig. 3A). It has been described that *Caveolin-1* is expressed in BMMCs and plays a role in bacterial entry into MCs [69]. However, we were unable to detect *Caveolin-1* in our analyses. Therefore, we examined *Caveolin* mRNA expression and found that *Caveolin-1* is absent in MCs, whereas *Caveolin-2* is expressed in primary BMMCs and PMCs, but at very low levels in MC cell lines (Fig. 3B, *upper panels*). The mutual absence of CAVEOLIN-1 was also confirmed by Western blot analysis. Expression analysis of CAVEOLIN-1 in HSC confirmed the functionality of the primers and antibody used (Suppl. Figure 1A, Fig. 3B,C). Thus, TGF-β receptors are localized in low and high-density membrane domains in all analysed MCs, similar to KIT. Low-density domains in MCs do not contain Caveolin-1, however, contain highly glycosylated receptors (TGF-β-receptors and KIT) in MCs and HSCs.

### TGF-β1/ALK5 Mediates Activation of Two SMAD Signalling Pathways with Different Kinetics

**Fig. 4:**
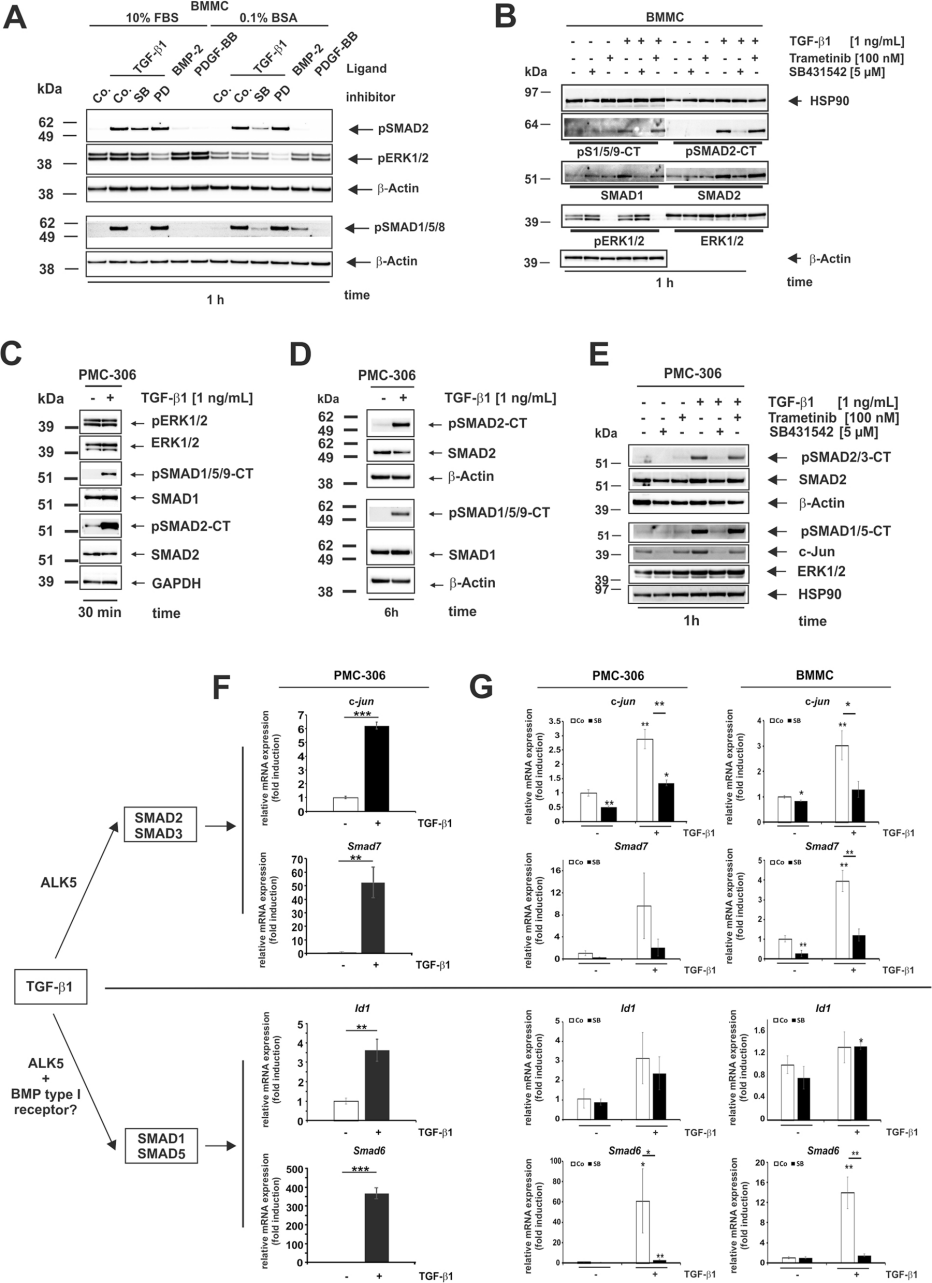
TGF-β1-mediated activation of SMAD2 and SMAD1/5 pathways and corresponding target genes in MC. **A**,**B** BMMCs were starved overnight, pre-incubated in the absence or presence of specified inhibitors for 1 h and subsequently stimulated either in starvation medium (10% FBS) or stimulation medium (0.1% BSA) with the indicated ligands for 1 h: TGF-β1 (1 ng/mL), BMP-2 (25 ng/mL), PDGF-BB (25 ng/mL), SB431542 (SB, 5 µM), PD98059 (PD, 10 µM), Trametinib (100 nM). Cellular proteins were analysed by Western blot using the indicated antibodies. Equal protein loading was monitored by detecting β-Actin **A**,**B** and HSP90 (**B**). (*n* = 3). **C**,**D** PMC-306 were cultured in growth mediumand stimulated or not with TGF-β1 (1 ng/mL) for 30 min (**C**) or 6 h (**D**). Cellular proteins were analysed by Western blot using the indicated antibodies. Equal protein loading was demonstrated by re-probing the membrane with a GAPDH (**C**) or a β-Actin (**D**) specific antibody. **E** PMC-306 were cultured in growth medium, pre-incubated with or without SB431542 (5 µM), Trametinib (100 nM), and stimulated or not with TGF-β1 (1 ng/mL) for 1 h**.** Cellular proteins were analysed by Western blot using indicated antibodies. Equal protein loading was demonstrated by re-probing the membrane with a HSP90 specific antibody.** F** PMC-306 were cultured in starvation medium, stimulated with TGF-β1 (1 ng/mL) for 90 min where indicated, followed by RNA extraction and qPCR analysis of the SMAD1/SMAD5 target genes *Id1 and Smad6* (*right panels*) or the SMAD2/SMAD3 target genes *c-jun* and *Smad7* (*left panel*). Expression of *Id1, Smad6, c-jun* and *Smad7* was normalized to *β-actin* and displayed as fold induction relative to the untreated control. (*n* = 3; ± SD; ** p < 0.01; *** p < 0.001). **G** PMC-306 (*left panels*) or BMMC (*right panels*) cells were cultured in growth medium, pre-incubated in the presence or absence of SB431542 (5 µM) and then stimulated with TGF-β1 (1 ng/mL) for 6 h where indicated. RNA was extracted and RT-qPCR was performed for the detection of *c-jun*, *Smad7, Id1* and *Smad6*, normalized to *β-actin* and displayed as fold induction relative to the untreated control. (*n* = 3; ± SD; * *p* < 0.05; ** p < 0.01)

MC effectors are directly regulated by MAP kinases and SMAD mediators in response to TGF-β1 [47, 70]. To analyse the activation of signalling intermediates in response to TGF-β1, we first stimulated BMMCs for 1 h with TGF-β1 (Fig. 4A). These experiments were conducted either in medium containing 10% FBS or in medium containing 0.1% BSA. As expected, the activation of ERK1/2 was reduced when the medium contained 0.1% BSA instead of 10% FBS. BMP-2 was used as control stimulus and had no effect on SMAD activation (Fig. 4A). TGF-β1 induced SMAD2 phosphorylation, and this activation was reduced by the ALK5 inhibitor SB431542 (SB) (Fig. 4A,B).

Surprisingly, we found i) an activation of SMAD1/5, and ii) this was also dependent on ALK5 activity (Fig. 4A,B). Inhibition of ERK1/2 activation using the MEK inhibitor PD98059 or Trametinib did not affect the pSMAD signal. As expected, PDGF-BB did not induce ERK1/2 activation due to the absence of the PDGF receptor-β (data not shown). These pilot experiments demonstrated that medium including 10% FBS was suitable to analyse the respective TGF-β-responsive pathways in BMMCs. Therefore, TGF-β1 stimulation of the recently described cell line PMC-306 [51] was first performed directly in growth medium (including 10% FBS) for 30 min and 6 h. (Fig. 4C,D). Similar to BMMCs, TGF-β1 caused activation of SMAD2 and SMAD1/5 in PMC-306 cells after 30 min, and this activation was still apparent after 6 h. These activation events were both blocked by the inhibitor SB431542 after 1 h of stimulation (Fig. 4E). The activation of SMAD1/5 and SMAD2 could be confirmed by upregulation of the corresponding target genes *Id1/Smad6* (SMAD1/5) and *c-jun*/*Smad7* (SMAD2/3) after 90 min in PMC-306 (Fig. 4F) and dependency on ALK5 after 6 h in PMC-306 (Fig. 4G, *left panels*) and BMMC (Fig. 4G, *right panels*).

We also confirmed the responsiveness to TGF-β1 in commonly used cell lines of human (HMC1.1) and mouse origin (L138.8A) (Suppl. Figure 2). In both cell lines, TGF-β1 activated SMAD2 (Suppl. Figure 2A,B). Additionally, we observed a weak activation of SMAD1/5 but only in HMC1.1 (Suppl. Figure 2C). SMAD activation of both pathways was sensitive to the ALK5 inhibitor SB431542. Both inhibitors, SB431542 and PD98059, were specific for their targets, except for Dorsomorphin (DM), which does not affect SMAD, but ERK1/2 activation (Suppl. Figure 2A-C). DM was initially used to analyse the involvement of BMP type I receptors in TGF-β1-mediated SMAD1/5 activation. It was shown that SMAD3 is expressed and involved in MC growth and *Mcpt6* expression [37, 71]. However, despite the expectation that the antibodies used for detecting SMAD2/pSMAD2 would cross-react with SMAD3/pSMAD3 (due to the high sequence homology between SMAD2 and SMAD3), we were unable to detect SMAD3/pSMAD3. Therefore, we analysed mRNA expression levels of individual *Smad* mRNAs, including *Smad3*, in MCs (Suppl. Figure 2D). This analysis revealed that the mRNA levels for *Smad2* and *Smad1/Smad5* in MCs are comparable to HSCs, while *Smad3* mRNA is strongly underrepresented in MCs, especially in PMC-306 cells compared to HSCs (Suppl. Figure 2D). Thus we conclude that MCs respond to TGF-β1 with the activation of SMAD2 and SMAD1/5, and both pathways depend on ALK5 activity.

### TGF-β1/SMAD-Mediated *Smad6/Id1 and c-jun/Smad7* Expression is Directly Regulated and Reduced by IL-3

**Fig. 5:**
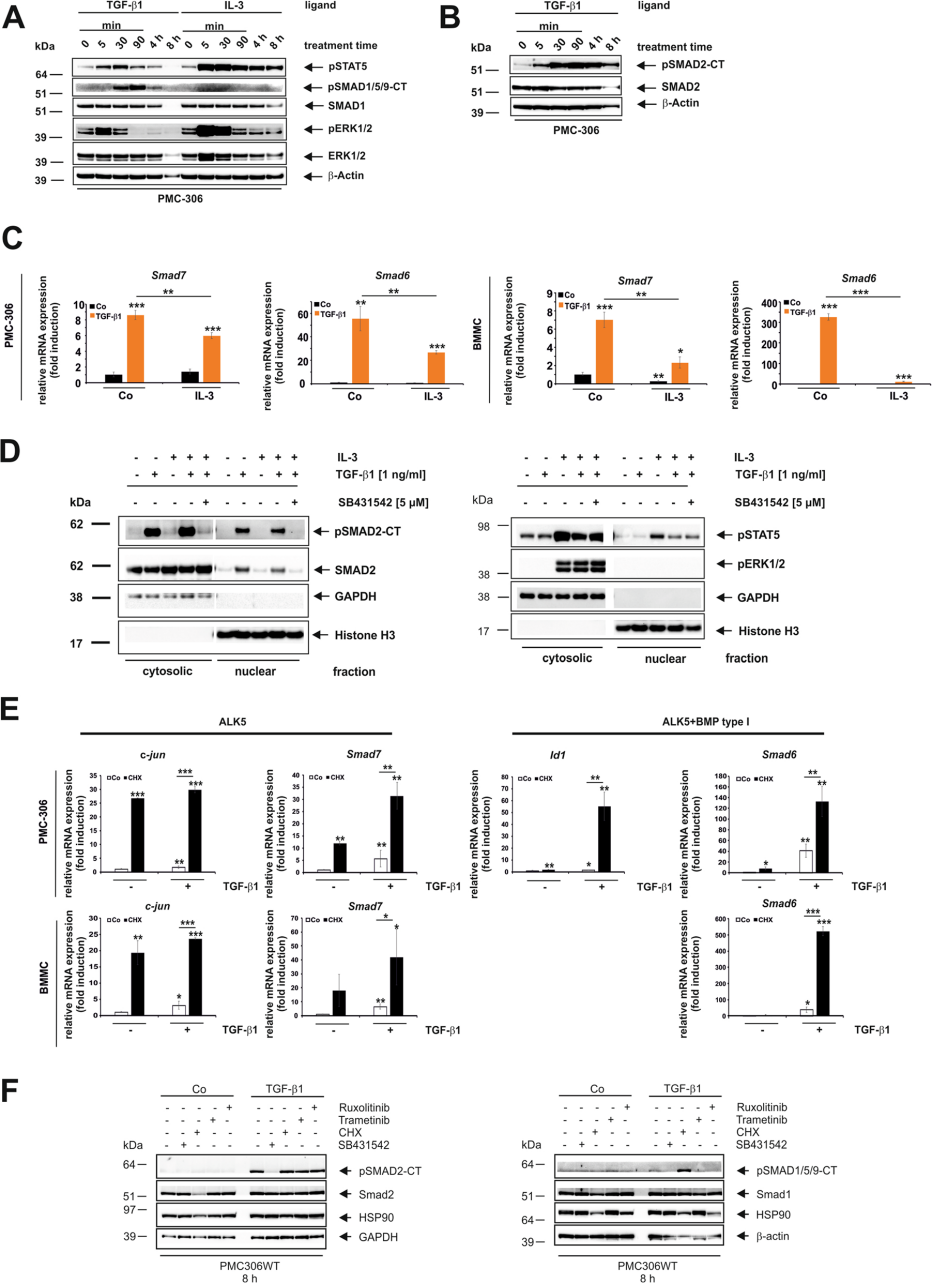
Impact of IL-3 on short term TGF-β1-mediated activation of intermediates and target gene expression. **A**,**B** PMC-306 cells were cultured in starvation medium, then stimulated or not with TGF-β1 (1 ng/mL) or IL-3 for the indicated times. Cellular proteins were analysed by Western blot and the detection of β-Actin was used to demonstrate equal protein loading. (**C**) PMC-306 (*left panels*) or BMMC (*right panels*) were cultured in starvation medium and stimulated or not with TGF-β1 (1 ng/mL), serum supplement IL-3 or a combination thereof for 6 h. RNA was extracted and RT-qPCR was performed for the detection of *Smad7* (*left panels*) or *Smad6* (*right panels*). Expression of *Smad6* and *Smad7* was normalized to *β-actin* and displayed as fold induction relative to the untreated control. (*n* = 3; ± SD; * *p* < 0.05, ** p < 0.01, *** p < 0.001) (**D**) PMC-306 cells were cultured in starvation medium, pre-incubated for 1 h in the presence or absence of SB431542 (5 µM) and then stimulated with TGF-β1 (1 ng/mL), IL-3 or a combination thereof for 30 min where indicated. Thereafter, cytosolic and nuclear fractions were isolated and analyzed by Western blot using the depicted antibodies. Purity and identity of the cellular fractions were monitored by GAPDH (cytosol) or Histone H3 (nucleus), respectively. **E** PMC-306 (*upper panels*) and BMMC (*lower panels*) cells were cultured in growth medium, pre-incubated for 1 h in the presence or absence of cycloheximide (CHX, 10 µg/mL) and then stimulated or not with TGF-β1 (1 ng/mL) for 6 h. RNA was extracted and RT-qPCR performed for the detection of *c-jun*/*Smad7* (*left panels*, ALK5 targets)*, Id1*/*Smad6* (*right panels,* ALK5 + BMP type I). Expression of *c-jun*, *Smad7*, *Smad6* and *Id1* was normalized to *β-actin* and displayed as fold induction relative to the untreated control. (*n* = 3; ± SD; * *p* < 0.05, ** p < 0.01, *** p < 0.001). **F** PMC-306 cells were cultured in growth medium, pre-incubated for 1 h in the presence or absence of CHX (10 µg/mL), SB431542 (5 µM), Trametinib (100 nM), Ruxolitinib (500 nM) and then stimulated or not with TGF-β1 (1 ng/mL) for 8 h. Cellular proteins were analysed by Western blot and the detection of GAPDH, β-Actin and HSP90 was used to demonstrate equal protein loading

Since TGF-β1 mainly induces signalling events involved in the regulation of anti-proliferation/pro-differentiation, we sought to investigate whether there is cross-talk between signalling processes induced by the proliferative/pro-survival factor IL-3 and TGF-β1 in PMC-306 and BMMC (Fig. 5). Kinetic analyses in PMC-306 cells revealed that TGF-β1 caused a delayed and only transient activation of SMAD1/5 (pSMAD1/5/9-CT) compared to prolonged SMAD2 (pSMAD2-CT) activation (Fig. 5A,B). Additionally, TGF-β1 induced a brief and transient activation of STAT5 and ERK1/2 for up to 30 min, followed by a decrease in ERK1/2 steady state activation. IL-3 led to immediate strong phosphorylation of STAT5, and ERK1/2 without directly affecting SMAD activation (Figs. 5A and D). Interestingly, IL-3 reduced TGF-β1-mediated target gene expression of *Smad7* and *Smad6* in both PMC-306 (Fig. 5C, *left panels*) and BMMCs after 6 h (Fig. 5C, *right panels*). To determine if IL-3 affects target gene expression by SMAD activation/nuclear translocation, we performed separation of nuclear and cytosolic compartments (Fig. 5D *left panel*). TGF-β1 increased the phosphorylation and nuclear translocation of SMAD2. IL-3 did not affect the activation or translocation of SMAD2 (Fig. 5D, *left panel*). Treatment controls for IL-3 are shown in Fig. 5D, *right panel* via activation of pSTAT5 and pERK1/2.

The decrease in STAT5 activation upon combined TGF-β1/IL-3 stimulation was not consistently observed (Fig. 5D, *right panel*). Furthermore, blocking secondary responses by inhibiting protein synthesis using CHX showed that gene responses mediated by TGF-β1 were still present and even increased in PMC-306 (*upper panels*) and BMMC (*lower panels*) (Fig. 5E). The increase in target gene expression upon CHX treatment is coupled with stronger SMAD1/5 activation (Fig. 5F, *right panel*), while SMAD2 activation remains unaffected (Fig. 5F, *left panel*). These results confirm the direct impact of TGF-β1-mediated signalling on *c-jun, Smad7, Smad6*, and *Id1*, and show that these responses are reduced by IL-3. However, there is no direct effect on SMAD2 activation or nuclear translocation. Moreover, the use of CHX suggests that translational processes in MCs are induced (mutually by IL-3) causing dampening of TGF-β1-induced *c-jun*, *Smad6*, *Smad7*, and *Id1* transcription.

### Long-Term TGF-β1 Responses in Mast Cells Lead to SMAD2 Activation, *Smad7* Expression and Reduced Cell Viability

**Fig. 6:**
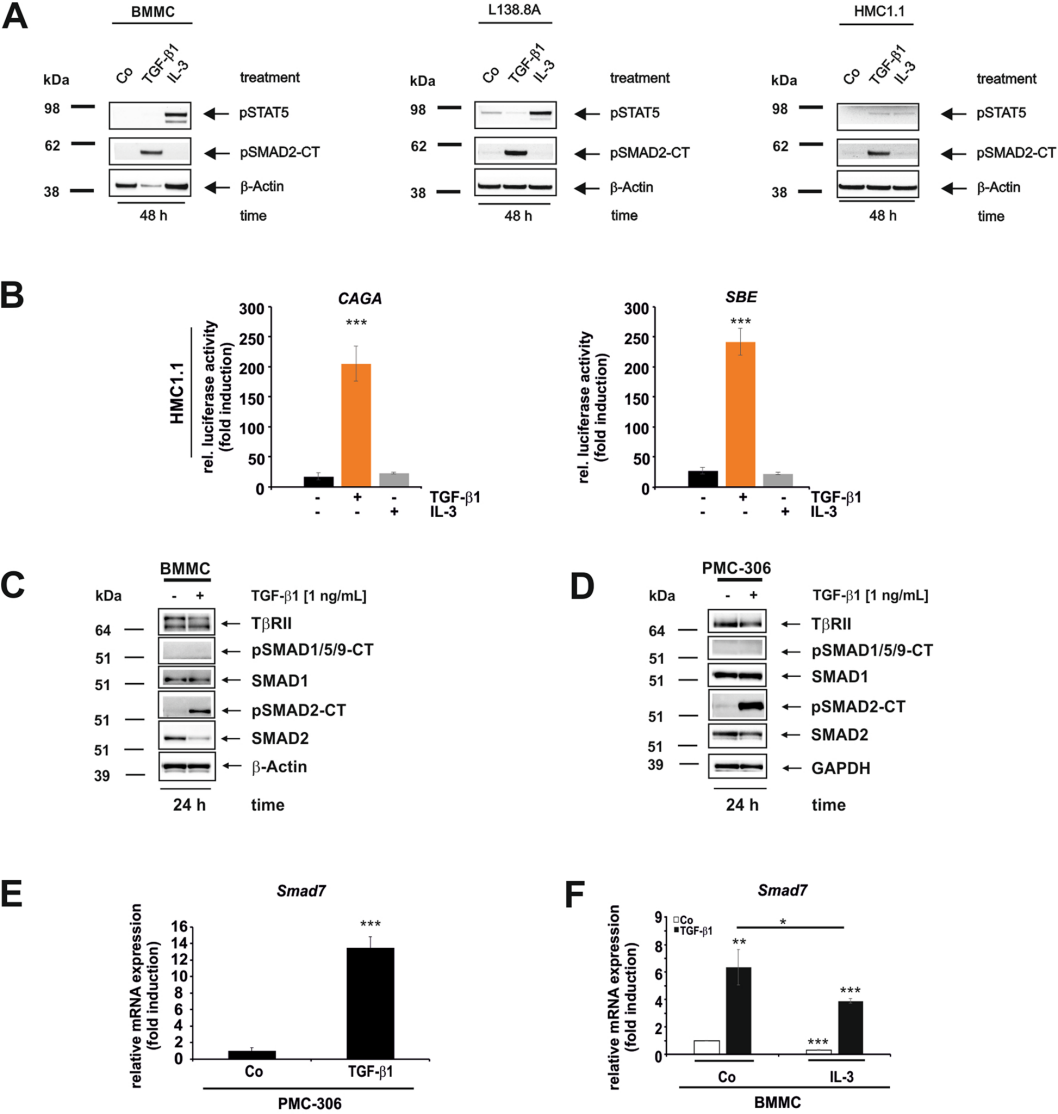
TGF-β1-mediated pathway selectively activates intermediates and target genes in MC. **A** To monitor the persistence of TGF-β1-mediated SMAD activation, primary MCs (BMMC, *left panel*) and permanent MCs (L138.8A, *middle panel*; HMC1.1, *right panel*) were cultured overnight in starvation medium before the addition of TGF-β1 (1 ng/mL) or IL-3 for 48 h. Proteins were then isolated and analysed by Western blot for the activation of SMAD2 and STAT5. Equal loading was confirmed by re-probing the membrane with a β-Actin specific antibody. **B** HMC1.1 cells were transiently transfected with the SMAD3 responsive luciferase reporters CAGA_12_-Luc (*left*) or SBE_2_-Luc (*right*) using Lipofectamine.™ 2000 (ratio 2:6). Cells were starved overnight before the addition of TGF-β1 (1 ng/mL) or IL-3 for 24 h. Cells were harvested, and luciferase activity was determined and normalized to the protein content of the individual samples. The results were displayed as fold induction relative to the untreated control. (n = 3; ± SD; *** p < 0.001). **C**,**D** BMMCs (**C**) or PMC-306 cells (**D**) were cultured in growth medium and stimulated with or without TGF-β1 (1 ng/mL) for 24 h. Cellular proteins were harvested and analysed for the indicated marker proteins using Western blot. Equal protein loading was confirmed by re-probing the membranes with a β-Actin (**C**) or a GAPDH (**D**) specific antibody. **E** PMC-306 cells were cultured in growth medium with or without TGF-β1 (1 ng/mL) for 24 h, RNA was extracted and RT-qPCR for the detection of *Smad7* was performed. **F** BMMCs were cultured in starvation medium with or without TGF-β1, IL-3 or a combination thereof for 24 h. RNA was extracted and RT-qPCR was performed to detect *Smad7*. The expression of *Smad7* was normalized to *β-actin* and displayed as fold induction relative to the untreated control. (*n* = 3; ± SD; * *p* < 0.05, ** p < 0.01, *** p < 0.001)

To determine whether the initial rapid TGF-β1-induced SMAD responses persist for more than 6 h and potentially contribute to the continuous production of effector (MCPT1, as described below) in MCs, we conducted a long-term activation experiment (Fig. 6).

It is well-established that TGF-β1 strongly affects the viability of MCs [72]. In pilot experiments we first determined appropriate culture conditions for long term stimulation (Suppl. Figure 3). Using flow cytometry analysis to examine starved cells stimulated with IL-3, TGF-β1 or both (Suppl. Figure 3A) we found an increase in apoptotic cells mediated by TGF-β1 even in the presence of IL-3. Additionally, we determined the numbers of cells cultured in growth medium (with and without TGF-β1) for 24 h or starvation medium in the absence or presence of IL-3/SCF with or without TGF-β1 (Suppl. Figure 3B). These analyses confirmed that TGF-β prevents the survival of MCs, impacting their viability (Suppl. Figure 3A,B). In IL-3- and FBS-containing growth conditions, the addition of TGF-β1 only had a subtle effect on the viability of BMMCs and PMC-306 cells (Suppl. Figure 3B, *upper panel*). The shift to starvation conditions significantly reduced the viability of cells, particularly for PMC-306 (Suppl. Figure 3B, *lower panel*). Confirming previous data [51], PMC-306 cells exhibited higher sensitivity to growth factor deprivation, as evidenced by cell count and corresponding Western blot analysis of cellular proteins (Suppl. Figure 3B,C). Therefore, long-term analysis of PMC-306 was only performed in growth medium.

Stimulation with TGF-β1 for 48 h led to the phosphorylation of SMAD2 in primary BMMCs (Fig. 6A, *left panel*), L138.8A (Fig. 6A, *middle panel*) and the human cell line HMC1.1 (Fig. 6A, *right panel*). IL-3 did not directly affect SMAD2 activation, but it provoked an increase in STAT5 activation in the murine cells. To analyse SMAD3 activation, we first demonstrated successful transfection of cells using an EGFP reporter (Suppl. Figure 3D). Thereafter, HMC1.1 was transiently transfected with the reporter plasmids CAGA_12_-Luc and SBE_2_-Luc, which contain SMAD3 binding elements from the *SERPINE1* and *JUNB* promoter, respectively [58, 59]. Both TGF-β-sensitive reporters were induced, suggesting that SMAD3 dependent signalling is active or can be activated in HMC1.1 cells (Fig. 6B). In addition to SMAD2/(3), we attempted to detect SMAD1/SMAD5 activation at later time points (Fig. 6C,D). While SMAD2 activation was confirmed in BMMCs (Fig. 6C) and PMC-306 (Fig. 6D), we were unable to detect any activation of SMAD1/5, although SMAD1 protein was present. As a monitor of TGF-β1 treatment, TβRII was downregulated as expected (Fig. 6C,D). Consistent with the SMAD activation, we observed an upregulation of *Smad7* by TGF-β1 after 24 h in PMC-306 cells and in BMMCs (Fig. 6E,F). Furthermore, the inhibitory effect of IL-3 supplement on *Smad7* expression in the presence or absence of TGF-β1 was still observed (Fig. 6F).

In conclusion, SMAD2 is activated by TGF-β1 both early and late in an ALK5-dependent manner in all MCs. As a result of the late SMAD-response, *Smad7* is induced up to 24 h. Interestingly, we were able to demonstrate transient immediate early activation of SMAD1/5, which is absent upon stimulation of MCs for 24 h. SMAD3 is activated in the long term in HMC1.1 cells, even though *Smad3* mRNA is generally expressed at very low levels in MCs.

### Expression of Selected Marker Genes in Individual Mast Cells

We evaluated the mRNA and protein expression of specific genes that have been associated with TGF-β1 regulation in well-established MCs (Suppl. Figure 4). In RT-qPCR analysis, the “identity marker” *Kit* was found to be highly expressed (Suppl. Figure 4A, *left panel*). Conversely, *Mrgprb2* exhibited significantly lower expression in the cell lines when compared to primary cells (Suppl. Figure 4A, *right panel*).

Among the effector proteins, the “mucosal” markers *Mcpt1* and *Mcpt2*, which are expressed in both mucosal and connective tissue MCs, were found to be expressed at low levels in BMMCs and cell lines but highly in primary PMCs (Suppl. Figure 4B, *upper panel*). The “connective tissue” marker *Mcpt5* was expressed at high levels in all MCs and *Mcpt6* was mainly expressed in primary cells and nearly absent in cell lines (Suppl. Figure 4B, *middle panel*). Granzyme B (*Gzmb*), which has been found to be expressed in BMMCs [73], was significantly higher expressed in primary BMMCs (Suppl. Figure 4B, *lower panel*), [16, 51]. Hence, the mRNA levels of the analysed effector proteases (except *Mcpt5*) were generally very low in PMC-306 cells. Consistent with the mRNA expression, the proteins KIT, Tryptase and Granzyme B were highly expressed in BMMCs but either not expressed or expressed at very low levels in the cell lines. Additionally, KIT showed a different migration pattern in the MC lines compared to BMMCs (Suppl. Figure 4C, *upper panel*). Western blot analysis also confirmed the low expression of MCPT1 in L138.8A and BMMCs, and its absence in PMC-306 cells at the protein level (Suppl. Figure 4C, *lower panel*). ConA precipitation of KIT and Granzyme B confirmed their *N*-glycosylation and the lower expression of KIT and Granzyme B in cell lines [74] (Suppl. Figure 4D).

### TGF-β1/ALK5 Regulate Expression of Mucosal Mast Cell Genes

**Fig. 7:**
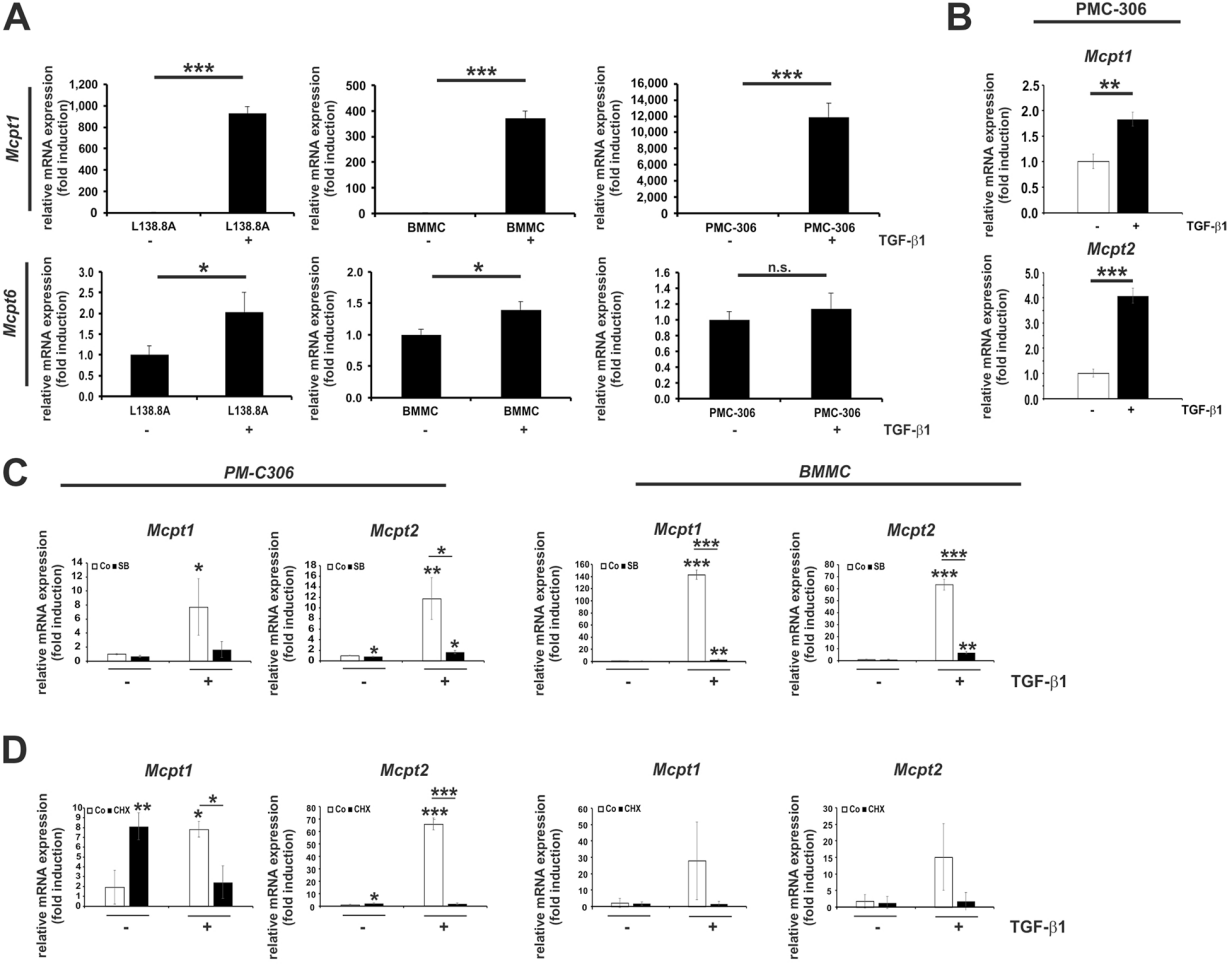
TGF-β1 induces mucosal MC specific proteases in both primary and permanent MCs. **A** To analyze *chymase* (*Mcpt1*, *mucosal/connective tissue cells, upper panel*) or tryptase *(Mcpt6, connective tissue cells, lower panel)* cells were cultured in growth medium with or without TGF-β1 (1 ng/mL) for 24 h. RNA was then isolated, reverse transcribed and RT-qPCR was performed. The expression of *Mcpt1 and Mcpt6* was normalized to *β-actin* and shown as fold induction relative to the untreated control. (*n* = 3; ± SD; * *p* < 0.05, *** p < 0.001). Note: *Mcpt1* shows high induction rates, while *Mcpt6* is induced but to a very low extent. **B** PMC-306 cells were cultured in starvation medium, stimulated with TGF-β1 (1 ng/mL) or left untreated for 90 min, followed by RNA extraction and RT-qPCR analysis of *Mcpt1* and *Mcpt2* mRNA expression. The expression of *Mcpt1 and Mcpt2* was normalized to *β-actin* and shown as fold induction relative to the untreated control. (*n* = 3; ± SD; ** p < 0.01, *** p < 0.001).** C**,**D** To demonstrate dependency on ALK5 and secondary responses, PMC-306 (*left panels*) and BMMC (*right panels*) cells were cultured in growth medium, pre-incubated with or without SB431542 (5 µM), and then stimulated in the absence or presence of TGF-β1 (1 ng/mL) for 6 h (**C**), or cultured in growth medium, pre-incubated with or without CHX (10 µg/mL), and then stimulated in the absence or presence of TGF-β1 for 6 h (**D**). RNA was isolated, reverse transcribed, and qPCR was performed to detect *Mcpt1* and *Mcpt2.* SB431542 completely inhibits TGF-β1 responses, while CHX strongly reduces *Mcpt1* and *Mcpt2* expression*.* The expression of *Mcpt1 and Mcpt2* was normalized to *β-actin* and shown as fold induction relative to the untreated control. (*n* = 3; ± SD; * *p* < 0.05, ** p < 0.01, *** p < 0.001)

Our expression analysis suggests that primary MCs exhibit a broader expression pattern compared to the analysed immortalized MC lines. To examine the responsiveness of MC marker genes to TGF-β1 in individual cell types, we stimulated cells under normal culture conditions to minimize interference with cell viability itself (Fig. 7A). The mRNA of *Mcpt1* was substantially upregulated after 24 h by TGF-β1 in L138.8A, BMMC, and PMC-306 (Fig. 7A, *upper panel*). In contrast, the expression of the *Mcpt6* gene was only slightly induced by TGF-β1 in L138.8A cells and BMMCs, but not in PMC-306 cells (Fig. 7A, *lower panel*).

Further analysis revealed that the MC marker genes *Mcpt2*, *Mcpt8*, and the integrin receptor β-subunit *Itgae*, all associated with a mucosal MC signature [75], can be upregulated by TGF-β1 in all three cell types (Suppl. Figure 5). In contrast, genes associated with the connective tissue MC type were regulated more heterogeneously. The expression of *Mcpt5* and *Mcpt6* was only slightly modulated by TGF-β1, while the expression of *Mcpt4*/*Mcpt7* was more significantly upregulated, and *Cpa3* was strongly downregulated by TGF-β1 (Suppl. Figure 5B).

The induction of *Mcpt1* and *Mcpt2* mRNA was already evident after 90 min of TGF-β1 application, consistent with previous reports showing that these target genes are directly regulated by SMADs [46, 47] (Fig. 7B). In line with the TGF-β1-regulated expression of *Smad7* (cf. Figure 5B), the TGF-β1-induced expression of *Mcpt1* and *Mcpt2* was abolished by ALK5 inactivation (Fig. 7C). However, unlike *Smad7*, the expression of *Mcpt1* and *Mcpt2* was inhibited by CHX (Fig. 7D). Therefore, the induction of *Mcpt1* and *Mcpt2* by TGF-β1 after 6 h was already indirect and relied on a secondary response that requires protein expression [70], although this effect was only a clear tendency in BMMC (Fig. 7D, * right panel*).

At the protein level, the expression of MCPT1 resembled that of a late response gene, showing induction from 24 h onwards, dependent on ALK5 in BMMCs (Suppl. Figure 6A,B). Similarly, in PMC-306 cells, the induction of MCPT1 increased within the analysed time frame starting at 14 h, also dependent on ALK5 (Suppl. Figure 6C,D). In PMC-306 and BMMC, MCPT1 is released into the medium only upon IgE mediated degranulation after long-term (24 h) pre-incubation with TGF-β1, while MCPT6 is released only by BMMC independent of TGF-β1 (Suppl. Figure 6E). These results confirm that, consistent with mRNA expression, MCPT1 is responsive to TGF-β 1and is expressed and released into the medium upon the appropriate trigger.

### TGF-β1-Mediated *Mcpt1* Expression is Negatively Regulated by IL-3/ERK1/2

**Fig. 8:**
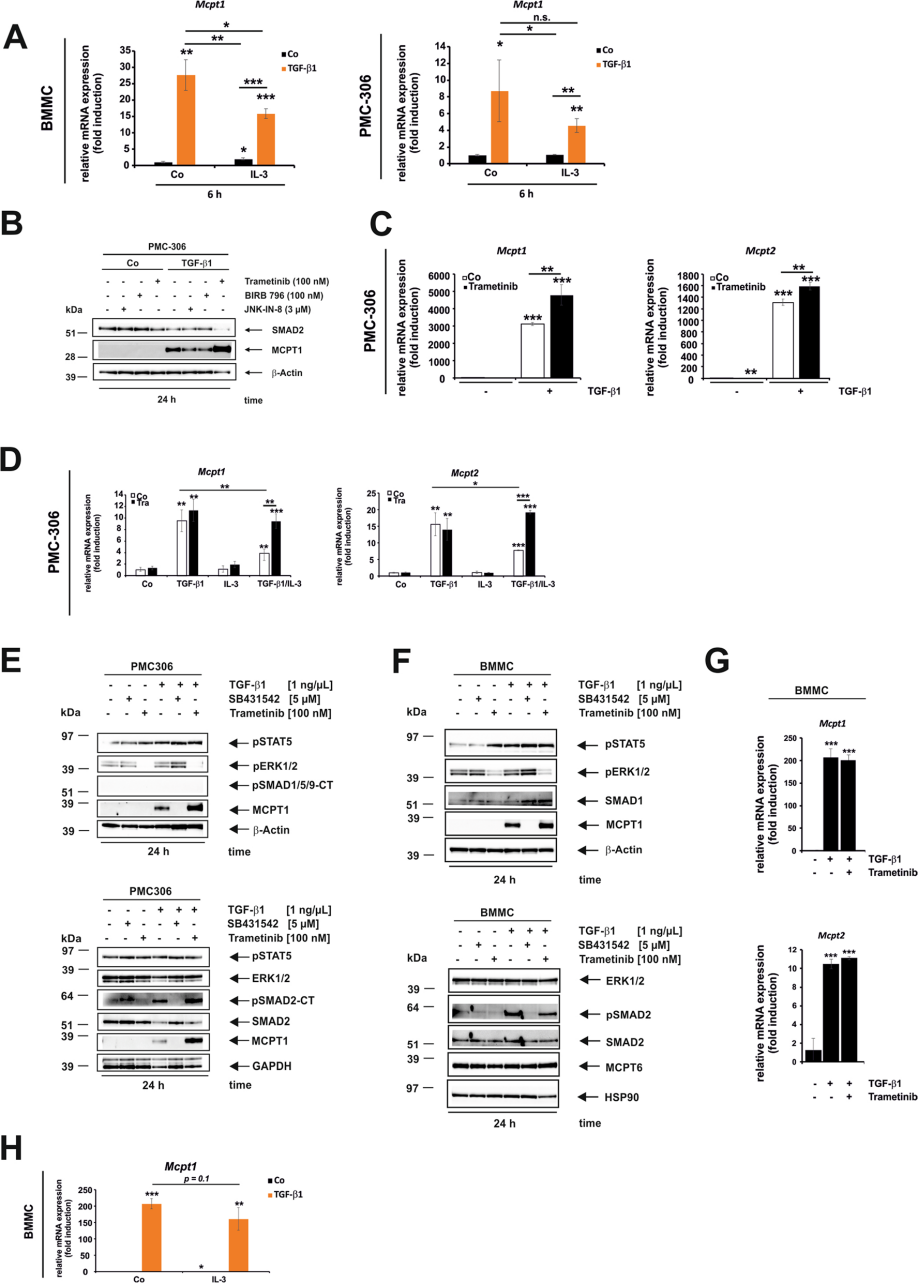
Impact of IL-3-mediated ERK1/2 activation and its inhibition on TGF-β1-mediated Mcpt1 gene and protein expression. **A** BMMC (*left panel*) and PMC-306 cells (*right panel)* were cultured in starvation medium in the absence or presence of TGF-β1 (1 ng/mL) with or without IL-3 for 6 h. Cells were harvested, RNA isolated and qPCR performed to detect *Mcpt1.* Expression of *Mcpt1* was normalized to *β-actin* and displayed as fold induction relative to the untreated control. (*n* = 3; ± SD; * *p* < 0.05, ** p < 0.01, *** p < 0.001). **B** To determine the role of MAPKs in the IL-3-mediated reduction of TGF-β1-regulated target gene expression, PMC-306 cells were cultured in growth medium, pre-incubated in the absence or presence of the indicated inhibitors for 1 h and stimulated with and without TGF-β1 for 24 h. Cellular proteins were analysed by Western blot with the indicated antibodies. Equal loading was demonstrated using a β-Actin-specific antibody. **C** PMC-306 cells were cultured in growth medium, pre-incubated in the presence or absence of Trametinib (100 nM) for 1 h and stimulated or not with TGF-β1 for 24 h. RNA was extracted and qPCR performed to detect *Mcpt1* and *Mcpt2*. Expression of *Mcpt1 and Mcpt2* was normalized to *β-actin* and displayed as fold induction relative to the untreated control. (*n* = 3; ± SD; ** p < 0.01, *** p < 0.001). **D** PMC-306 cells were cultured in starvation medium and stimulated with or without TGF-β1, IL-3 medium supplement or a combination thereof in the presence or absence of Trametinib (100 nM) for 6 h. RNA was extracted and RT-qPCR performed to detect *Mcpt1* and *Mcpt2*. Expression of *Mcpt1 and Mcpt2* was normalized to *β-actin* and displayed as fold induction relative to the untreated control. (*n* = 3; ± SD; * *p* < 0.05, ** p < 0.01, *** p < 0.001). **E**,**F** PMC-306 (**E**) or BMMC (**F**) were cultured in growth medium, pre-incubated in the presence or absence of SB431542 (5 µM) or Trametinib (100 nM) for 1 h and stimulated or not with TGF-β1 for 24 h. Cellular proteins were extracted and analysed in Western blot with the indicated specific antibodies. Equal protein loading was determined by re-probing the membranes with a β-Actin/GAPDH (**E**) or β-Actin/HSP90 (**F**) specific antibodies. **G** BMMCs were cultured in growth medium, pre-incubated with Trametinib (100 nM) for 1 h or not and stimulated or not with TGF-β1 (1 ng/mL) for 24 h. mRNA was extracted from the corresponding samples and the cDNAs were analysed for expression of *Mcpt1* and *Mcpt2* by qPCR. Expression of *Mcpt1* and *Mcpt2* was normalized to *β-actin* and displayed as fold induction relative to the untreated control. (*n* = 3; ± SD; *** p < 0.001). **H** BMMCs were cultured in starvation medium and stimulated or not with TGF-β1 (1 ng/mL), IL-3 or a combination thereof for 24 h. mRNA was extracted from the corresponding samples and the cDNAs were analysed for expression of *Mcpt1* by qPCR. Expression of *Mcpt1* was normalized to *β-actin* and displayed as fold induction relative to the untreated control. (*n* = 3; ± SD; * *p* < 0.05, ** p < 0.01, *** p < 0.001)

IL-3 reduced the expression of TGF-β1-induced *Smad6* and *Smad7* target genes (cf. Figure 5C). To determine if this also applies to TGF-β1-responsive MC markers, we examined *Mcpt1* expression under the same conditions after 6 h in BMMCs (Fig. 8A, *left panel*).

IL-3 reduced the TGF-β1-induced expression of *Mcpt1*, suggesting that it had the same inhibitory impact as seen for *Smad6* and *Smad7* (Fig. 5C). In PMC-306 cells, *Mcpt1* expression only showed a tendency with respect to IL-3-mediated repression due to high variations in the control (Fig. 8A, *right panel*). Since IL-3 strongly activated ERK1/2 (Fig. 5A, D), we determined if MAPKs are involved in the regulation of TGF-β1 responses. Therefore, PMC-306 cells were treated or not with individual MAPK inhibitors for 24 h in the presence or absence of TGF-β1 (Fig. 8B). JNK inhibition by JNK-IN-8 and p38 inhibition by BIRB 796 decreased and ERK1/2 inhibition by Trametinib increased TGF-β1-mediated MCPT1 expression (Fig. 8B). This effect was confirmed at the mRNA level by stimulation of PMC-306 cells with or without TGF-β1 for 24 h in the presence or absence of Trametinib showing that TGF-β1-mediated *Mcpt1* and *Mcpt2* expression increases upon ERK1/2 inactivation (Fig. 8C). To analyse if ERK1/2 acts downstream of IL-3, PMC-306 were stimulated with TGF-β1, IL-3 or a combination thereof in the presence or absence of Trametinib (Fig. 8D). IL-3, which by itself does not affect basal *Mcpt1* or *Mcpt2* expression, reduced the TGF-β1-mediated induction of *Mcpt1* and *Mcpt2,* and Trametinib abolished this effect (Fig. 8D). Those results suggest that IL-3 engages ERK1/2 to decrease TGF-β1 mediated *Mcpt1*/*Mcpt2* expression in PMC-306 cells. However, blocking ERK1/2 activation by Trametinib has no consistent impact on SMAD2 phosphorylation in PMC-306 (Fig. 8E). In contrast, analysis of BMMCs under the same conditions showed no enhancing effect of Trametinib on the MCPT1 protein (Fig. 8F) and mRNA (Fig. 8G) expression induced by TGF-β1 after 24 h. Additionally, the inhibitory effect of IL-3 on TGF-β1-induced *Mcpt1* mRNA expression present at 6 h was lost at 24 h, implying that the impact of IL-3/ERK1/2 on TGF-β1 signalling is under kinetic control in BMMCs (Fig. 8A,H). In conclusion, basal ERK activation represses TGFβ1-induced *Mcpt1*/*Mcpt2* expression, and this attenuating mechanism can be enforced in the presence of IL-3, indicating that the micro-environment controls TGF-β1 signalling and the MC phenotype.

## Discussion

It has been documented that MCs are recruited to inflammatory environments enriched in TGF-β1, such as the lung and liver [40, 75]. In this context, MCs may impact fibrogenic cells in different ways, including (i) activation of PAR2 by tryptase, (ii) activation of latent TGF-β1 by chymase, and (iii) secretion of TGF-β1 itself. In addition, MCs respond to and are regulated by TGF-β1, allowing for control of chemotaxis, cell cycle regulation, and differentiation. This study examines the mode(s) of action and responses of MCs related to TGF-β1 by comparing the recently developed PMC-derived cell line PMC-306 with primary BMMCs and immortalized derivatives such as L138.8A, commonly used for TGF-β1 signalling analysis [37, 70].

Since the first step in TGF-β1 signalling is the binding of the ligand to the core receptor complex (TβRI (ALK5) and TβRII), it is not surprising that both receptors are expressed at the mRNA and protein levels in all analysed murine MCs. The TβRI/TβRII ratio in MCs is high compared to HSCs, which are specialized in TGF-β signalling (Fig. 2). In contrast to the core receptors, the accessory type III receptors, especially TβRIII (Betaglycan), could not be detected in MCs. These receptors fine-tune TGF-β responses, and the absence of Betaglycan was unexpected given its widespread expression [76]. The second type III receptor, Endoglin, is expressed at protein level near the detection limit, with low mRNA levels found in primary BMMCs and PMCs.

In endothelial cells, it has been shown that TGF-β1, in addition to SMAD2/SMAD3, mediates the activation of the “BMP”-SMADs, SMAD1, and SMAD5, through a “lateral” signalling pathway that is initiated by the type I receptor ALK1 [66]. This signalling pathway is promoted by Endoglin. The functional coupling of these two receptors is evident, because mutations in both genes can cause hereditary hemorrhagic telangiectasia [77]. However, there is currently no data available for the activation of SMAD1/SMAD5 in MCs. qPCR analysis of cDNAs from different MCs did not result in amplification of *Alk1* transcripts, although its expression could be confirmed in primary HSCs and permanent HSC lines (Fig. 2) [67]. Therefore, the receptors that mediate the activation of the TGF-β1/SMAD1/SMAD5 pathway in endothelial cells, *i.e.* ALK1 and Endoglin, appear to not be expressed in MCs. Nevertheless, TGF-β1 stimulation of BMMCs and PMC-306 cells showed a strong immediate early activation of SMAD2 and SMAD1/SMAD5 (Fig. 4).

Meanwhile, it has been shown that activation of SMAD1/SMAD5 by TGF-β1 is not restricted to i) endothelial cells and ii) not only mediated by ALK1 [78], but also by other receptor complexes, including ALK2 and ALK3 [79–81]. The expression of these receptors in MCs is currently being analysed. *Alk2* and *Alk3* mRNAs have already been detected in MCs (data not shown). However, it is unclear whether the corresponding receptor proteins are expressed because the MCs (PMC-306/BMMCs) did not respond to different BMPs (BMP2/BMP4/BMP7) that are known to bind to those receptors [82] (Fig. 4A). The small molecule inhibitor Dorsomorphin, which blocks the BMP type I receptor (ALK1, ALK2, ALK3, ALK6) [83], could not be used due to cross reactions with other kinases (Suppl. Figure 2A-C). However, the analysis shown here clearly proves that ALK5 is involved in all TGF-β1 responses, as demonstrated by the use of the selective TGF-β type I receptor small molecule inhibitor SB431542 [79, 80, 84, 85].

The consecutive nuclear translocation and induction of the target gene *Id1* confirm the activation of the SMAD1/5 pathway (Fig. 4F). Additionally, we have demonstrated the expression of the target gene and feedback inhibitor *Smad6* (Fig. 4F). In comparison to SMAD2, the activation of SMAD1/5 is delayed, remaining inactive after 5 min, while SMAD2 is already activated (Fig. 5A,B). However, the phosphorylation of SMAD1/5 is only transient, present at 6 h but absent at 24 h, contrasting with to the persistent activation of SMAD2. These differences in kinetics have been observed in C2C12 myoblast cells, where there is a delayed and concentration-dependent activation of SMAD1 by TGF-β1 compared to SMAD2/3 [80]. The timed shutdown of SMAD1/5-mediated ID1 expression after 3 h, as a result of SMAD3-induced ATF3 expression, has been demonstrated in epithelial cells [86]. However, it is unknown if a similar cross talk between SMAD2/3 and SMAD1/5 signaling is active in MCs, but the fact that CHX increases *Id1* and *Smad6* mRNA expression points in this direction (Fig. 5E).

An unexpected discovery regarding SMAD3 is its low mRNA expression in MCs compared to HSCs (Suppl. Figure 2). Additionally, we were unable to detect SMAD3 or phospho-SMAD3, except for the activation of the SMAD3-specific reporter genes CAGA-Luc and SBE-Luc after 24 h in HMC1.1 cells (Fig. 6). The question of whether the induction of the target gene and feedback inhibitor *Smad7* necessarily indicates SMAD3 activation is uncertain, as its expression has been shown to be regulated by both SMAD2 and SMAD3 [87]. However it has been demonstrated that in BMMCs TGF-β directly activates transcription of *Mcpt7 *via SMAD3, rather than through SMAD2 [37]. Nevertheless, the significance of SMAD3 as the key regulator of these processes is questionable, as the experiments were conducted with promoter reporters of *Mcpt1*/*Mcpt7* and analysed in non-MC systems like HepG2, which are deficient in MC-specific regulatory factors. Moreover, analysis in BMMCs shows that *Mcpt1* and *Mcpt7* are induced by TGF-β1 in WT and *Smad3* knock-out cells, arguing against an exclusive contribution of SMAD3 [70]. The individual roles of SMAD2 and SMAD3 have been emphasised in BMMCs using siRNA knock down of each *Smad*, with knock down of *Smad2* completely abolishing the expression of *Mcpt1* and *Mcpt2*, while knockdown of *Smad3* only partially affected their expression [47]. These results indicate that SMAD2 plays a more important role in regulating the aforementioned MC effectors. However the exact roles of the individual SMADs in regulating the expression of MC target genes need to be addressed in more detail in the future.

Regarding the role of MAPKs in TGF-β1 signalling, previous studies have discussed the involvement of individual enzymes in the transcriptional regulation of *Mcpt1* and *Mcpt7* [70]. However, when examining the induction of MAPKs by IL-3 (Fig. 5), it becomes challenging to determine if TGF-β1 itself activates MAPKs, specifically ERK1/2, to regulate protease expression in the presence of IL-3 under steady-state conditions. One significant obstacle in analysing long-term responses in MCs, particularly in the presence of TGF-β1, is the loss of vitality due to the deprivation of survival factors and the pro-apoptotic effect of TGF-β1 itself (Suppl. Figure 3) [88, 89]. Therefore, long-term TGF-β1 responses in PMC-306 were only conducted under steady-state conditions (in the presence of IL-3 and SCF). However, in those long-term conditions we could not consistently demonstrate a negative impact of TGF-β1 on ERK1/2 activation (Fig. 8E), but we did observe a strong induction of MCPT1. Conversely, short-term responses up to 8 h revealed that TGF-β1 reduced ERK1/2 activation starting from 90 min on (Fig. 5A). This suggests that ERK1/2 does not need to be turned off to allow for TGF-β1-mediated MCPT1 expression, but rather a reduction in ERK1/2 activity by Trametinib clearly enhances TGF-β1-mediated MCPT1 expression (Fig. 8E). These findings highlight a negative influence of the IL-3/ERK1/2 axis on TGF-β1-mediated MCPT1 expression. It has long been recognized that IL-3 induces an immature phenotype in BMMC by promoting early differentiation genes like *Mcpt6* and BMMC proliferation while inhibiting late differentiation genes like *Mcpt1* and *Mcpt2* [90]. Here, we present data suggesting that an IL-3/ERK1/2 axis triggers a secondary response that blocks TGF-β1 signalling to prevent late gene responses and differentiation.

The overall importance of IL-3 in MC propagation in vitro has been shown [91], and this importance may also be relevant in the liver in vivo*.* Activated T cells are the main source of IL-3, and these cells infiltrate the liver in the context of PSC, as demonstrated by human patient samples and mouse models, potentially supplying IL-3 for MC expansion [92, 93]. Whether targeting IL-3 could serve as a basis for therapeutic intervention is currently unknown, but research has been conducted on SCF and MC-derived TGF-β1 in corresponding mouse models [24, 40].

A comparison of TGF-β1-mediated gene expressions of “classical targets”, namely *Smad7* and *Smad6*, and “MC targets”, specifically *Mcpt1* and *Mcpt2*, was conducted to determine common and cell-type-specific effects of IL-3. The results showed a downregulation of *Smad7* and *Smad6* in PMC-306 and in BMMCs after 6 h, as well as a decrease in *Mcpt1* and *Mcpt2* expression after 6 h in the presence of IL-3 (Fig. 5, Fig. 8). This aligns with the fact that IL-3 promotes proliferation over the anti-proliferative and pro-differentiation actions of TGF-β1. However, it is worth noting that the signalling mechanisms engaged by TGF-β1 for “classical” targets and “MC” specific targets differ. This is evident when using the translation inhibitor CHX (Fig. 5, Fig. 7). While CHX promotes the mRNA expression of *c-jun*, *Smad6* and *Smad7*, it reduces the expression of *Mcpt1* and *Mcpt2*. Therefore, TGF-β1 signaling directly regulates *c-jun, Smad6* and *Smad7,* because no protein synthesis is needed*.* In contrast, TGF-β1-mediated gene expression of *Mcpt1* and *Mcpt2* is indirect and blocked by CHX, thus requiring a secondary response. In this context, the relevance of persistent activation of SMAD2 for the prolonged expression of *Mcpt1* and *Mcpt2* is at least questionable.

A similar indirect regulation of *Mcpt1* by TGF-β1 has been demonstrated in BMMCs [70]. Based on the presented results, it can be concluded that the direct TGF-β1 response is affected in steady state conditions (via IL-3 signaling). However, this effect is unlikely to be mediated by a reduction in SMAD2 phosphorylation or nuclear translocation, as these factors remained unchanged in response to IL-3 after 30 min and thereafter (Fig. 5, Fig. 8). In BMMCs, it has been shown that inhibition of p38 reduces the expression of *Mcpt1* mRNA, while inhibition of ERK1/2 increases its expression [70]. In PMC-306 cells, we found that inhibition of p38 reduces TGF-β1 mediated MCPT1 protein expression, and inhibition of ERK1/2 leads to an increase in expression after 24 h (Fig. 8). Furthermore, we demonstrated that inhibition of ERK1/2 by Trametinib eliminates the negative effect of IL-3 on TGF-β1-mediated *Mcpt1* and *Mcpt2* mRNA expression after 6 h (Fig. 8). Although the negative impact of IL-3 is still observed in BMMCs when analysing *Smad7* after 24 h, neither the mRNA level nor the protein expression of MCPT1 is affected by IL-3 at 24 h. This suggests differences in the regulation of *Mcpt1* by TGF-β1 in BMMCs compared to PMC-306 (Fig. 8), and thus goes hand in hand with the diverse MC phenotypes dependent on micro-environmental conditions in various tissues. However, when speculating about different responses of BMMC and PMC-306, it has to be taken into account that PMC-306 are cultured in the presence (dependency) of SCF and BMMCs are not. SCF is a strong inducer of ERK1/2 activation and is currently being analysed for a role in the TGF-β1-mediated induction of MCPT1 and MCPT2. With a focus on proliferation, SCF was much more potent in counteracting the anti-proliferative effect of TGF-β1 compared to IL-3 (Supplemental Fig. 3). On the other hand, SCF is able to induce late connective tissue genes, e.g. *Mcpt4,* similar to TGF-β1 [94].

In summary, we have demonstrated that TGF-β1 induces activation of both the SMAD2 and SMAD1/5 signaling pathways with distinct kinetics. Additionally, TGF-β1 promotes the expression of several genes associated with the mucosal MC gene signature. Under steady-state conditions, TGF-β1 responses, such as *Smad6/Smad7* and *Mcpt1/Mcpt2*, are reduced in the presence of IL-3. Conversely, we have shown that blocking ERK1/2 activation increases TGF-β1-mediated *Mcpt1/Mcpt2* expression. Therefore, we hypothesize that ERK1/2 acts as a mutual switch between IL-3-driven proliferation and TGF-β1-promoted mucosal MC differentiation.

## Supplementary Information

Supplementary Material 1.

Supplementary Material 2.

Supplementary Material 3.

Supplementary Material 4.

Supplementary Material 5.

Supplementary Material 6.

Supplementary Material 7.

Supplementary Material 8.

Supplementary Material 9.

## Data Availability

The data is provided in the manuscript or supplementary information files. Additional data is available from the corresponding authors on reasonable request.

## Abbreviations

BMMC(s): bone marrow-derived mast cell(s)
CHX: cycloheximide
CTMC(s): connective tissue mast cell(s)
HSC(s): hepatic stellate cell(s)
MC(s): mast cell(s)
MMC(s): mucosal mast cell(s)
PMC(s): peritoneum-derived mast cell(s)
SCF: stem cell factor
WT: wild-type
IL-3: interleukin-3
