# Supplementary material for: TGF-β1 Induces Mucosal Mast Cell Genes and is Negatively Regulated by the IL-3/ERK1/2 Axis
Source: Cell Commun Signal. 2025 Feb 11;23:76. doi: 10.1186/s12964-025-02048-8 (PMC11817834; doi:10.1186/s12964-025-02048-8)
Supplement: Supplementary file 1 — Supplementary Material 1. [file 12964_2025_2048_MOESM1_ESM.docx]

# Suppl. Table 1: Special reagents used in this study

| **Reagent** | **Source (order number)** | **Stock solution / Remarks** |
| --- | --- | --- |
| Mouse TGF-β1 | R&D Systems (#7666-MB) | 10 ng/mL, 4 mM HCl/ 0.1% BSA, recombinant mouse TGF-β1 |
| BMP-2 | R&D Systems (#355-BM) | 50 ng/mL, 4 mM HCl/ 0.1% BSA, recombinant human/mouse/rat BMP-2 protein |
| PDGF-BB | R&D Systems (#220-BB) | 50 ng/mL, 4 mM HCl, recombinant human PDGF-BB |
| SB431542 | Sigma-Aldrich (#S4317) | 10 mM in DMSO, selective TGF-β receptor kinase inhibitor for ALK4, ALK5, and ALK7 |
| PD98059 | Sigma-Aldrich (#P215) | 10 mM in DMSO, potent MEK1/2 inhibitor |
| Trametinib (GSK1120212) | Selleckchem (#S2673) | 10 mM in DMSO, potent MEK1/2 inhibitor |
| Doramapimod (BIRB 796) | Abcam (#ab142166) | 20 mM in DMSO, selective cell permeable p38 MAPK inhibitor |
| JNK-IN-8 | Sigma-Aldrich (#SML1246) | 20 mM in DMSO, irreversible JNK inhibitor |
| Ruxolitinib | Sigma-Aldrich (HY-50856) | 10 mM in DMSO, Jak1/2 inhibitor |
| Dorsomorphin (Compound C) | Sigma-Aldrich (#P5499) | 10 mM in DMSO, BMP type I receptor inhibitor |
| Concanavalin A beads | Calbiochem (#234568) | Con A, *Canavalia ensiformis*, agarose conjugate |
| Cycloheximide | Sigma-Aldrich (#239763-M) | 10 mg/mL in DMSO |
| Anti-Dinitrophenyl antibody, mouse monoclonal (IgE isotype, clone SPE-7) | Sigma-Aldrich (#D8406) | ~1 mg/mL, buffered aqueous solution |
| Albumin Dinitrophenyl-HSA | Sigma-Aldrich (#A6661) | 40 mg/mL in H_2_O |
| Lipofectamine^TM^ 2000 | Invitrogen (#11668-019) | Ready to use transfection reagent |
| TransIT-LT1 | Mirus (#MIR 2304) | Ready to use transfection reagent |
| Opti-MEM | Invitrogen (# 31985062) | Reduced serum medium |
| DMSO (cell culture grade) | AppliChem (#A3672) | liquid |
| Trypan blue | Sigma-Aldrich (#T8154) | Premade 0.4% liquid, sterile-filtered solution |
| cOomplete™ protease inhibitor cocktail | Roche (#11836145001) | Provided as tablets |
| Phosphatase inhibitor cocktail 2 | Sigma-Aldrich (#P5726) | Aqueous solution |
| Bovine serum albumin | Sigma-Aldrich (#A9418) | Lyophilized powder, suitable for cell culture |
