# Supplementary material for: TGF-β1 Induces Mucosal Mast Cell Genes and is Negatively Regulated by the IL-3/ERK1/2 Axis
Source: Cell Commun Signal. 2025 Feb 11;23:76. doi: 10.1186/s12964-025-02048-8 (PMC11817834; doi:10.1186/s12964-025-02048-8)
Supplement: Supplementary file 2 — Supplementary Material 2. [file 12964_2025_2048_MOESM2_ESM.docx]

# Suppl. Table 2: Primers used in this study

| Gene | Ref. Seq. | Forward | | Reverse |
| --- | --- | --- | --- | --- |
| Loading | | | | |
| *Actb* | NM_007393 | 5’-CTCTAGACTTCGAGCAGGAGATGG-3’ | 5’-ATGCCACAGGATTCCATACCCAAGA-3’ | |
| Mast cell related | | | | |
| *Kit* | NM_021099.3 | 5’-GATCTGCTCTGCGTCCTGTT-3’ | 5’-CTTGCAGATGGCTGAGACG-3’ | |
| *Mrgprb2* | NM_175531.4 | 5’-CCTCAGCCTGGAAAACGAAC-3’ | 5’-CCATCCCAACCAGGGAAATGA-3’ | |
| *Mcpt1* | NM_008570.1 | 5’-ACGGACAGAGGTTCTGAGGA-3’ | 5’-GAGCTCCAAGGGTGACAGTG-3’ | |
| *Mcpt2* | NM_008571.2 | 5’-TTCACCACTAAGAACGGTTCG-3’ | 5’-CTCCAAGGATGACACTGATTTCA-3’ | |
| *Itgae* | NM_008399.4 | 5’-GCAGAGAACCACAGGACGAA-3’ | 5’-TGCTGCTGCCAATGATGAGA-3’ | |
| *Mcpt4* | NM_010779.2 | 5’-GCACTTCTCTTGCCTTCTGG-3’ | 5’-ATGTAAGGGCGAGAATGTGG-3’ | |
| *Mcpt5 (Cma1)* | NM_010780.3 | 5’-ATCTGCTGCTCCTTCTCCTG-3’ | 5’-ACTCCGTGCCTCCAATGA-3’ | |
| *Mcpt6 (Tpsb2)* | NM_010781.3 | 5’-TGCTGTGTGCTGGAAATACC-3’ | 5’-CCCTTCACTTTGCAGACCA-3’ | |
| *Mcpt7 (Tpsab1)* | NM_031187.4 | 5’-TCCTCACTGTGTCCAAATGC-3’ | 5’-CCTTCTCGTGTCATAGCTGGA-3’ | |
| *Mcpt8* | NM_008572.1 | 5’-GGATGTTCCTGCTCCTGGT-3’ | 5’-TGGGGTTTGGACTCTGTACC-3’ | |
| *Cpa3* | NM_007753.2 | 5’-GCTATTAATTCCTTATGGCTACACATT-3’ | 5’-GTGGCAATCCTTGCAACTTT-3’ | |
| *Gzmb* | NM_013542.3 | 5’-GCTGCTCACTGTGAAGGAAGT-3’ | 5’-TGGGGAATGCATTTTACCAT-3’ | |
| TGF-β related | | | | |
| *Tbri (Alk5)* | NM_009370.4 | 5’-GCAGCTCCTCATCGTGTTG-3’ | 5’-AGAGGTGGCAGAAACACTGTAAT-3’ | |
| *Alk1 (Acvrl1)* | NM_009612.3 | 5’-CGAGTCGCCCTGTCCAAAG-3’ | 5’- GGTCCTTGGAGATAGGAGAAGAG-3’ | |
| *Tbrii* | NM_009371.3 | 5’-AGAAGCCGCATGAAGTCTG-3’ | 5’-GGCAAACCGTCTCCAGAGTA-3’ | |
| *Tbriii* | NM_011578.4 | 5’-CTCCCCAGGGGTCTTCTC-3’ | 5’-CTTGGTCAGCCTTAGTGACAGA-3’ | |
| *Endoglin* | NM_007932.2 | 5’-CATTGCACTTGGCCTACGA-3’ | 5’-GATGTTGACTCTTGGCTGTCC-3’ | |
| *Smad1* | NM_008539.4 | 5’-CTACTGGCGCAGTCTGTGAA-3’ | 5’- GATCTCAATCCAGCAGGGGG-3’ | |
| *Smad2* | NM_010754.5 | 5’-AGGACGGTTAGATGAGCTTGAG-3’ | 5’-GTCCCCAAATTTCAGAGCAA-3’ | |
| *Smad3* | NM_016769.4 | 5’-CGCAGGTTCTCCAAACCTCT-3’ | 5’-AATGTCTCCCCAACTCGCTG-3’ | |
| *Smad5* | NM_008541.3 | 5’-AATAAAGTTGCGGCGCGTG-3’ | 5’-CTTGACAGGTGCCATAGGCT-3’ | |
| *Smad7* | NM_001042660.1 | 5’-ACCCCCATCACCTTAGTCG-3’ | 5’-GAAAATCCATTGGGTATCTGGA-3’ | |
| *Smad6* | NM_008542.3 | 5’-GTTGCAACCCCTACCACTTC-3’ | 5’-GGAGGAGACAGCCGAGAATA-3’ | |
| *Id1* | NM_010495.3 | 5’-GCGAGATCAGTGCCTTGG-3’ | 5’-CTCCTGAAGGGCTGGAGTC-3’ | |
| c*-jun* | NM_010591.2 | 5’-ACATGCTCAGGGAACAGGTG-3’ | 5’-CTGCGTTAGCATGAGTTGGC-3’ | |
| Trafficking related | | | | |
| *Caveolin1* | NM_007616.5 | 5’-AACGACGACGTGGTCAAGA-3’ | 5’-TGTCACAGTGAAGGTGGTGAA-3’ | |
| *Caveolin2* | NM_016900.4 | 5’-CCTCACCAGCTCAACTCTCA-3’ | 5’-CACATATTTGCTGATTTCAAAGAGA-3’ | |
