# Supplementary material for: TGF-β1 Induces Mucosal Mast Cell Genes and is Negatively Regulated by the IL-3/ERK1/2 Axis
Source: Cell Commun Signal. 2025 Feb 11;23:76. doi: 10.1186/s12964-025-02048-8 (PMC11817834; doi:10.1186/s12964-025-02048-8)
Supplement: Supplementary file 3 — Supplementary Material 3. [file 12964_2025_2048_MOESM3_ESM.docx]

# Suppl. Table 3: Details of antibodies used in this study

| Protein | Antibody | Source | Clonality | Epitope | Specificity | Dilution |
| --- | --- | --- | --- | --- | --- | --- |
| Loading controls | | | | | | |
| β-actin (AC-15) | A5441 | Sigma | Monoclonal (mouse) | Slightly modified β-cytoplasmic actin N-terminal peptide (Ac-Asp-Asp-Asp-Ile-Ala-Ala-Leu-Val-Ile-Asp-Asn-Gly-Ser-Gly-Lys) conjugated to keyhole limpet hemocyanin (KLH) | m, r, h | 1:10,000 |
| GAPDH (6C5) | sc-32233 | Santa Cruz | Monoclonal (mouse) | Purified GAPDH from rabbit muscle | m, r, h | 1:1,000 |
| Mast cell related | | | | | | |
| CD117, c-kit | A450229-2 | Agilent | Polyclonal (rabbit) | Peptide corresponding to amino acids 963 to 976 at the cytoplasmic C-terminal part of human c-kit | h | NA* |
| KIT (C-19) | sc-168 | Santa Cruz | Polyclonal (rabbit) | Peptide mapping within the C-terminus of c-kit of human origin | m, r, h | 1:500 |
| KIT (M14) | sc-1494 | Santa Cruz | Polyclonal (goat) | Peptide mapping at the C-terminus of c-kit of mouse origin | m, r, h | 1:500 |
| GZMB | CS-4275 | Cell Signaling | Polyclonal (rabbit) | Synthetic peptide corresponding to a central region of Granzyme B | m, r, h | 1:1,000 |
| Mast cell Tryptase (FL-275) | sc-32889 | Santa Cruz | Polyclonal (rabbit) | Amino acids 1-275 of full length mast cell tryptase of human origin | m, r, h | 1:1,000 |
| pSTAT5 (C11C5) | CS-9359 | Cell Signaling | Monoclonal (rabbit) | Synthetic peptide corresponding to residues surrounding Tyr694 of Stat5a | m, r, h | 1:1,000 |
| pSTAT3 (D3A7) | CS-9145 | Cell Signaling | Monoclonal (rabbit) | Synthetic phosphopeptide corresponding to residues surrounding Tyr705 of mouse Stat3 | m, r, h | 1:1,000 |
| MCPT1 (Chymase) | MAB5146 | R&D Systems | Monoclonal (rat) | Mouse myeloma cell line (NSO)-derived recombinant mouse Mcpt1 Ile21-Lys246 | m | 1:500 |
| pERK1/2 | CS-9101 | Cell Signaling | Polyclonal (rabbit) | Synthetic phospho-peptide corresponding to residues surrounding Thr202/Tyr204 of human p44 MAP kinase | m, r, h | 1:1,000 |
| ERK1/2 | CS-9102 | Cell Signaling | Polyclonal (rabbit) | Synthetic peptide derived from a sequence in the C-terminus of rat p44 MAP kinase | m, r, h | 1:1,000 |
| TGF-β1 related | | | | | | |
| TβRI (ALK5) | sc-398 | Santa Cruz | Polyclonal (rabbit) | Peptide mapping within the cytoplasmic domain of human TGFβRI | m, r, h | 1:500 |
| TβRII | sc-400 | Santa Cruz | Polyclonal (rabbit) | Peptide mapping within the cytoplasmic domain of human TGFβRII | h, m, r | 1:500 |
| TβRIII (β-glycan) | AF242PB | R&D Systems | Polyclonal (goat) | Mouse myeloma cell line (NSO)-derived recombinant human TGF-βRIII (Gly21-Asp781) | h | 1:1,000 |
| Endoglin (CD105) | AF1320 | R&D Systems | Polyclonal (goat) | Mouse myeloma cell line (NSO)-derived recombinant mouse Endoglin/CD105 (Glu27-Gly581) | m | 1:1,000 |
| SMAD2 | CS-3103 | Cell Signaling | Monoclonal (mouse) | Recombinant protein specific to the amino terminus of human Smad2 protein | m, r, h | 1:1,000 |
| SMAD2 | CS-3102 | Cell Signaling | Polyclonal (rabbit) | Synthetic peptide corresponding to residues in the amino-terminal region of Smad2/3 | m, r, h | 1:1,000 |
| pSMAD2 (CT) | CS-8828 | Cell Signaling | Monoclonal (rabbit) | Synthetic peptide corresponding to residues surrounding Ser465/467 of human Smad2 | m, r, h | 1:1,000 |
| pSMAD2 (CT) | CS-3101 | Cell Signaling | Polyclonal (rabbit) | Synthetic phospho-peptide corresponding to residues surrounding Ser465/467 of human Smad2 | m, r, h | 1:1,000 |
| SMAD1 | CS9743 | Cell Signaling | Polyclonal (rabbit) | Synthetic peptide corresponding to residues surrounding Ser190 of human Smad1 | m, h | 1:1,000 |
| pSMAD1/5/9 | CS-13820 | Cell Signaling | Monoclonal (rabbit) | Synthetic phospho-peptide corresponding to residues surrounding Ser463/465 of human Smad1 and Smad5 protein | m, r, h | 1:1,000 |
| pSMAD1/5 | CS-9516 | Cell Signaling | Monoclonal (rabbit) | Synthetic phospho-peptide corresponding to residues surrounding Ser463/465 of human Smad1 and Smad5 protein | m, r, h | 1:1,000 |
| Fibronection | AB1954 | Millipore | Polyclonal (rabbit) | Purified rat plasma fibronectin | m, r | 1:2,000 |
| COLIA1 | NB600-408 | Novus Biologicals | Polyclonal (rabbit) | Collagen Type I from human and bovine placenta | m, r, h | 1:1,000 |
| CTGF | sc-14939 | Santa Cruz | Polyclonal (goat) | Peptide mapping within an internal region of CTGF of human origin | m, r, h | 1:1,000 |
| α-SMA | CBL171 | Cymbus Biotechnol. | Monoclonal (mouse) | Synthetic decapeptide located at the N-terminus of α-smooth muscle actin | m, r, h | 1:1,000 |
| c-JUN | CS-9165 | Cell Signaling | Monoclonal (rabbit) | GST-c-Jun protein corresponding to the amino-terminal sequence of human c-Jun |  | 1:1,000 |
| GFP | sc-8334 | Santa Cruz | Polyclonal (rabbit) | Amino acids 1-238 representing full length GFP of *Aequorea victoria* origin | Tag protein | 1:1,000 |
| Trafficking | | | | | | |
| ALIX (1A12) | sc-53540 | Santa Cruz | Monoclonal (mouse) | Full length Alix of human origin | m, r, h | 1:1,000 |
| CD81 (B-11) | sc-166029 | Santa Cruz | Monoclonal (mouse) | Amino acids 90-210 of CD81 of human origin | m, r, h | 1:1,000 |
| Caveolin-1 | CS-3238 | Cell Signaling | Polyclonal (rabbit) | Synthetic peptide corresponding to residues surrounding Glu20 of human caveolin-1 | m, r, h | 1:1,000 |
| Histone H3 | CS-4499 | Cell Signaling | Monoclonal (rabbit) | Synthetic peptide corresponding to the C-terminus of human histone H3 | m, r, h | 1:1,000 |

# * This antibody was used in a Flex-kit Agilent/Dako autostainer.
