# Supplementary figures and images for: TGF-β1 Induces Mucosal Mast Cell Genes and is Negatively Regulated by the IL-3/ERK1/2 Axis
Source: Cell Commun Signal. 2025 Feb 11;23:76. doi: 10.1186/s12964-025-02048-8 (PMC11817834; doi:10.1186/s12964-025-02048-8)

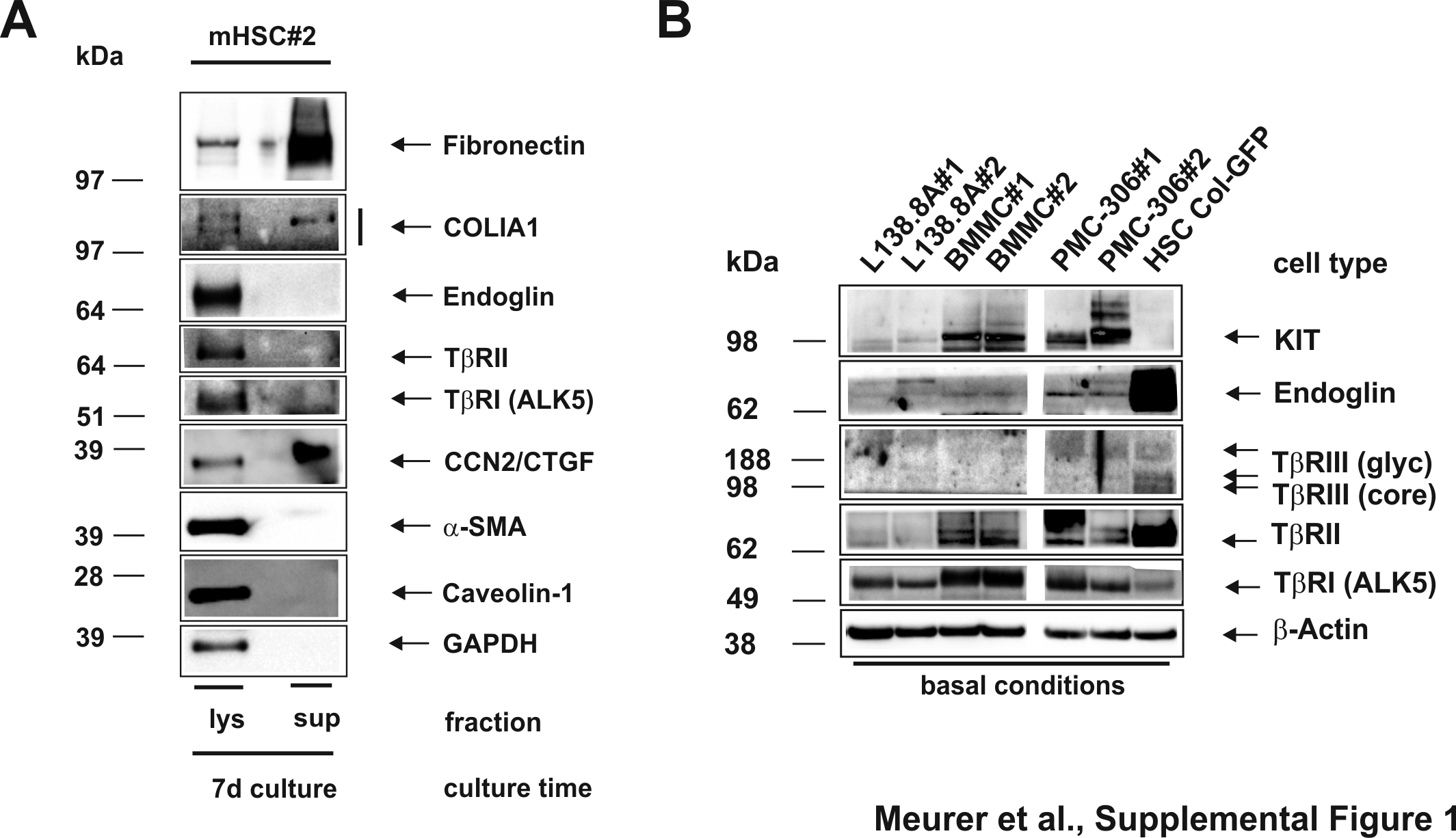

Supplement: Supplementary file 4 — Supplementary Material 4. [file 12964_2025_2048_MOESM4_ESM.jpg]

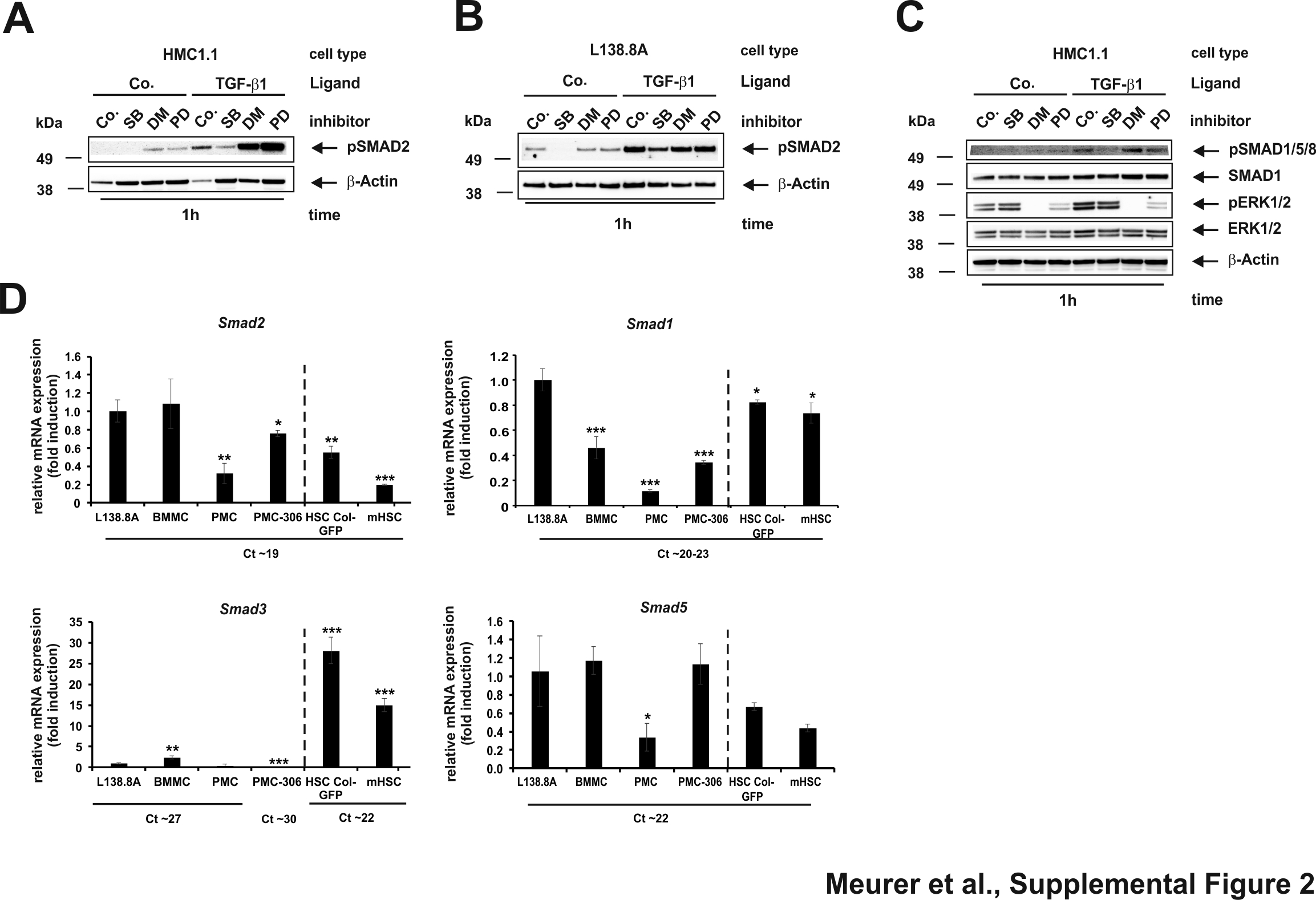

Supplement: Supplementary file 5 — Supplementary Material 5. [file 12964_2025_2048_MOESM5_ESM.jpg]

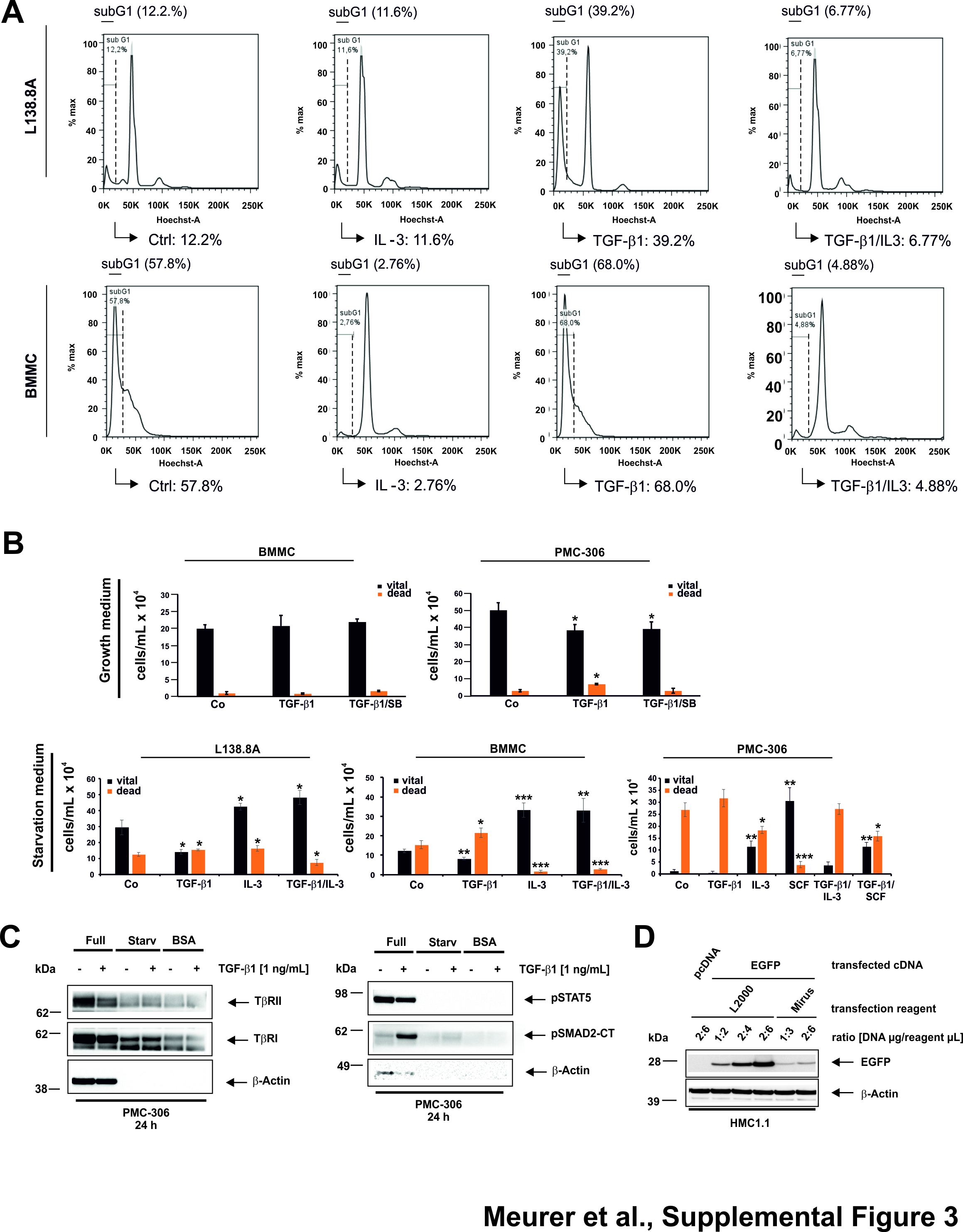

Supplement: Supplementary file 6 — Supplementary Material 6. [file 12964_2025_2048_MOESM6_ESM.jpg]

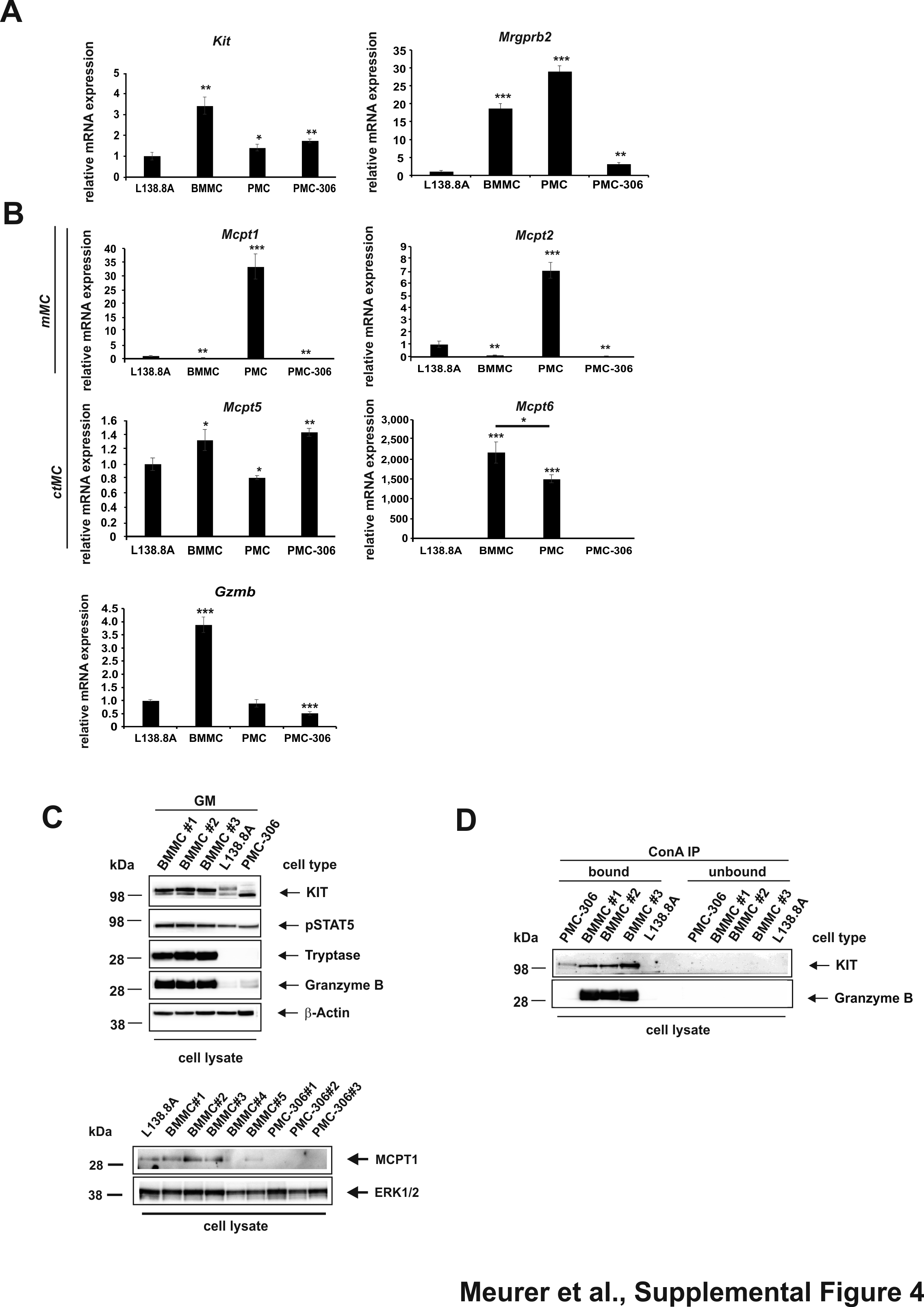

Supplement: Supplementary file 7 — Supplementary Material 7. [file 12964_2025_2048_MOESM7_ESM.jpg]

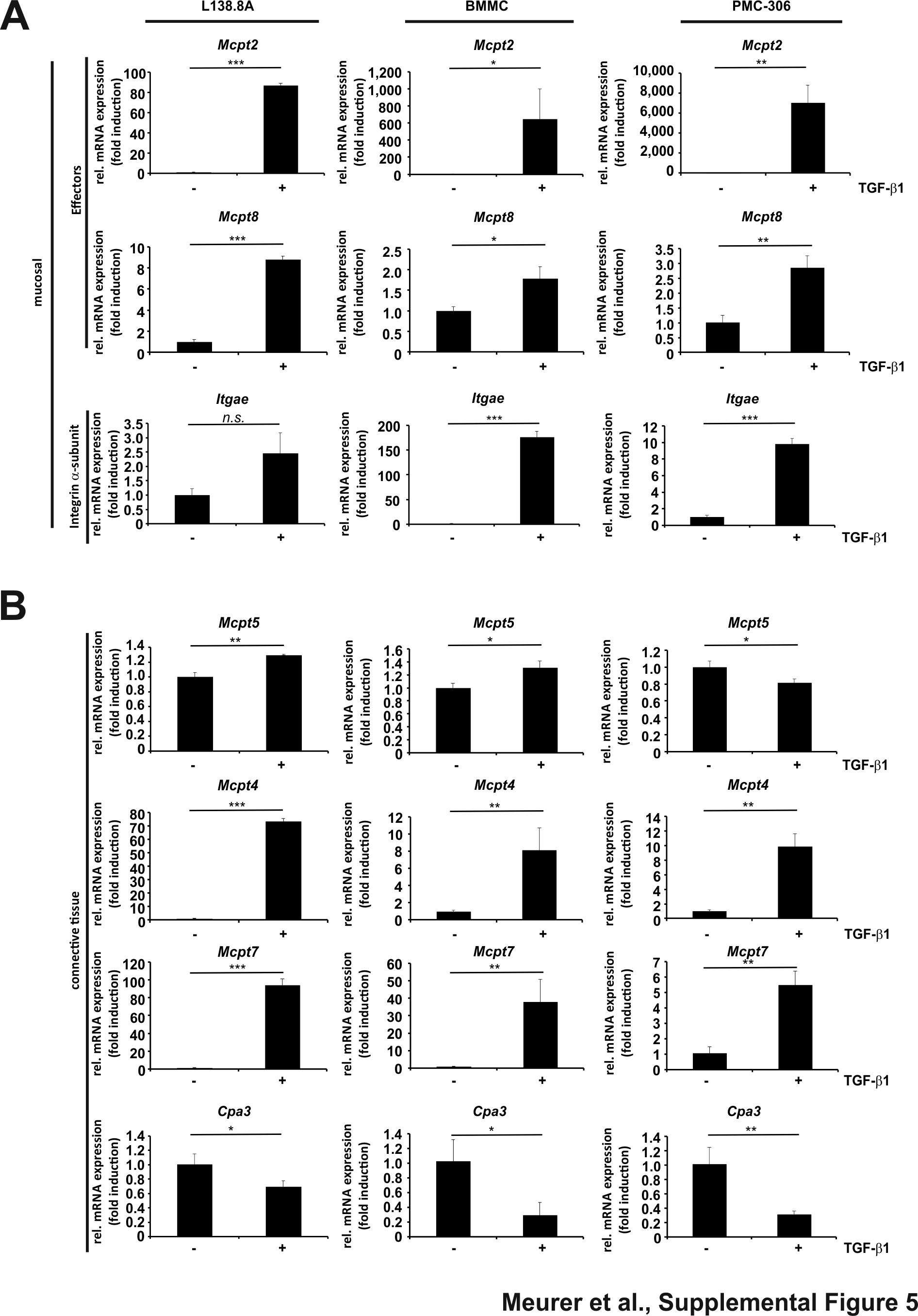

Supplement: Supplementary file 8 — Supplementary Material 8. [file 12964_2025_2048_MOESM8_ESM.jpg]
