# Supplementary material for: TGF-β1 Induces Mucosal Mast Cell Genes and is Negatively Regulated by the IL-3/ERK1/2 Axis
Source: Cell Commun Signal. 2025 Feb 11;23:76. doi: 10.1186/s12964-025-02048-8 (PMC11817834; doi:10.1186/s12964-025-02048-8)

# Supplementary File: Original Western blot results to the paper

# TGF-β1 Induces Mucosal Mast Cell Genes and is Negatively Regulated by the IL-3/ERK1/2 Axis

**Steffen K Meurer[1], Gina Bronneberg[2], Christian Penners[3], Marlies Kauffmann[2], Till Braunschweig[4], Christian Liedtke[3], Michael Huber[2], and Ralf Weiskirchen[1]**

This PDF-file contains the original Western blot results associated with the original submission

1 Fig. 2C/Suppl. Fig. 4C
2 Fig. 2D/Suppl. Fig. 4D
3 Fig. 2E/Suppl. Fig. 4C lower
4 Fig. 3A BMMC
5 Fig. 3A HSC
6 Fig. 3A L138.8A
7 Fig. 3A PMC-306
8 Fig. 3C
9 Fig. 4A
10 Fig. 4B
11 Fig. 4C
12 Fig. 5A_upper
13 Fig. 5A_2 lower
14 Fig. 5D_upper
15 Fig. 5D_lower
16 Fig. 6A
17 Fig. 6C
18 Fig. 6D
19 Fig. 7E
20 Fig. 7F
21 Fig. 7G
22 Fig. 7H
23 Fig. 8B
24 Fig. 8E
25 Suppl. Fig. 1A
26 Suppl. Fig. 1B
27 Suppl. Fig. 2A 2C
28 Suppl. Fig. 2B
29 Suppl. Fig. 2E
30 Suppl. Fig. 3C
31 Suppl. Fig. 3D

Figure 2C

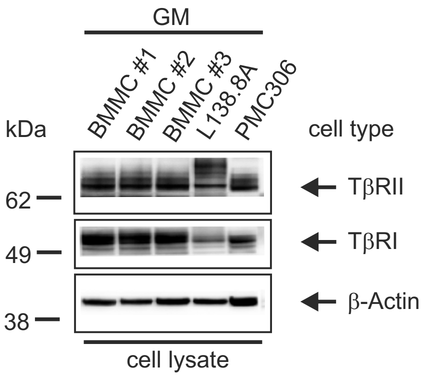


Suppl. Figure 4C

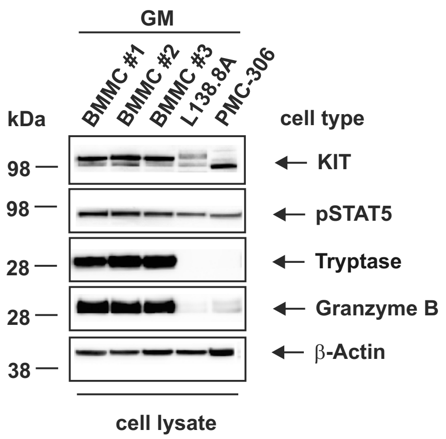


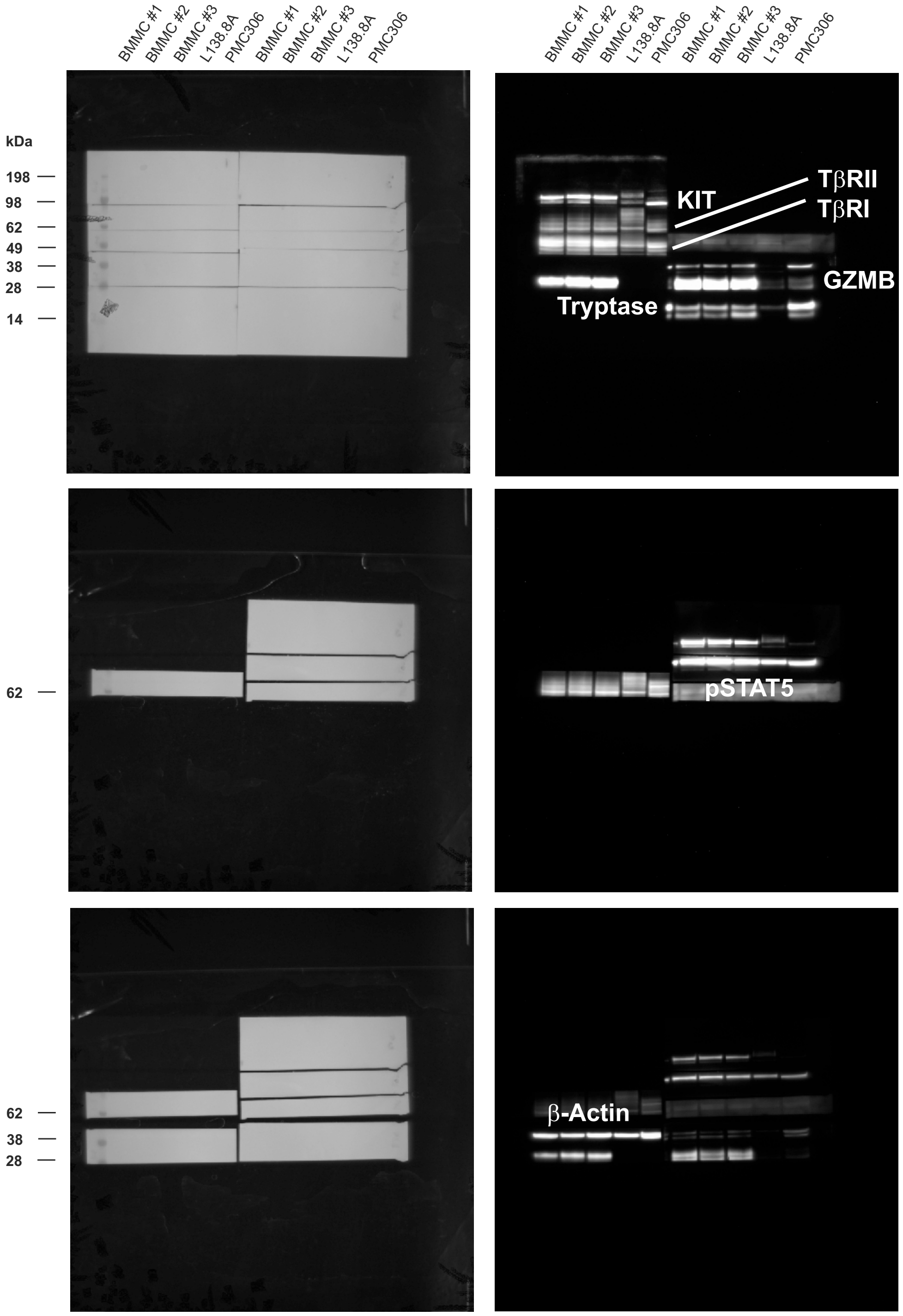

Figure 2D

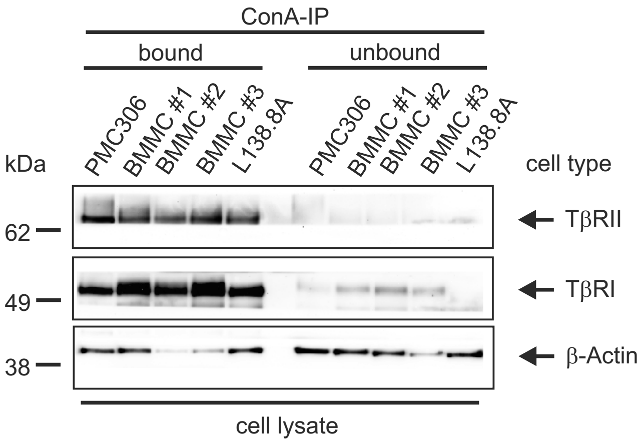


Suppl. Figure 4D

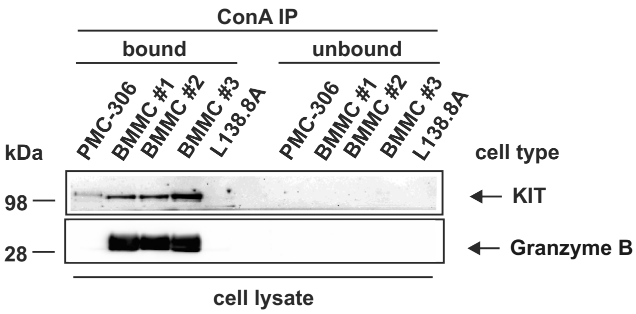


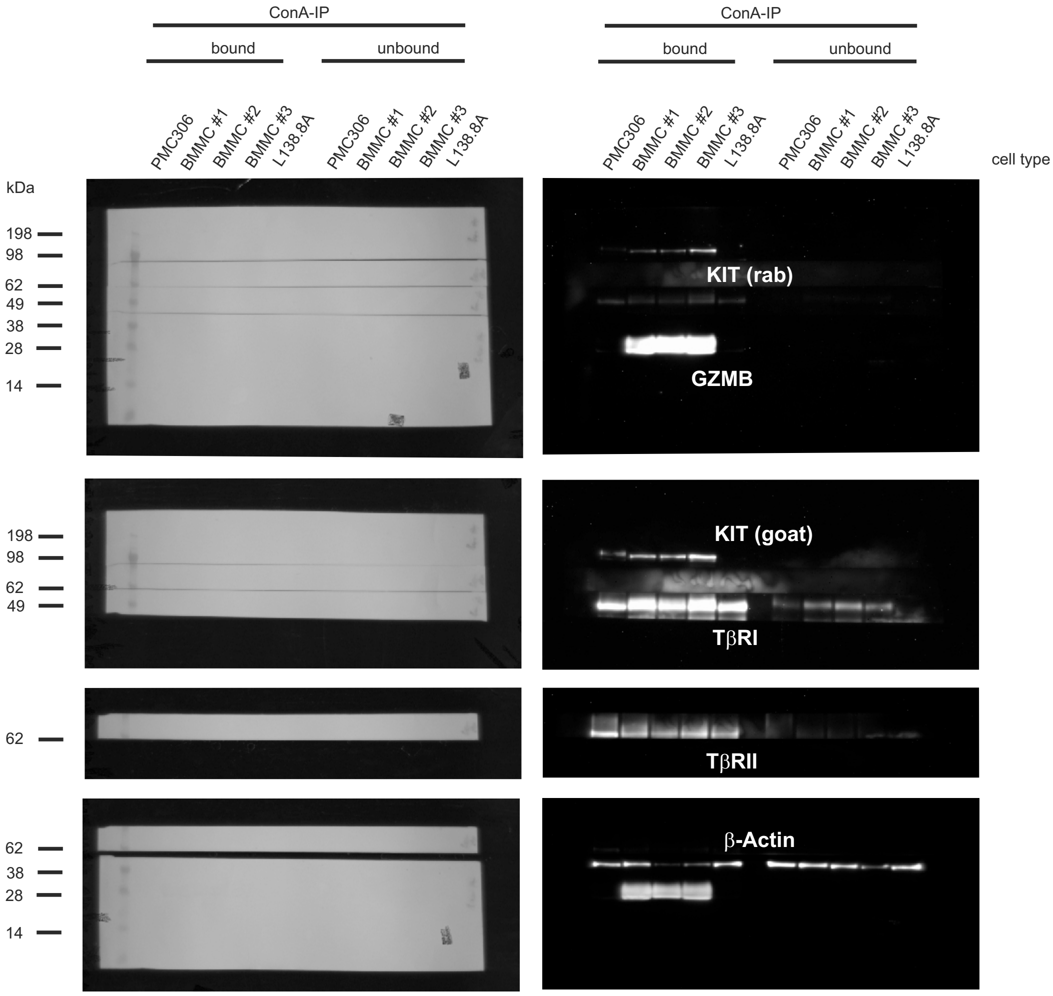

Figure 2E

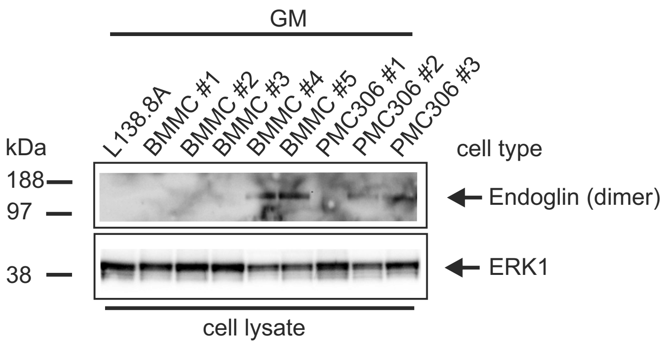


Suppl. Figure 4C

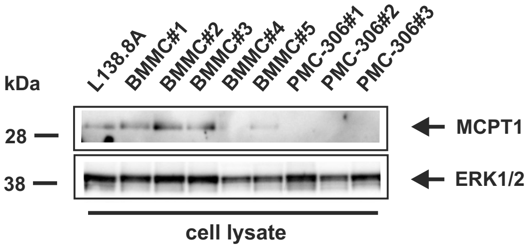


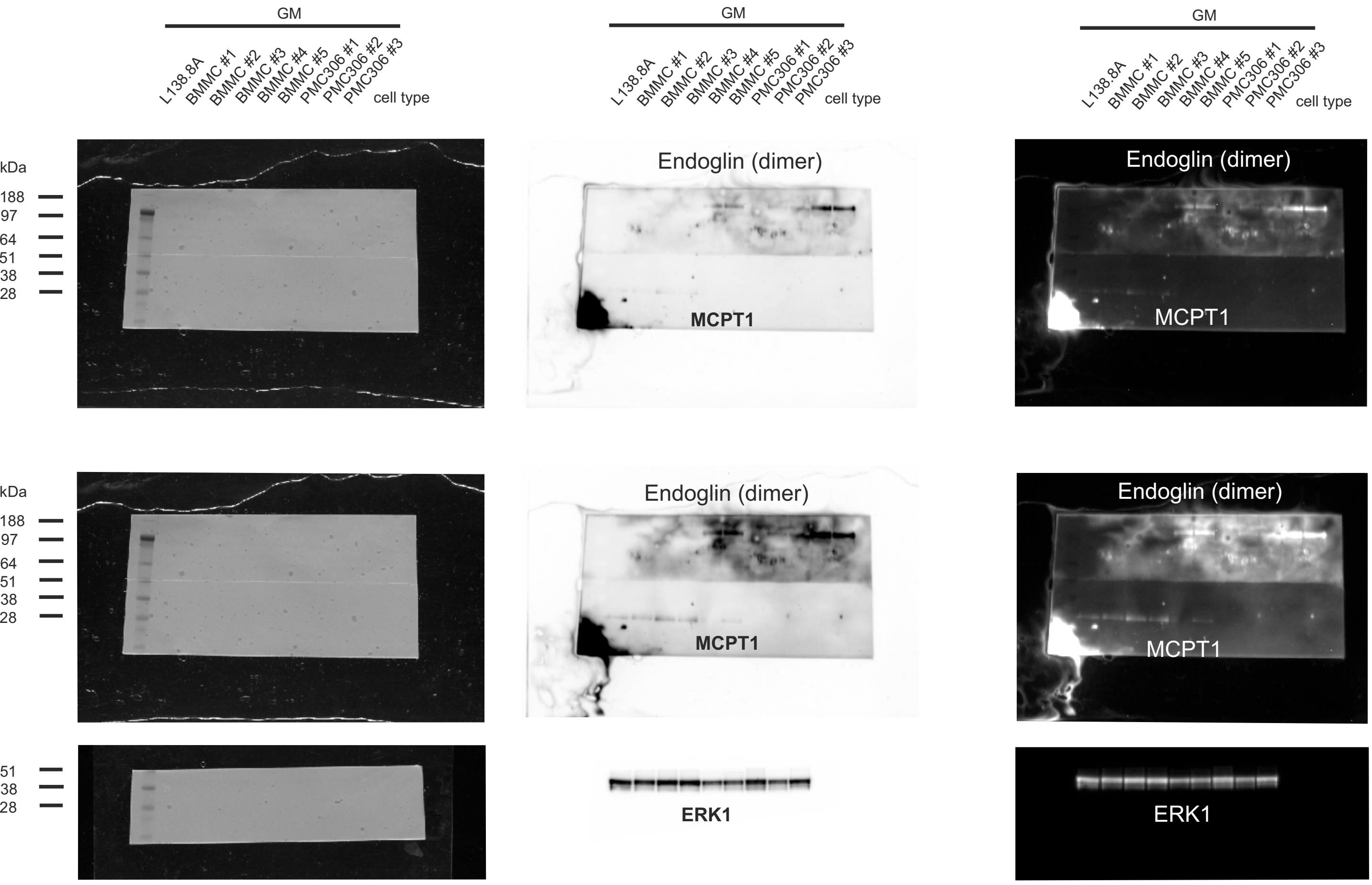

Figure 3A

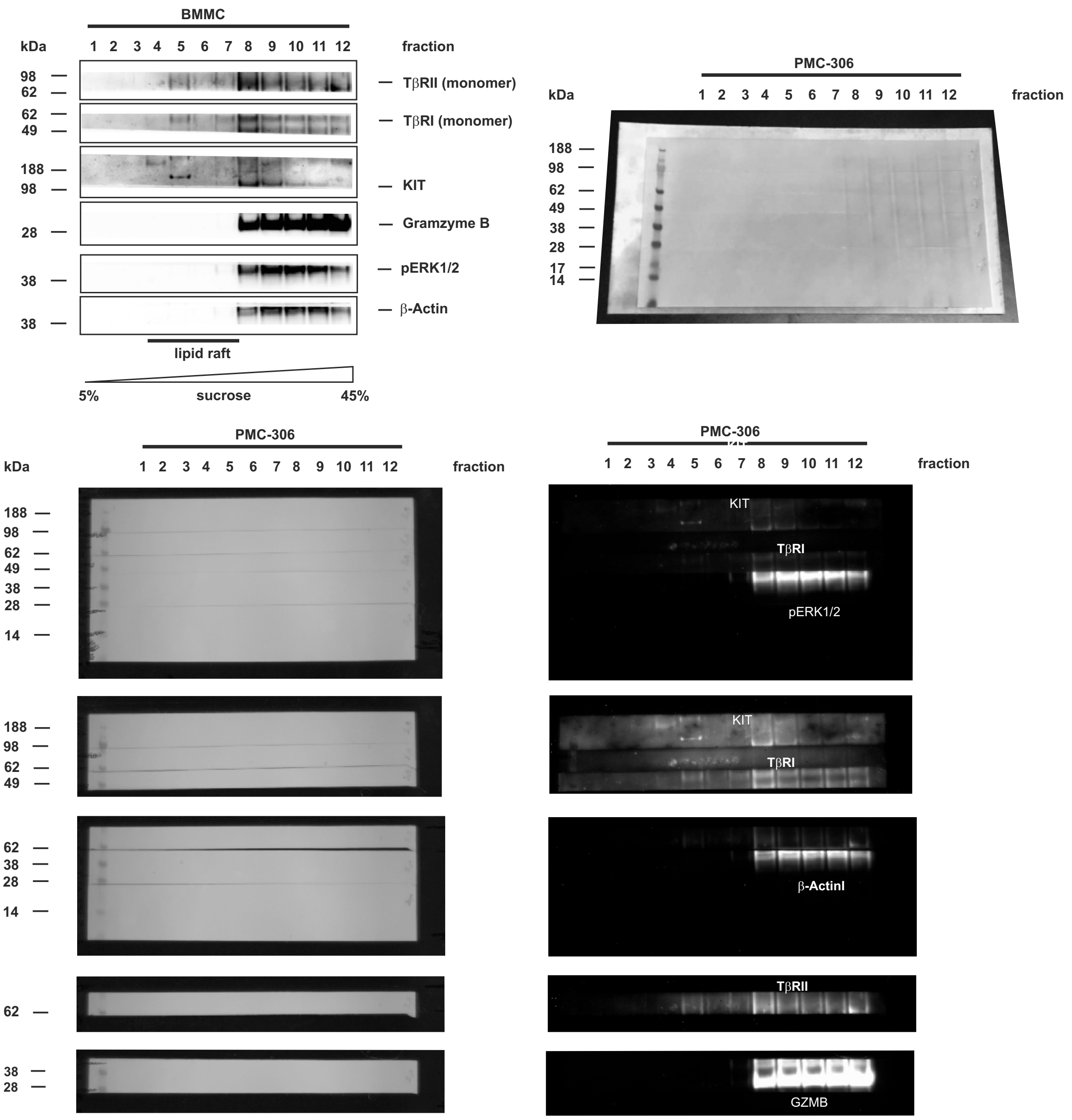
BMMC
kDa
1 2 3 4 5 6 7 8 9 10 11 12
fraction
98
62
TβRII (monomer)
62
49
TβRI (monomer)
188
98
KIT
28
Gramzyme B
38
pERK1/2
38
β-Actin
lipid raft
5%
sucrose
45%
PMC-306
kDa
1 2 3 4 5 6 7 8 9 10 11 12
fraction
188
98
62
49
38
28
17
14
PMC-306
kDa
1 2 3 4 5 6 7 8 9 10 11 12
fraction
188
98
62
49
38
28
14
188
98
62
49
62
38
28
14
62
38
28
PMC-306
1 2 3 4 5 6 7 8 9 10 11 12
fraction
KIT
TβRI
pERK1/2
KIT
TβRI
β-Actinl
TβRII
GZMB

# Figure 3A

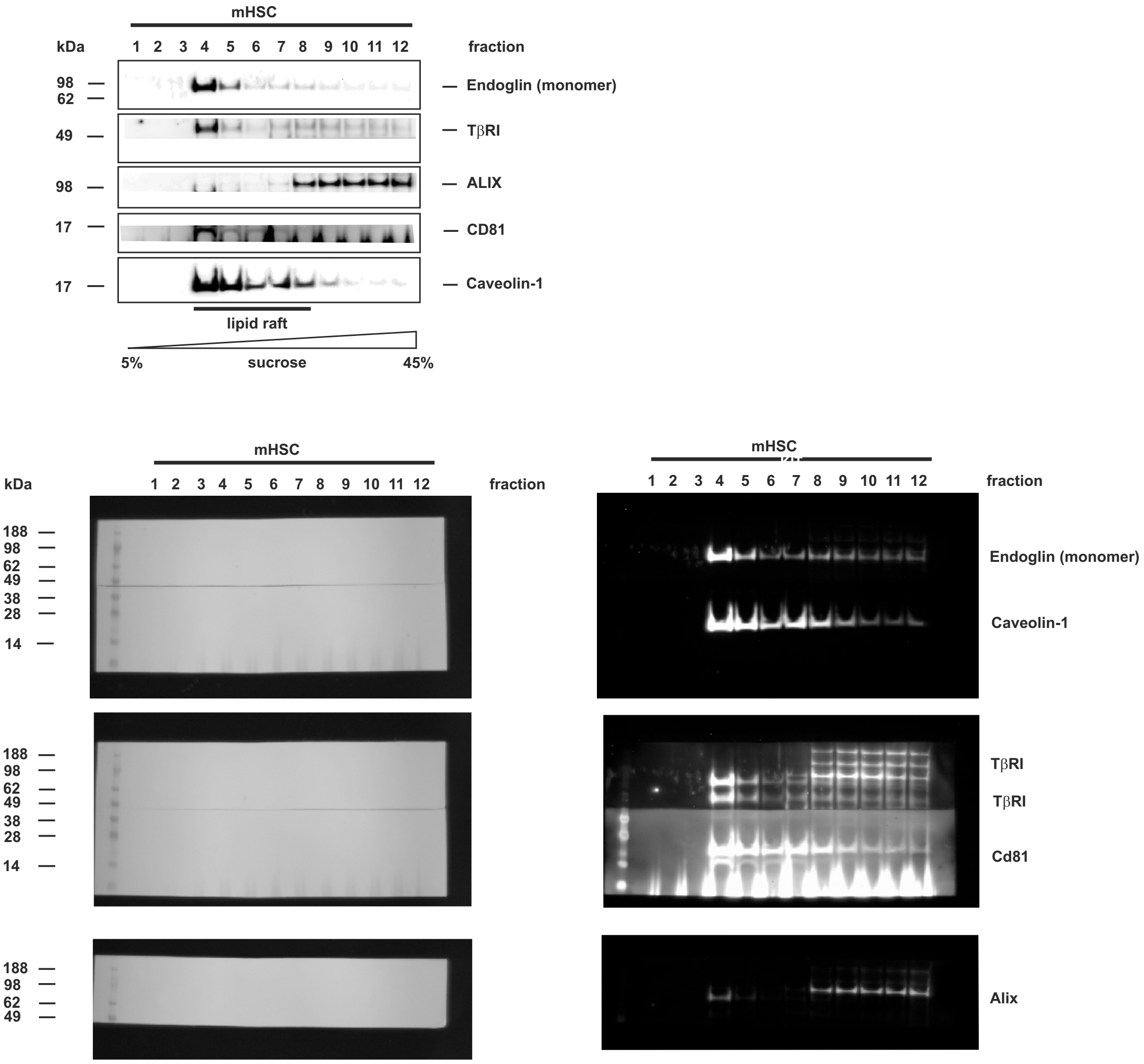
mHSC
kDa
1 2 3 4 5 6 7 8 9 10 11 12
fraction
98
62
Endoglin (monomer)
49
TβRI
98
ALIX
17
CD81
17
Caveolin-1
lipid raft
5%
sucrose
45%
mHSC
kDa
1 2 3 4 5 6 7 8 9 10 11 12
fraction
188
98
62
49
38
28
14
Endoglin (monomer)
Caveolin-1
TβRI
TβRI
Cd81
Alix

# Figure 3A

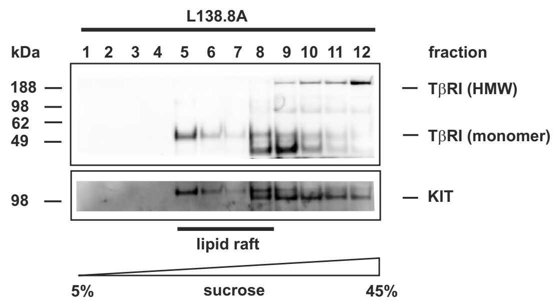
L138.8A
kDa
1 2 3 4 5 6 7 8 9 10 11 12
fraction
188
98
62
49
TβRI (HMW)
TβRI (monomer)
98
KIT
lipid raft
5%
sucrose
45%

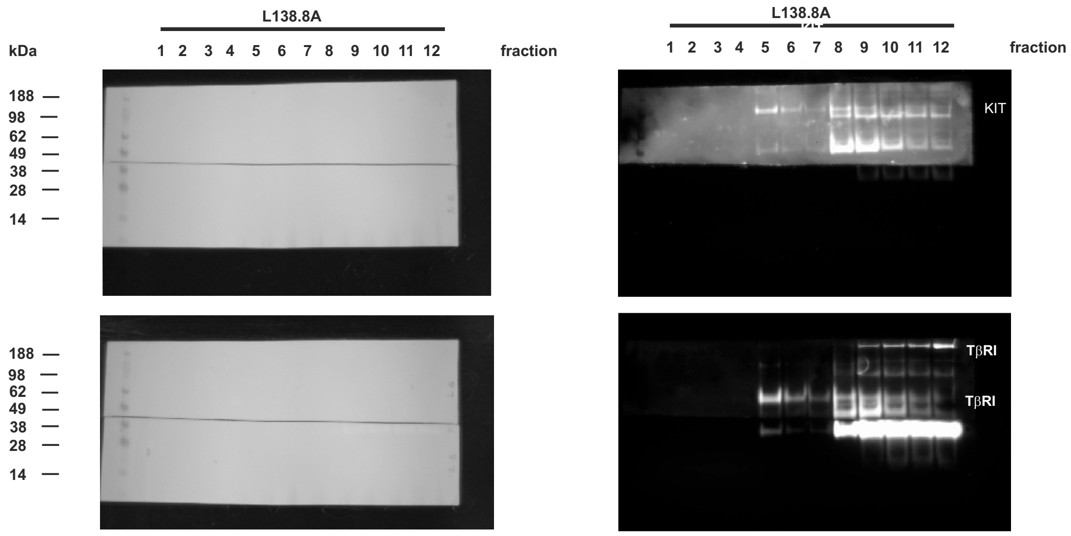
L138.8A
kDa
1 2 3 4 5 6 7 8 9 10 11 12
fraction
188
98
62
49
38
28
14
L138.8A
1 2 3 4 5 6 7 8 9 10 11 12
fraction
KIT
188
98
62
49
38
28
14
TβRI
TβRI

Figure 3A

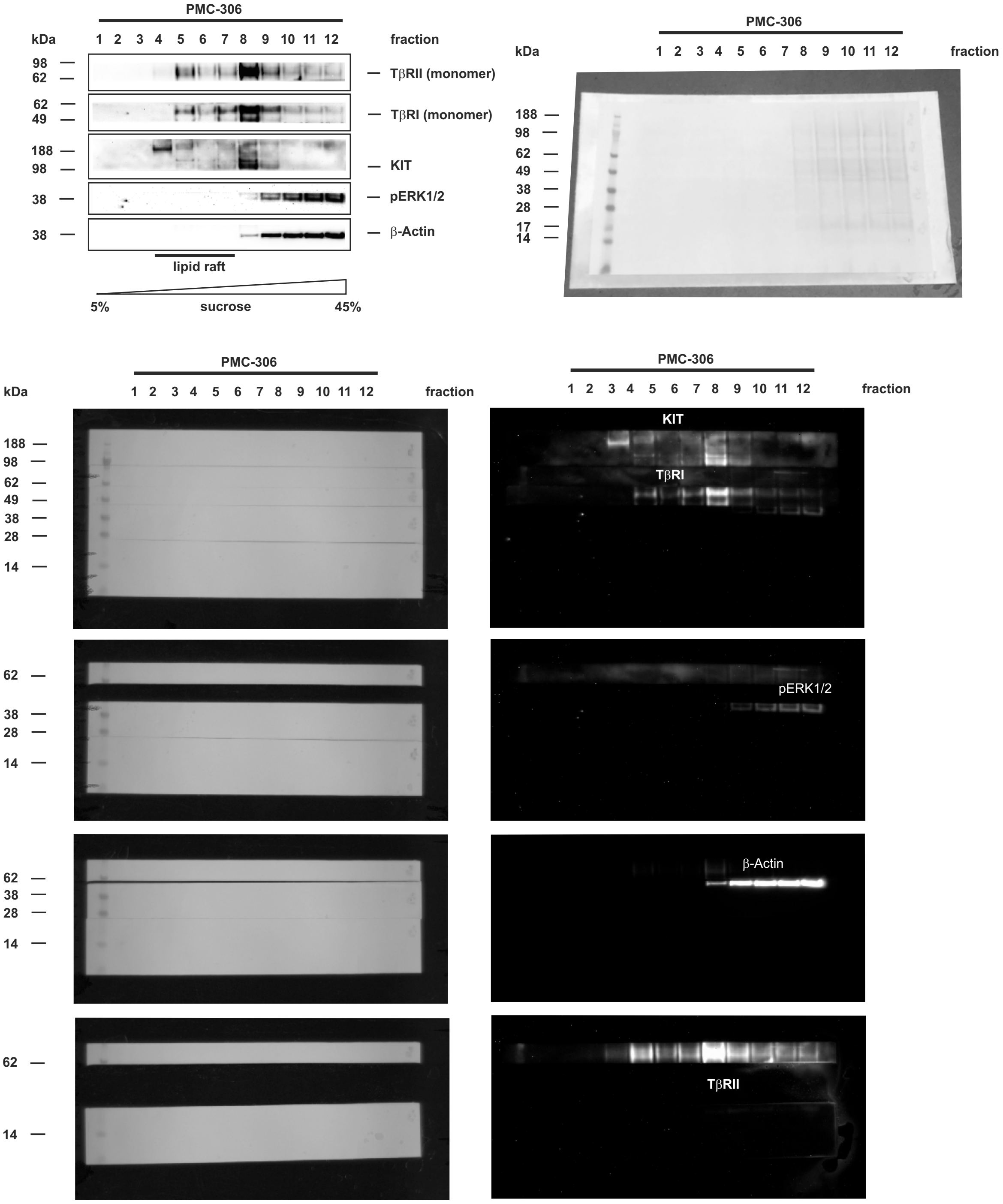

PMC-306
kDa
1 2 3 4 5 6 7 8 9 10 11 12
fraction
98
62
TβRII (monomer)
62
49
TβRI (monomer)
188
98
KIT
38
pERK1/2
38
β-Actin
lipid raft
5%
sucrose
45%
PMC-306
kDa
1 2 3 4 5 6 7 8 9 10 11 12
fraction
188
98
62
49
38
28
17
14
PMC-306
kDa
1 2 3 4 5 6 7 8 9 10 11 12
fraction
188
98
62
49
38
28
14
PMC-306
1 2 3 4 5 6 7 8 9 10 11 12
fraction
KIT
TβRI
62
38
28
14
pERK1/2
62
38
28
14
β-Actin
62
14
TβRII

Figure 3C

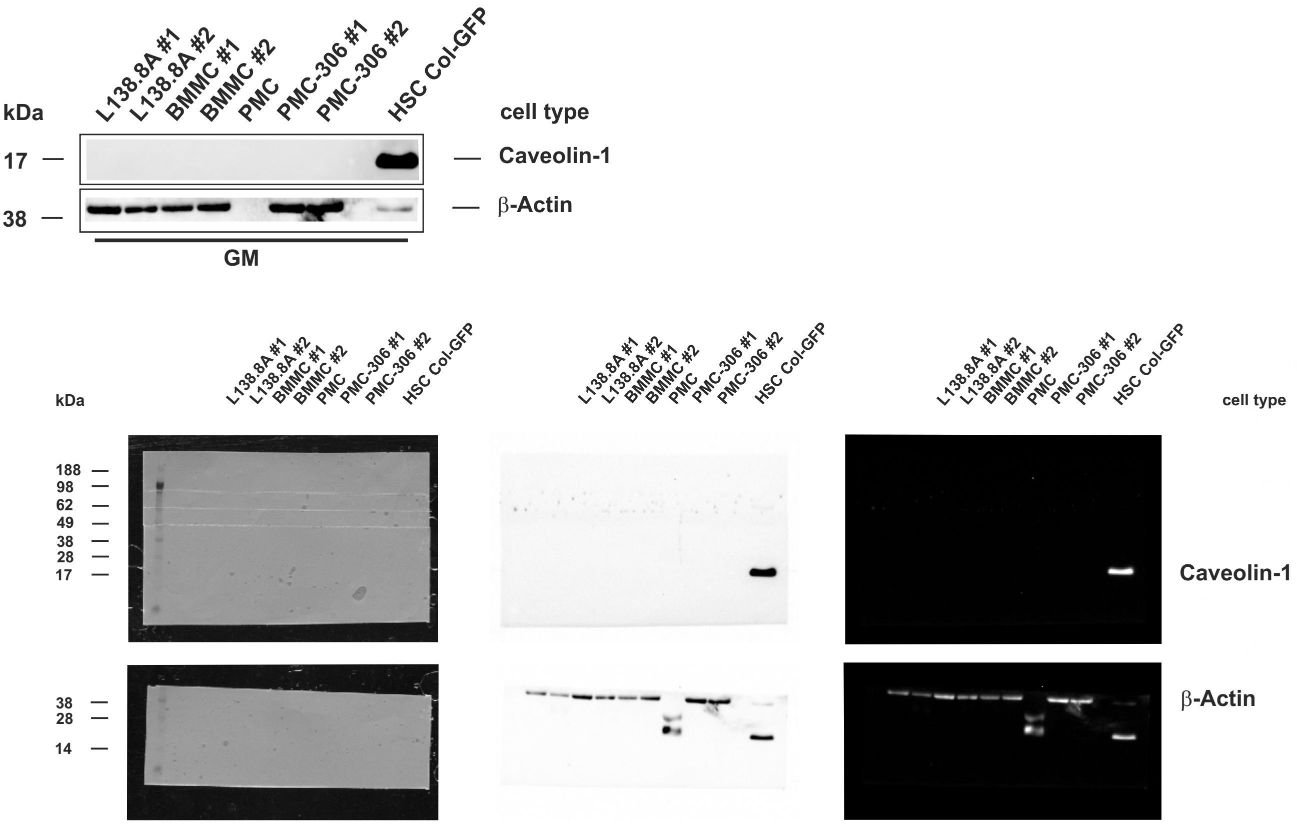

kDa
L138.8A #1
L138.8A #2
BMMC #1
BMMC #2
PMC
PMC-306 #1
PMC-306 #2
HSC Col-GFP
cell type
17
38
Caveolin-1
β-Actin
GM
188
98
62
49
28
14

Figure 4A

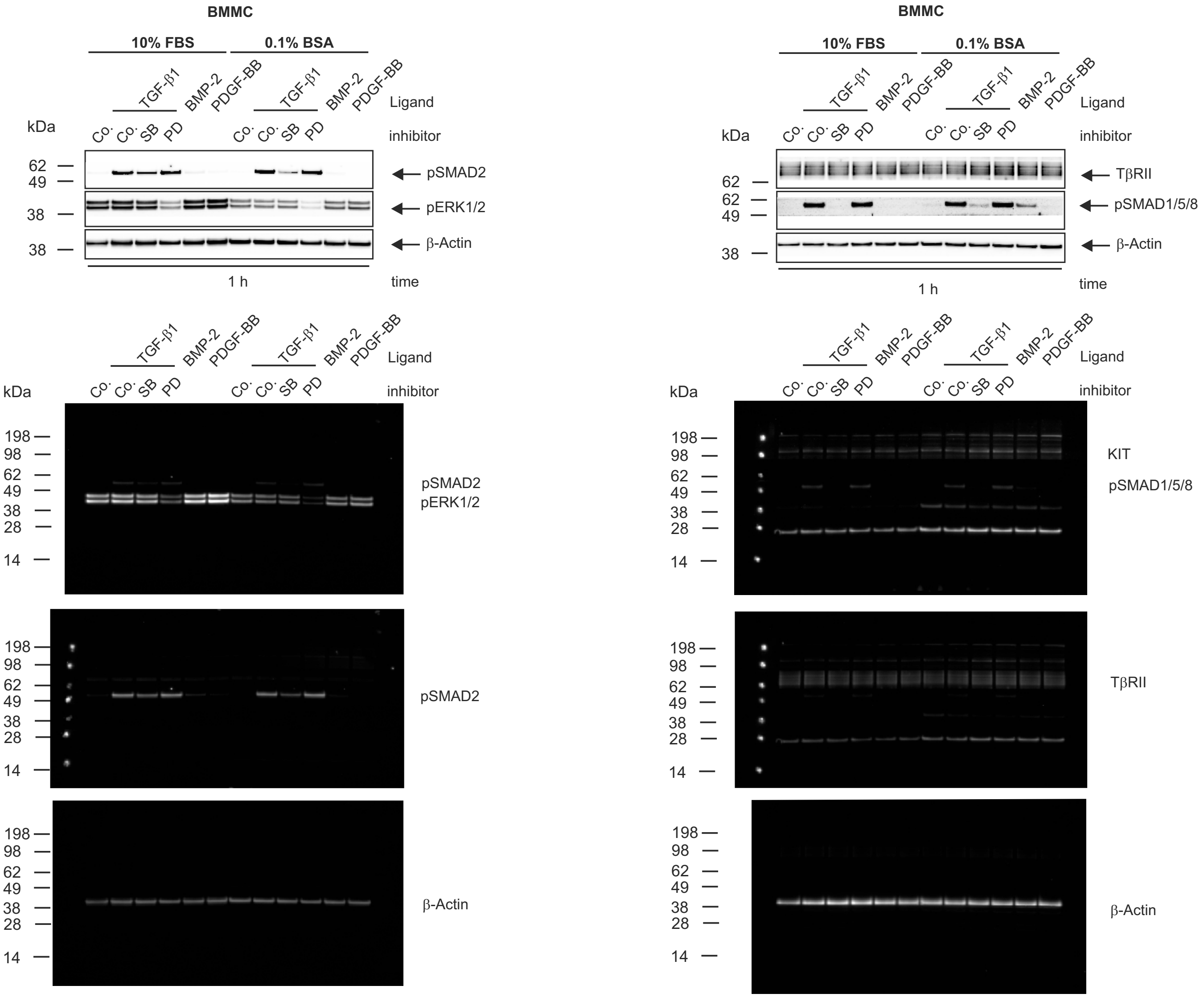
BMMC
10% FBS
0.1% BSA
TGF-β1
BMP-2
PDGF-BB
Ligand
kDa
Co.
SB
PD
inhibitor
62
49
38
pSMAD2
pERK1/2
β-Actin
1 h
time
TβRII
pSMAD1/5/8
198
98
28
14
KIT

# Figure 4B

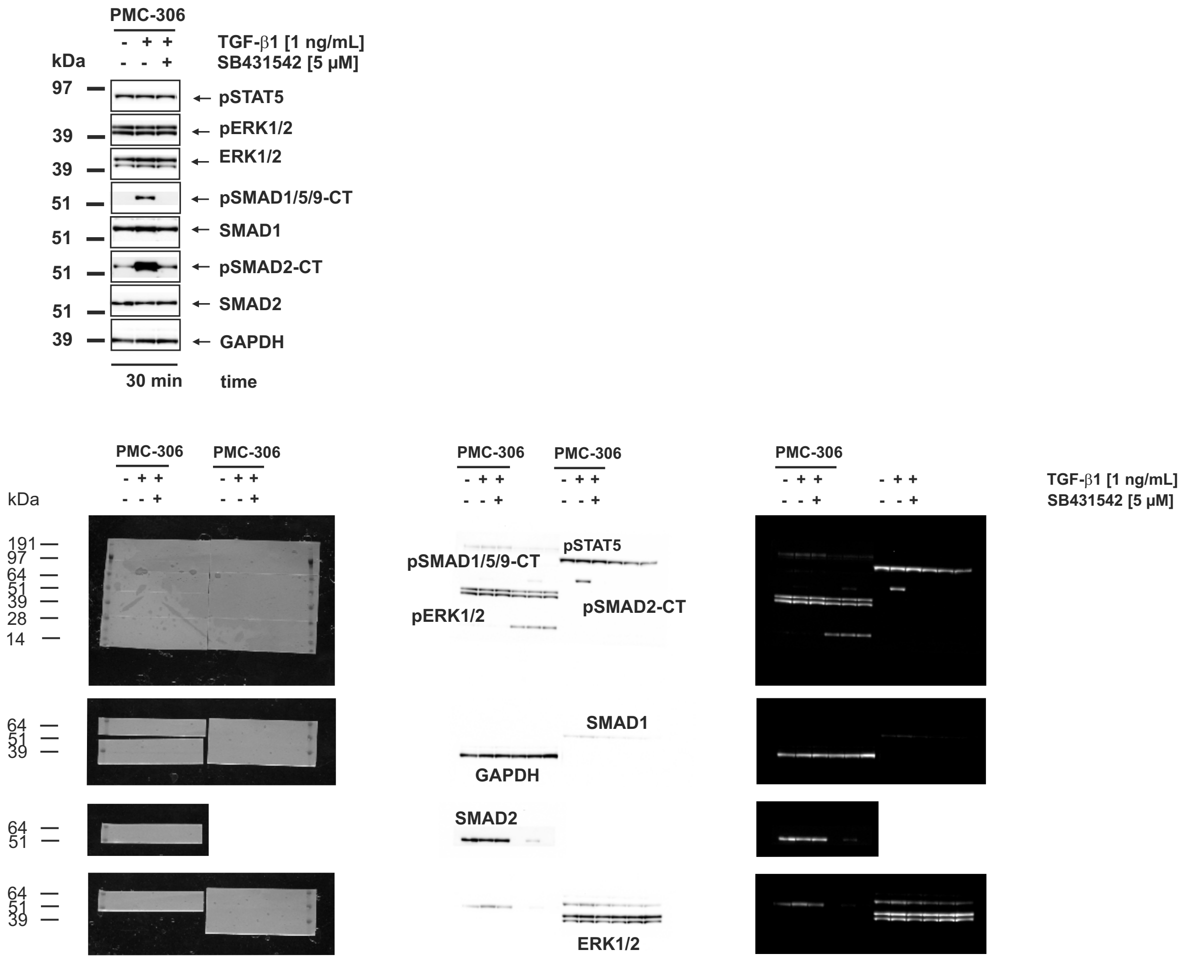

PMC-306
TGF-β1 [1 ng/mL]
SB431542 [5 μM]
kDa
97
39
39
51
51
51
51
39
pSTAT5
pERK1/2
ERK1/2
pSMAD1/5/9-CT
SMAD1
pSMAD2-CT
SMAD2
GAPDH
30 min
time
191
97
64
51
39
28
14
pSMAD1/5/9-CT
pSTAT5
pERK1/2
pSMAD2-CT
SMAD1
GAPDH
SMAD2
ERK1/2

Figure 4C

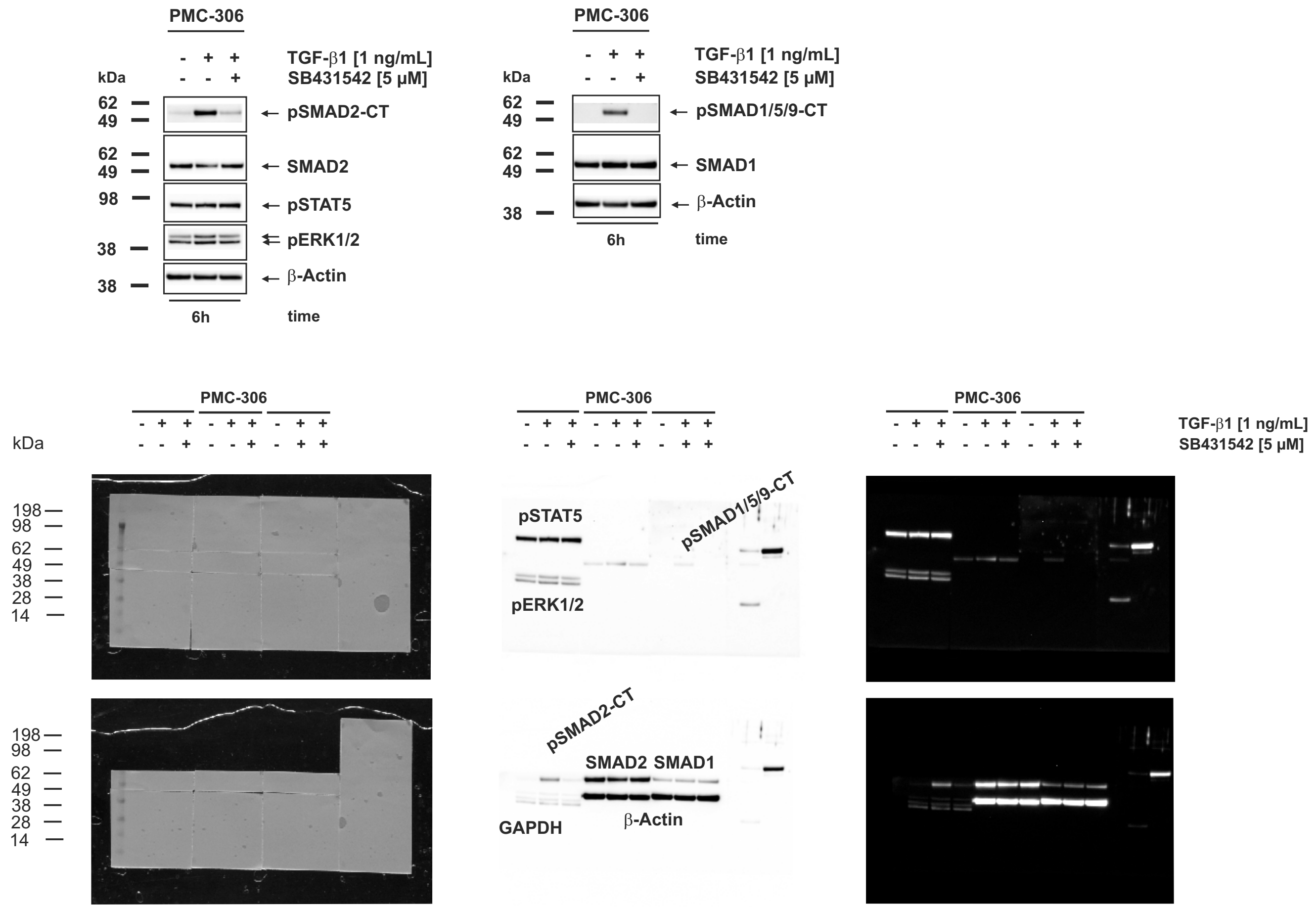
PMC-306
TGF-β1 [1 ng/mL]
SB431542 [5 µM]
kDa
62
49
pSMAD2-CT
SMAD2
98
pSTAT5
38
pERK1/2
β-Actin
6h
time
pSMAD1/5/9-CT
SMAD1
198
28
14
pSTAT5
pERK1/2
pSMAD2-CT
SMAD2 SMAD1
GAPDH
β-Actin

# Figure 5A

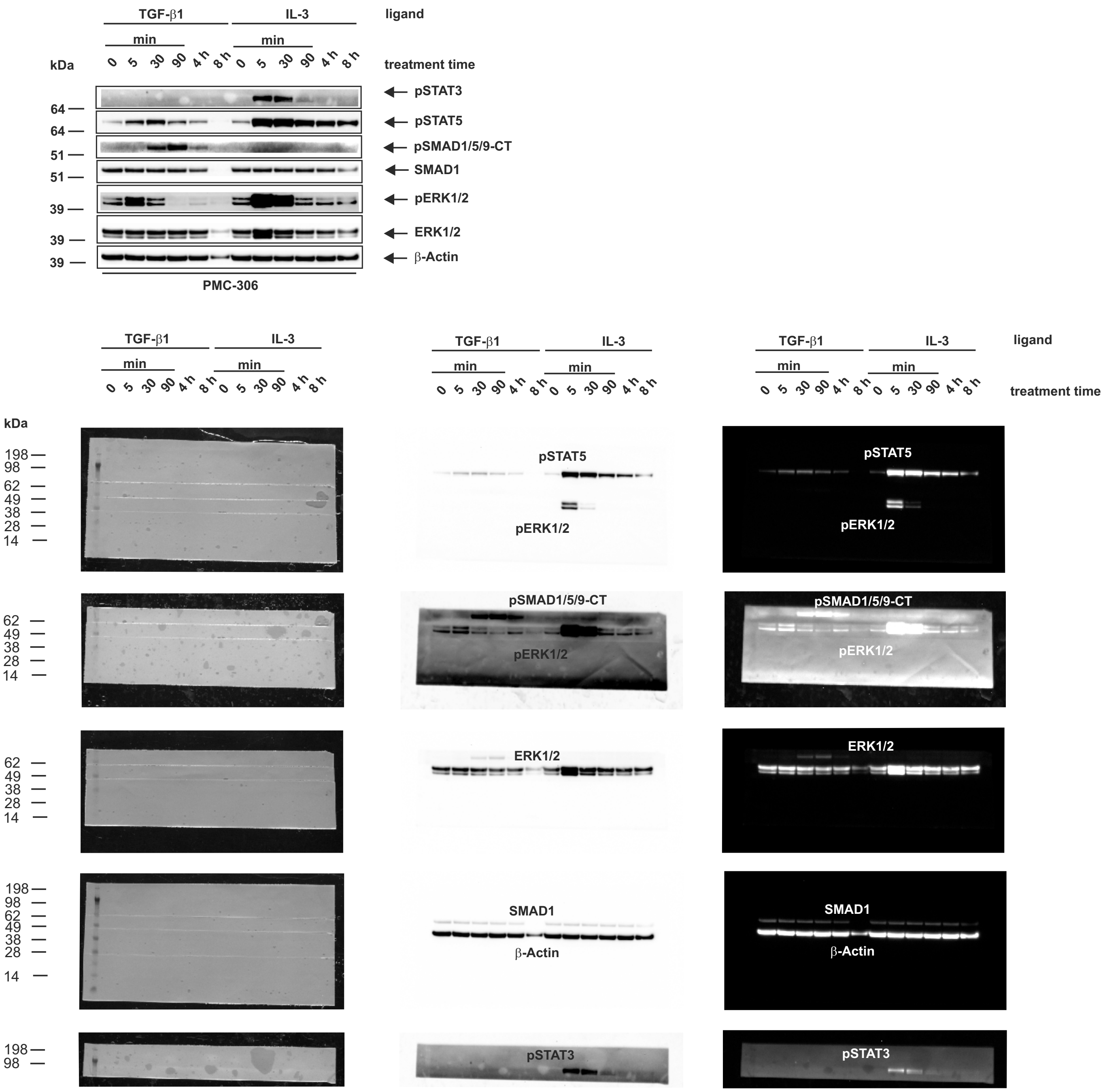
TGF-β1
IL-3
ligand
min
treatment time
kDa
0 5 30 90 4 h 8 h 0 5 30 90 4 h 8 h
64
64
51
51
39
39
39
pSTAT3
pSTAT5
pSMAD1/5/9-CT
SMAD1
pERK1/2
ERK1/2
β-Actin
PMC-306
198
98
62
49
38
28
14
pSTAT5
pERK1/2
pSMAD1/5/9-CT
ERK1/2
SMAD1
β-Actin
pSTAT3

# Figure 5A

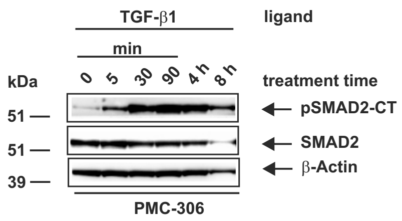

TGF-β1
ligand
min
kDa
0
5
30
90
4 h
8 h
treatment time
51
pSMAD2-CT
51
SMAD2
39
β-Actin
PMC-306


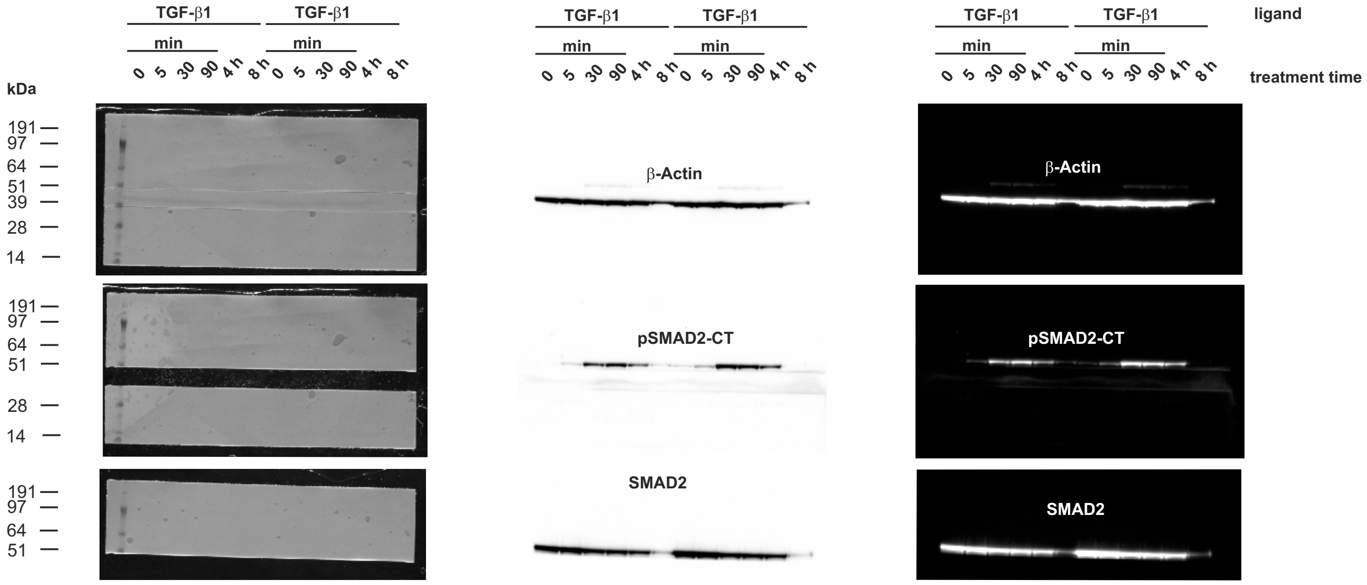

TGF-β1
TGF-β1
ligand
min
min
0
5
30
90
4 h
8 h
treatment time
kDa
191
97
64
51
39
28
14
β-Actin
pSMAD2-CT
SMAD2

Figure 5D

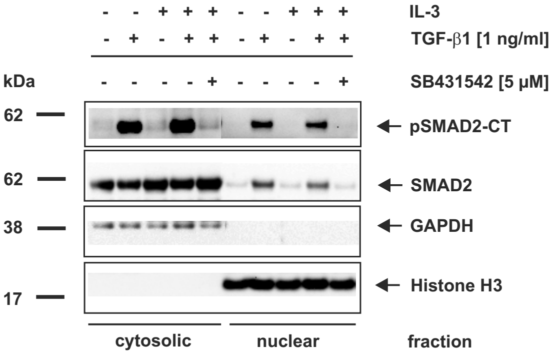
IL-3
TGF-β1 [1 ng/ml]
kDa
SB431542 [5 µM]
62
pSMAD2-CT
62
SMAD2
38
GAPDH
17
Histone H3
cytosolic
nuclear
fraction

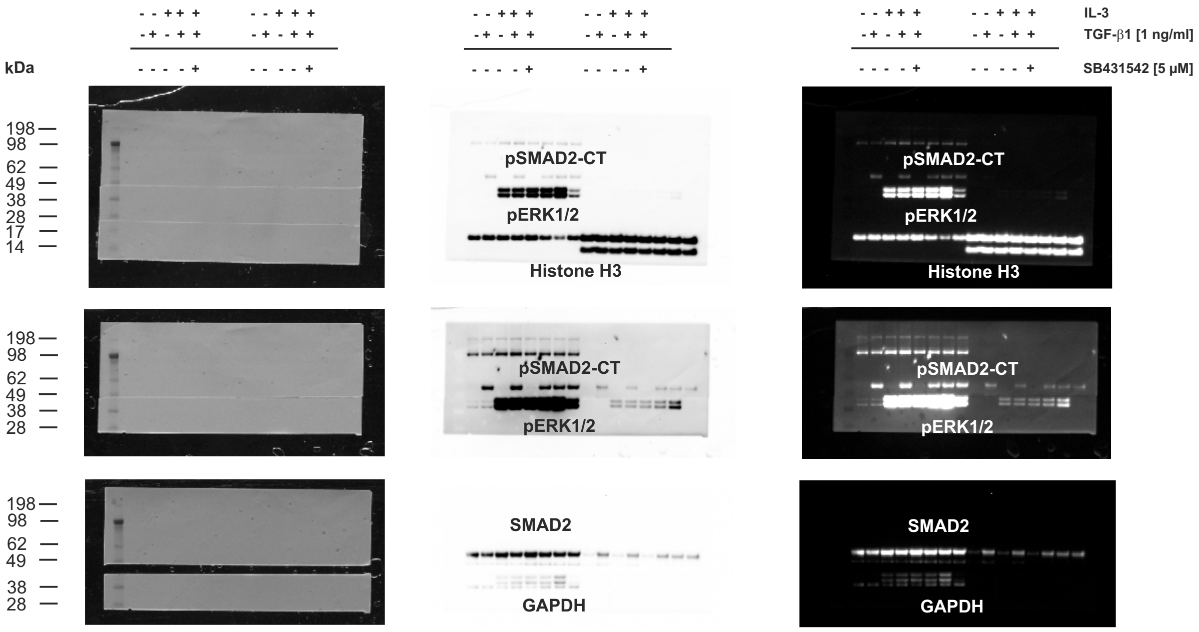
IL-3
TGF-β1 [1 ng/ml]
kDa
SB431542 [5 µM]
198
98
62
49
38
28
17
14
pSMAD2-CT
pERK1/2
Histone H3
SMAD2
GAPDH

Figure 5D

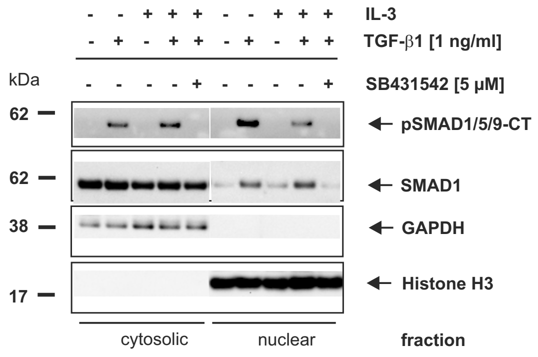
IL-3
TGF-β1 [1 ng/ml]
SB431542 [5 µM]
kDa
62
62
38
17
pSMAD1/5/9-CT
SMAD1
GAPDH
Histone H3
cytosolic
nuclear
fraction

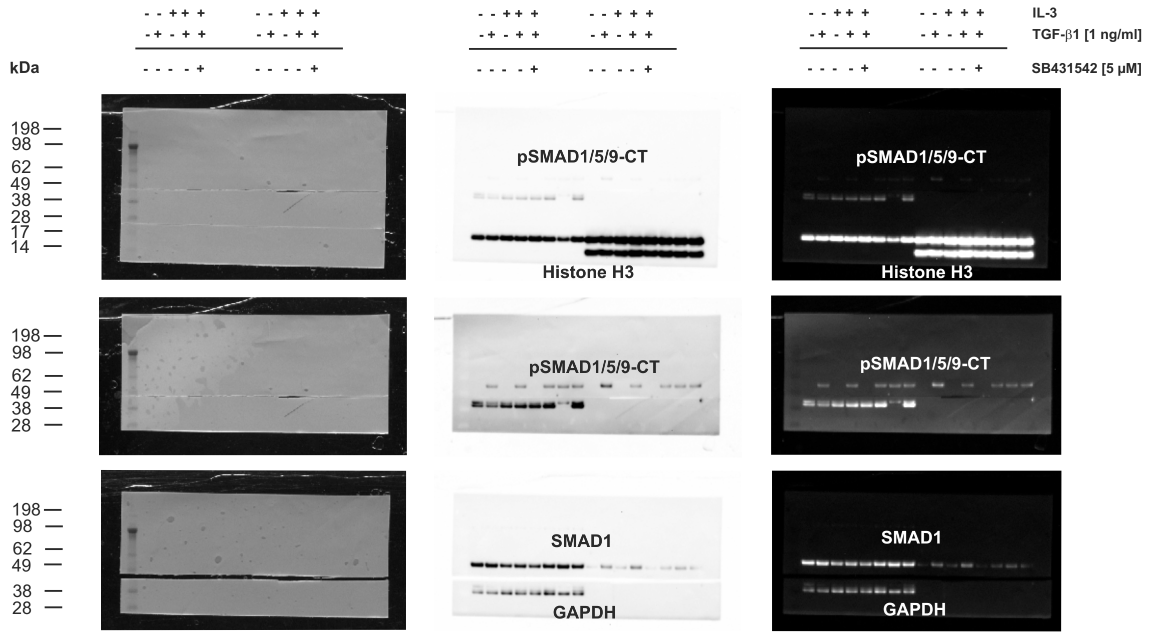
IL-3
TGF-β1 [1 ng/ml]
SB431542 [5 µM]
kDa
198
98
62
49
38
28
17
14
pSMAD1/5/9-CT
Histone H3
SMAD1
GAPDH

# Figure 6A

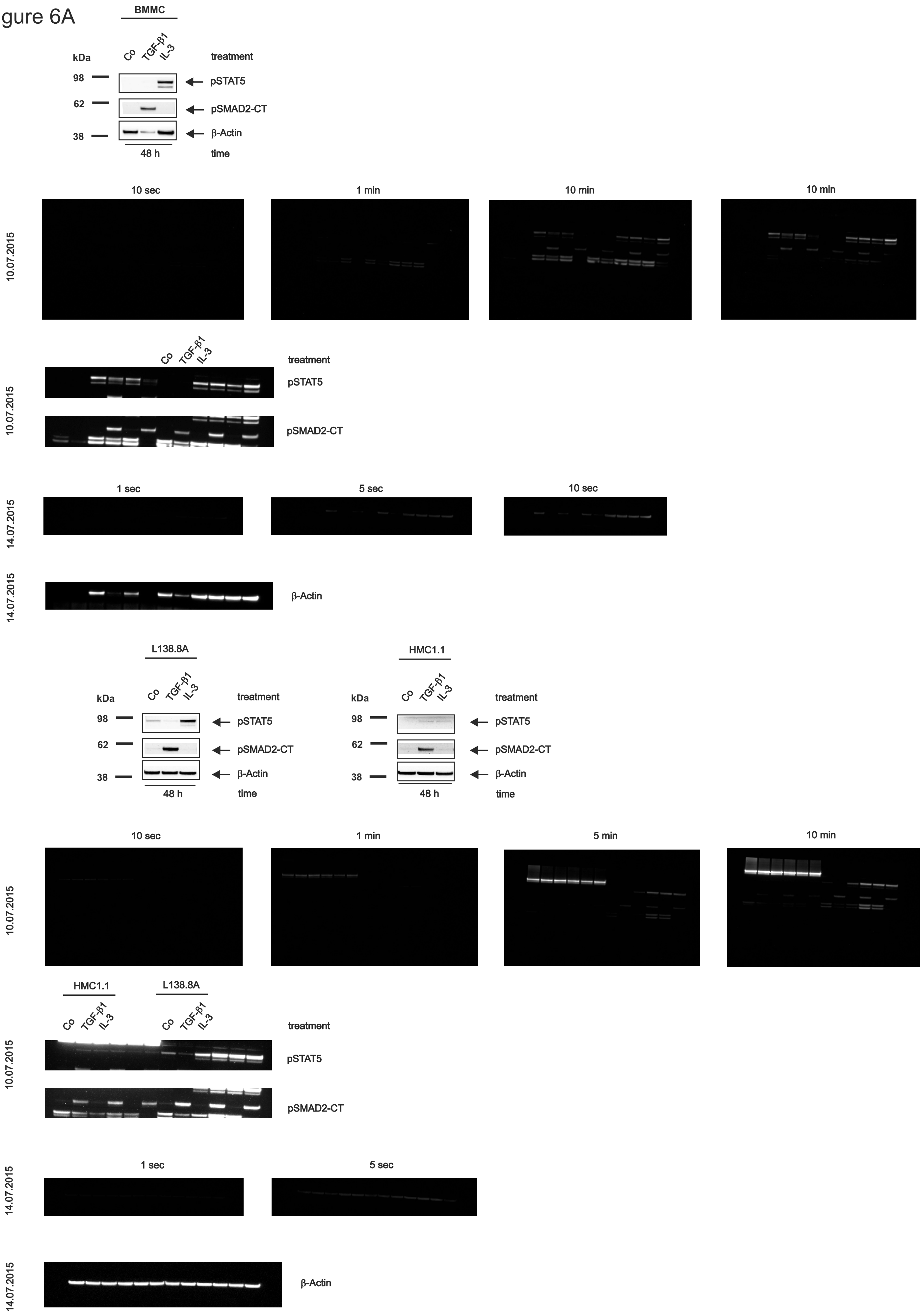
BMMC
kDa
Co
TGF-β1
IL-3
treatment
98
62
38
pSTAT5
pSMAD2-CT
β-Actin
48 h
time
10.07.2015
10 sec
1 min
10 min
10 min
10.07.2015
Co
TGF-β1
IL-3
treatment
pSTAT5
pSMAD2-CT
14.07.2015
1 sec
5 sec
10 sec
14.07.2015
β-Actin
L138.8A
kDa
Co
TGF-β1
IL-3
treatment
98
62
38
pSTAT5
pSMAD2-CT
β-Actin
48 h
time
HMC1.1
kDa
Co
TGF-β1
IL-3
treatment
98
62
38
pSTAT5
pSMAD2-CT
β-Actin
48 h
time
10.07.2015
10 sec
1 min
5 min
10 min
HMC1.1
L138.8A
Co
TGF-β1
IL-3
Co
TGF-β1
IL-3
treatment
10.07.2015
pSTAT5
pSMAD2-CT
14.07.2015
1 sec
5 sec
14.07.2015
β-Actin

Figure 6C

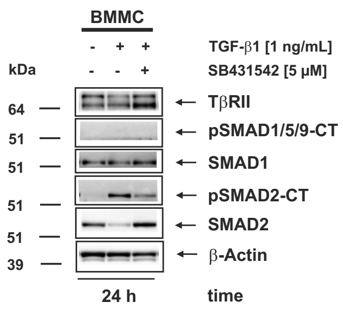
BMMC
TGF-β1 [1 ng/mL]
SB431542 [5 µM]
kDa
TβRII
pSMAD1/5/9-CT
SMAD1
pSMAD2-CT
SMAD2
β-Actin
24 h
time
64
51
39


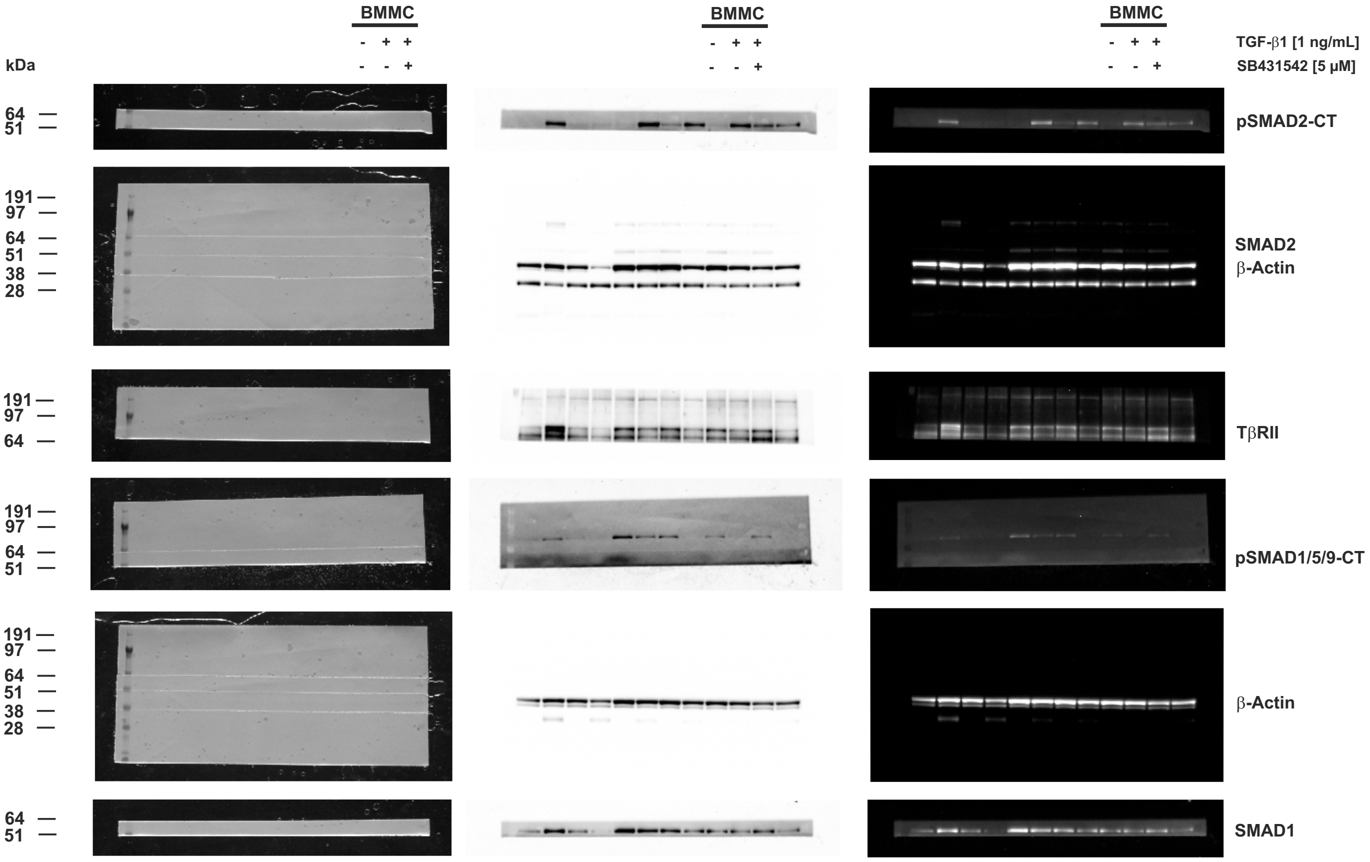
BMMC
TGF-β1 [1 ng/mL]
SB431542 [5 µM]
kDa
pSMAD2-CT
SMAD2
β-Actin
TβRII
pSMAD1/5/9-CT
β-Actin
SMAD1
191
97
64
51
38
28

Figure 6D

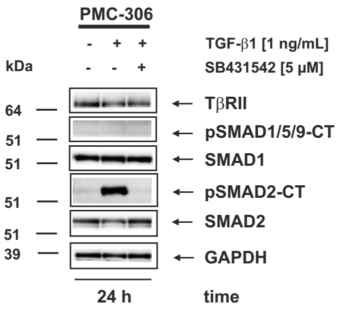

PMC-306
TGF-β1 [1 ng/mL]
SB431542 [5 µM]
kDa
64
51
51
51
51
39
TβRII
pSMAD1/5/9-CT
SMAD1
pSMAD2-CT
SMAD2
GAPDH
24 h
time


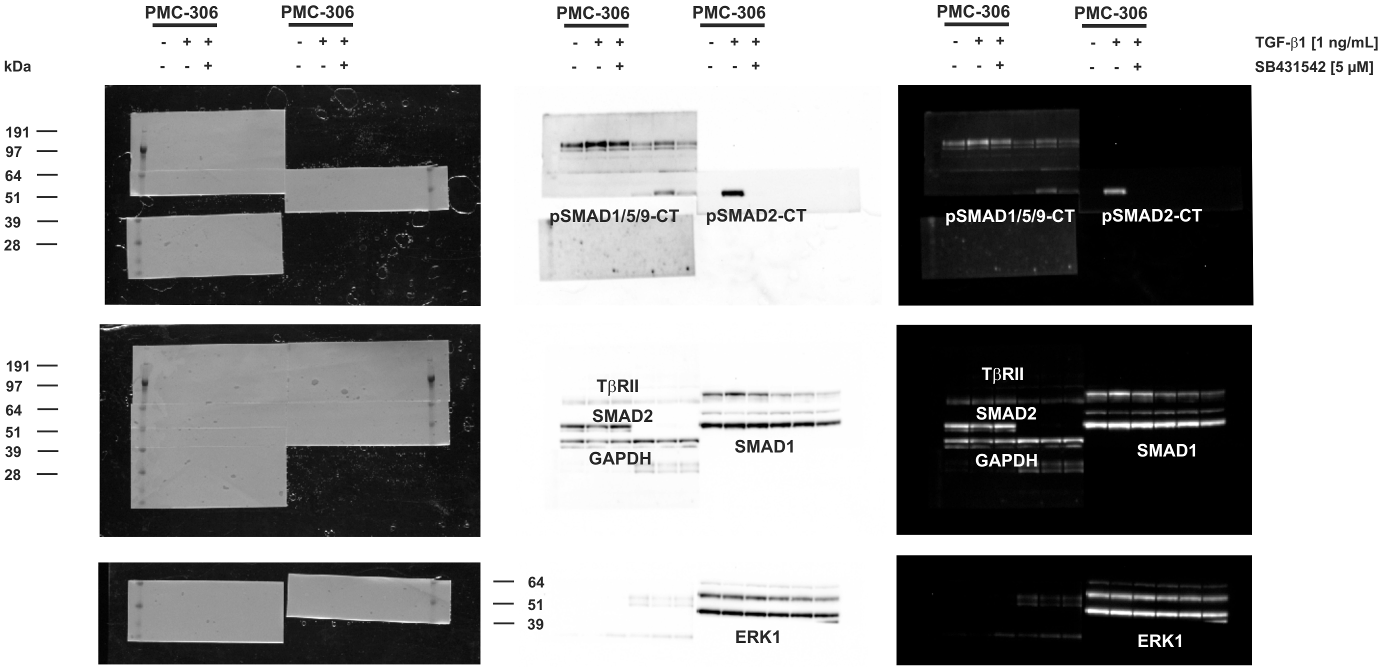

PMC-306
PMC-306
TGF-β1 [1 ng/mL]
SB431542 [5 µM]
kDa
191
97
64
51
39
28
pSMAD1/5/9-CT
pSMAD2-CT
TβRII
SMAD2
GAPDH
SMAD1
64
51
39
ERK1

Figure 7E

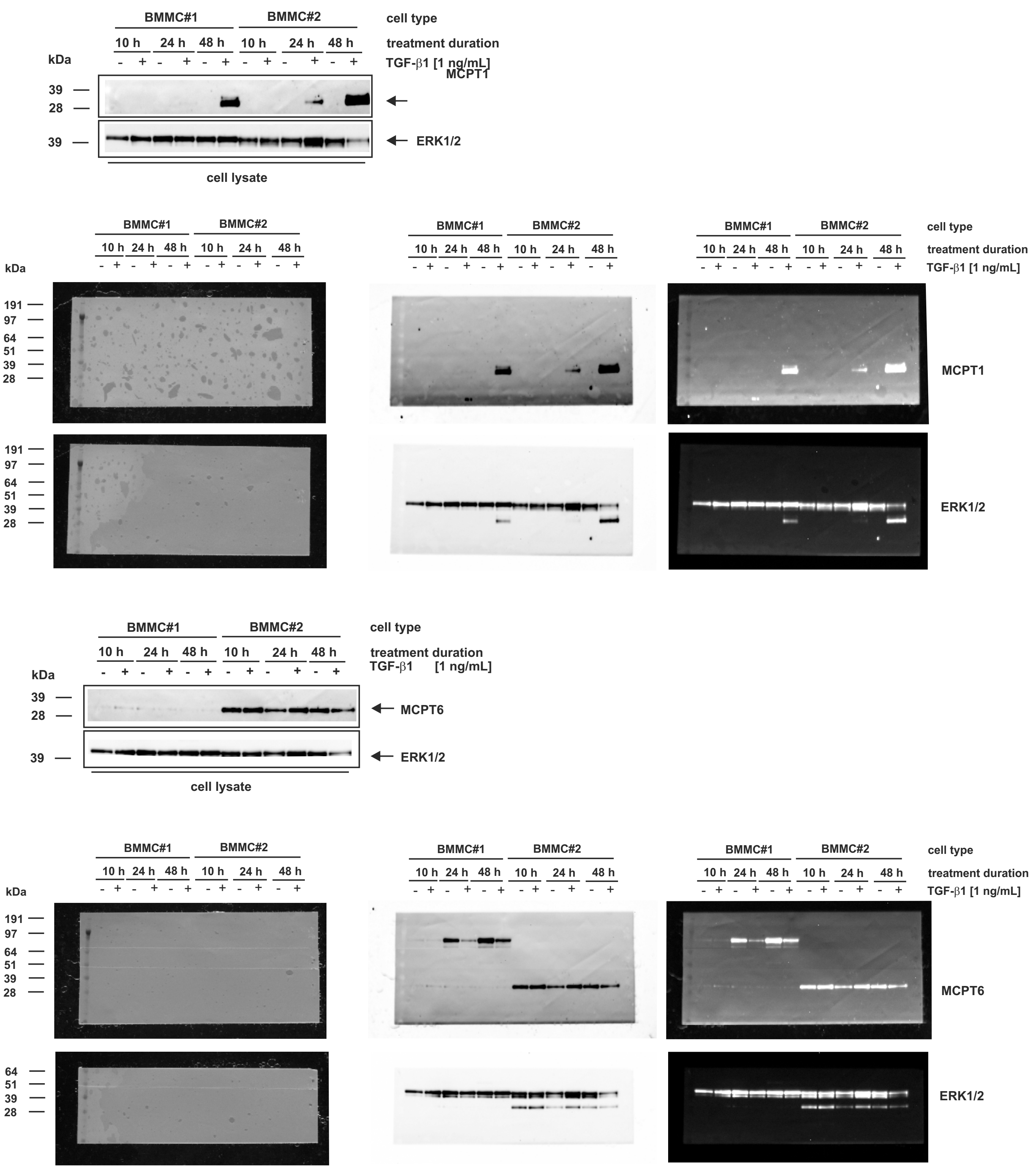

BMMC#1
BMMC#2
cell type
10 h
24 h
48 h
treatment duration
kDa
TGF-β1 [1 ng/mL]
MCPT1
39
28
ERK1/2
cell lysate
191
97
64
51
MCPT6

# Figure 7F

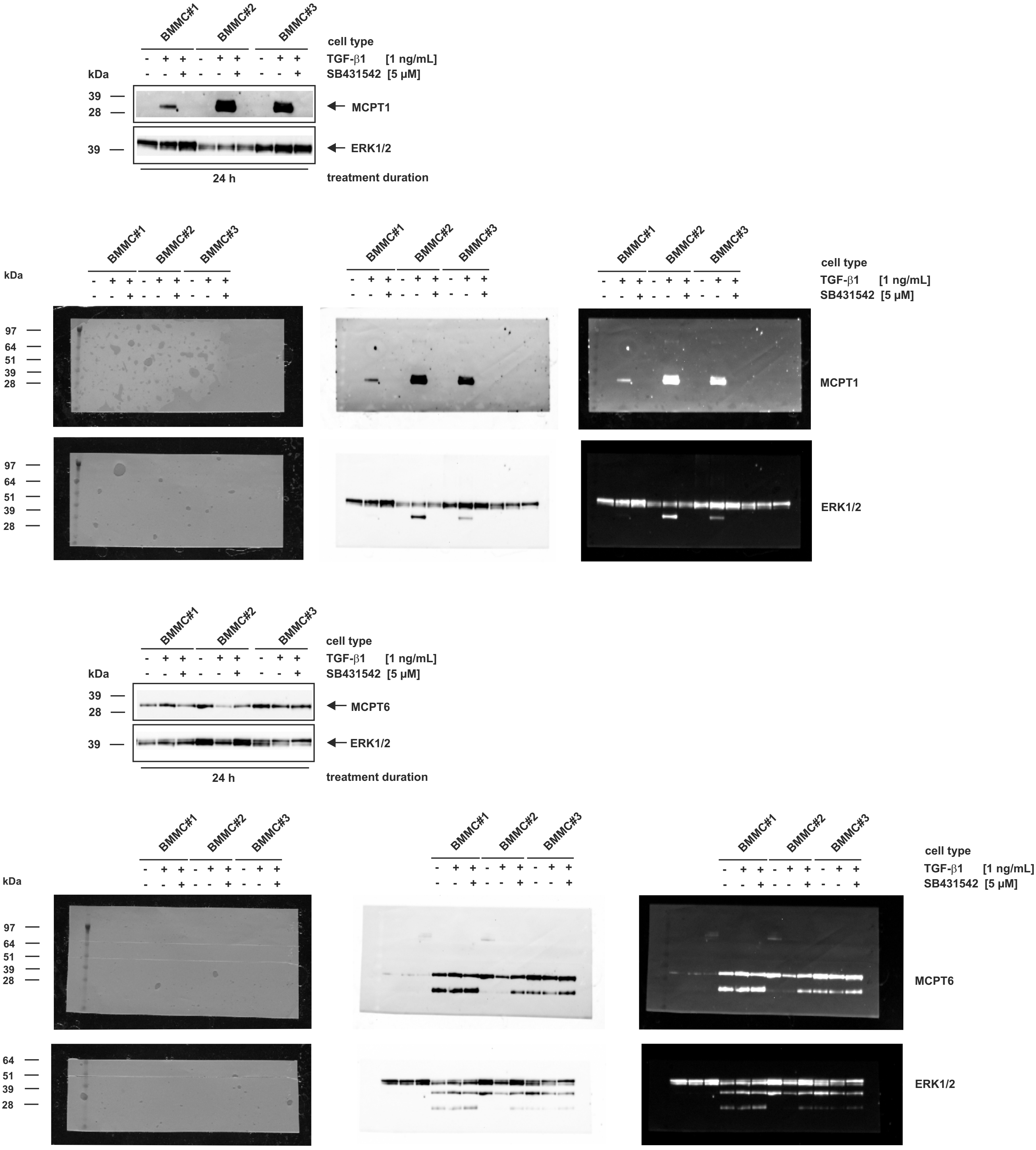
BMMC#1
BMMC#2
BMMC#3
cell type
TGF-β1 [1 ng/mL]
SB431542 [5 µM]
kDa
39
28
MCPT1
ERK1/2
24 h
treatment duration
97
64
51
MCPT6

Figure 7G

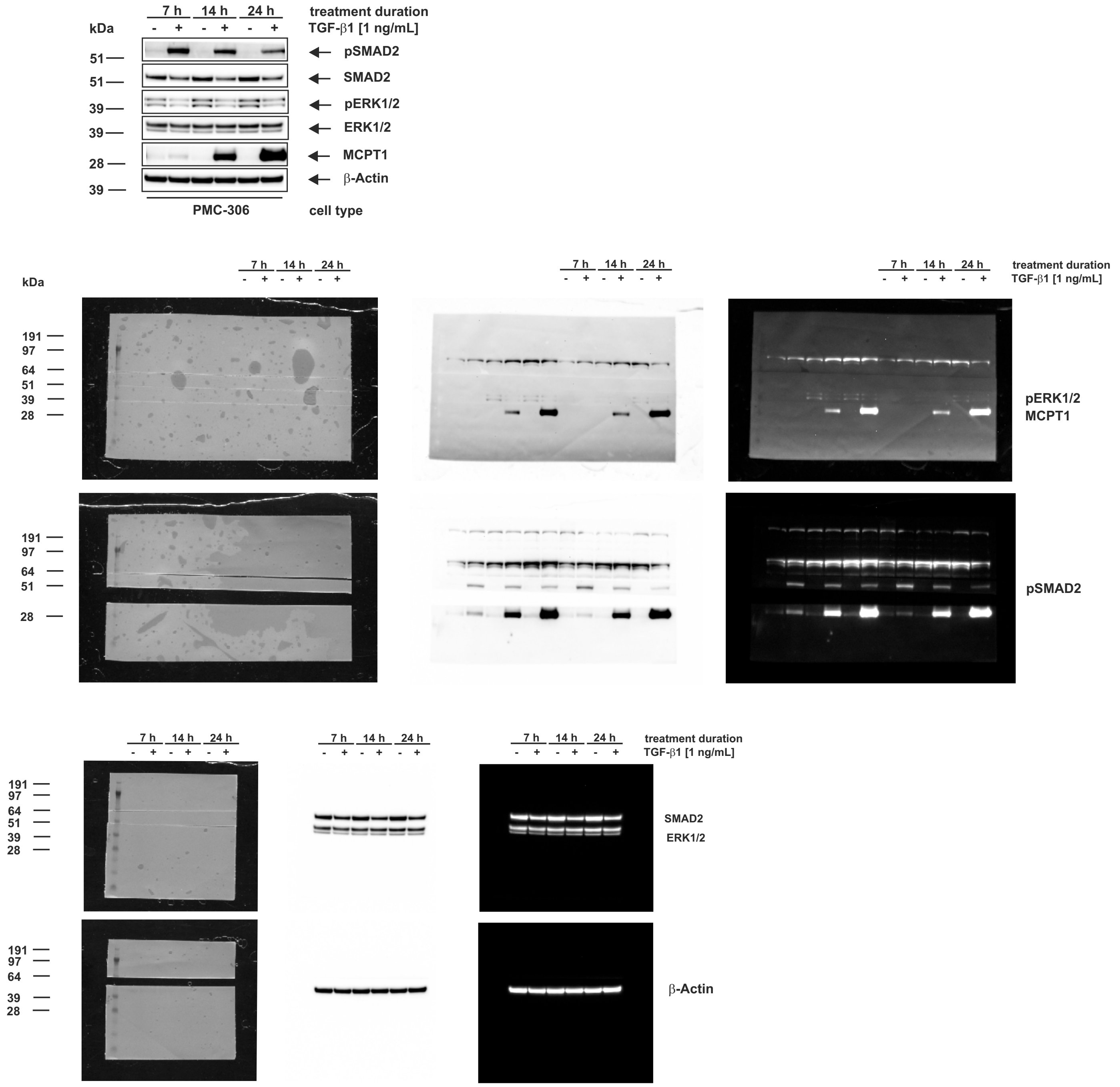
7 h
14 h
24 h
treatment duration
kDa
- + - + - +
TGF-β1 [1 ng/mL]
51
pSMAD2
51
SMAD2
39
pERK1/2
39
ERK1/2
28
MCPT1
39
β-Actin
PMC-306
cell type
191
97
64
51
39
28
pERK1/2
MCPT1
pSMAD2
SMAD2
ERK1/2
β-Actin

Figure 7H

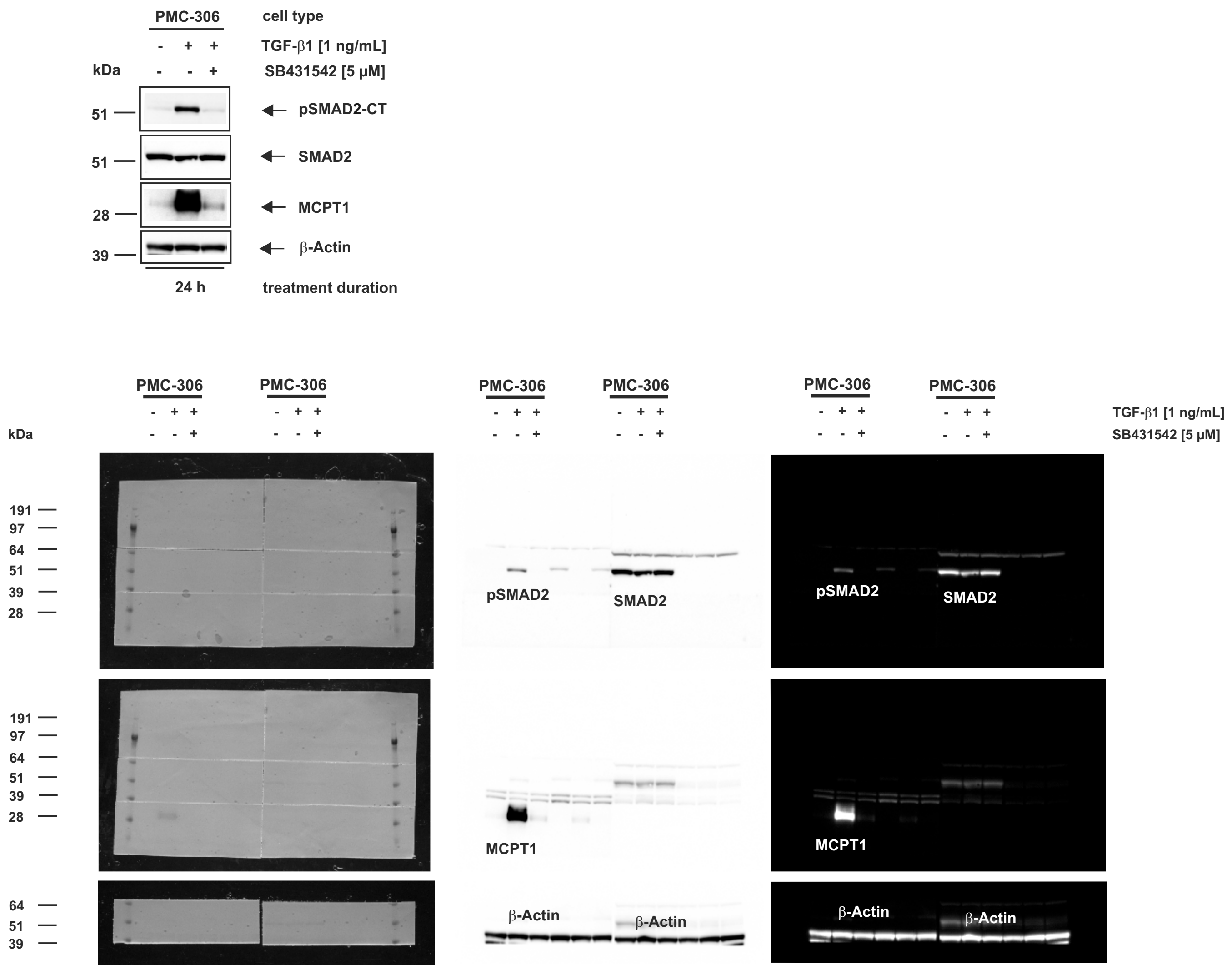

PMC-306
cell type
TGF-β1 [1 ng/mL]
SB431542 [5 µM]
kDa
51
pSMAD2-CT
SMAD2
28
MCPT1
39
β-Actin
24 h
treatment duration
PMC-306
191
97
64
51
39
28
pSMAD2
SMAD2
MCPT1
β-Actin

# Figure 8B

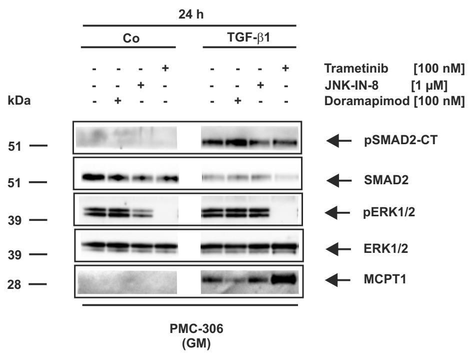

24 h
Co
TGF-β1
Trametinib [100 nM]
JNK-IN-8 [1 µM]
Doramapimod [100 nM]
kDa
51
51
39
39
28
pSMAD2-CT
SMAD2
pERK1/2
ERK1/2
MCPT1
PMC-306
(GM)


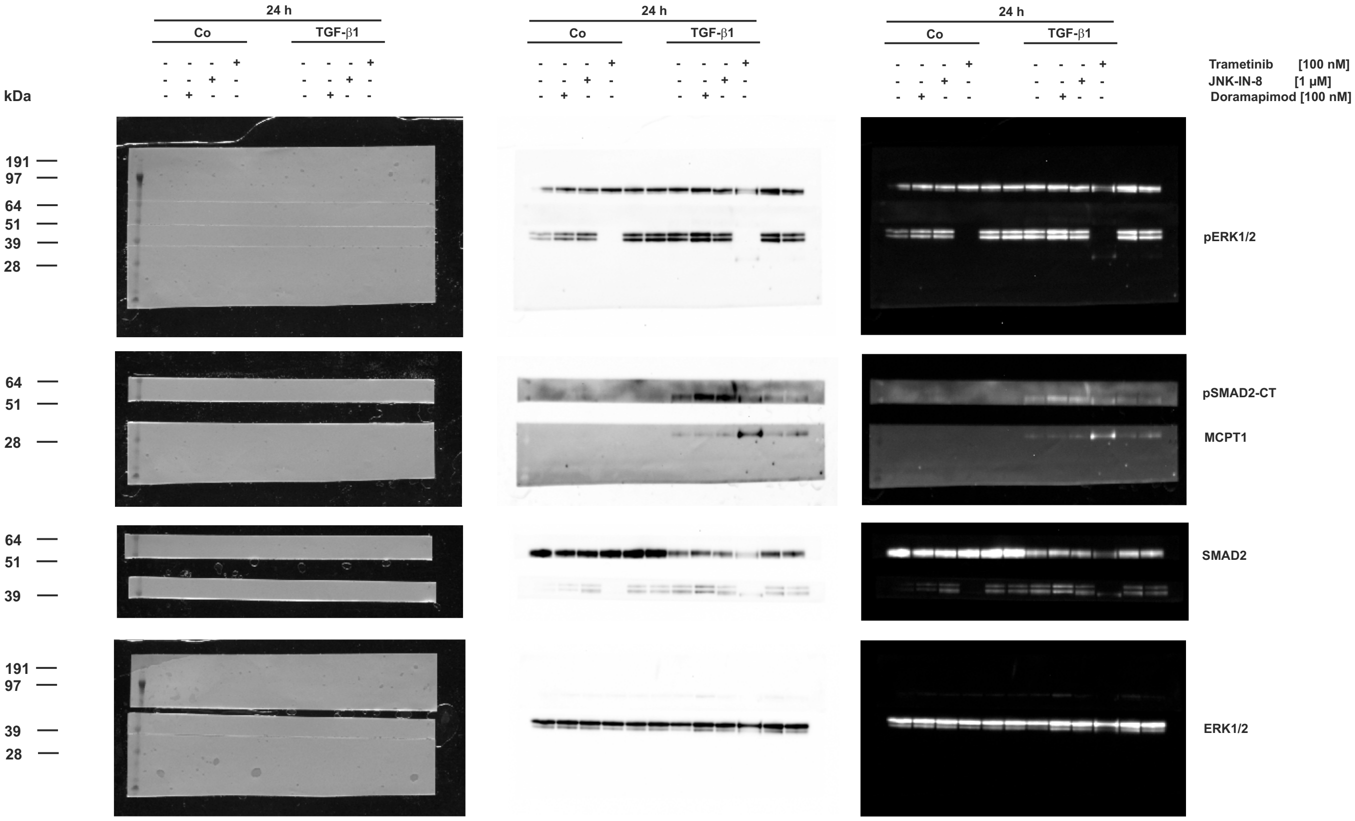

24 h
Co
TGF-β1
Trametinib [100 nM]
JNK-IN-8 [1 µM]
Doramapimod [100 nM]
kDa
191
97
64
51
39
28
pERK1/2
pSMAD2-CT
MCPT1
SMAD2
ERK1/2

Figure 8E

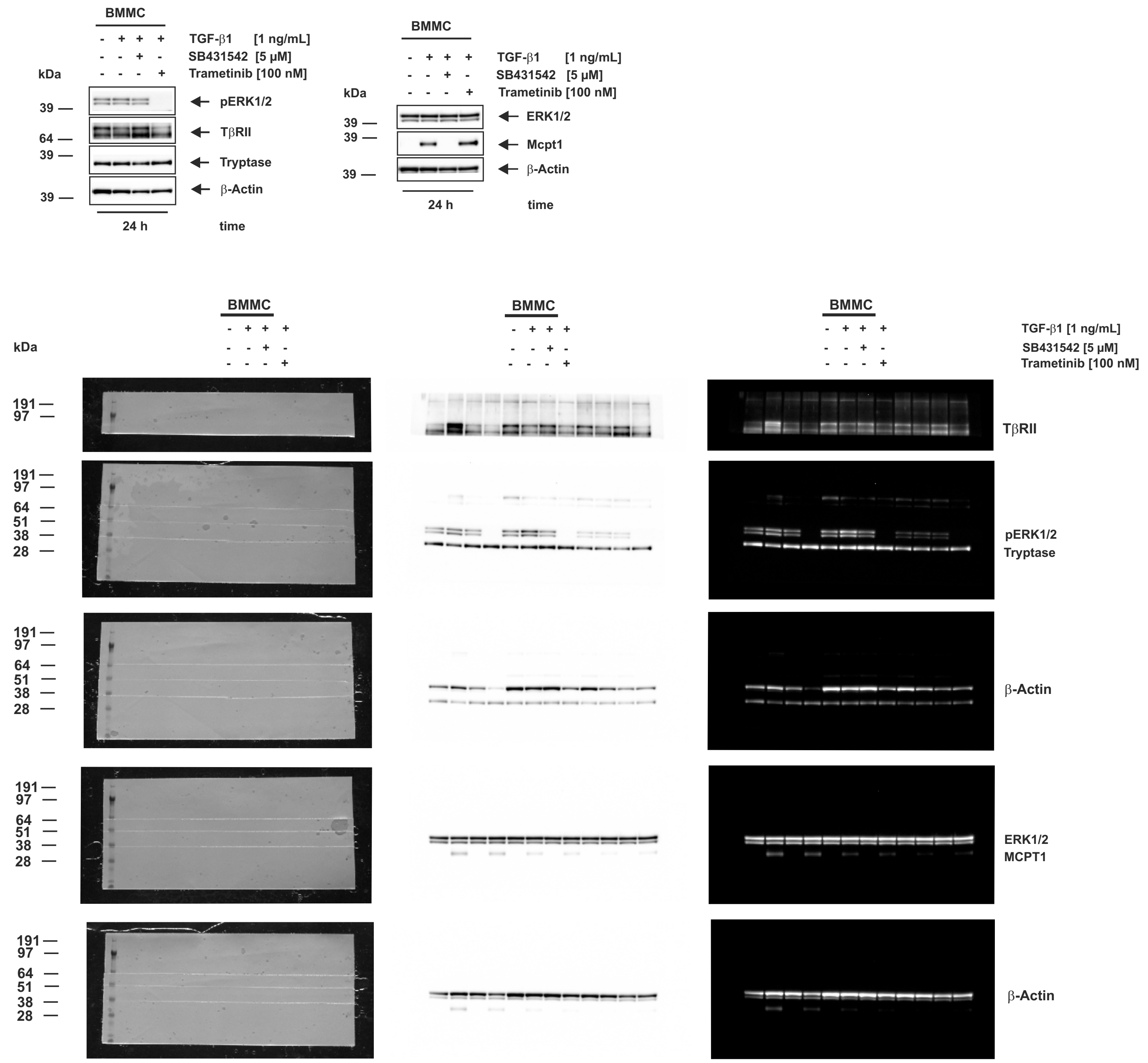
BMMC
TGF-β1 [1 ng/mL]
SB431542 [5 µM]
Trametinib [100 nM]
kDa
39
64
39
39
pERK1/2
TβRII
Tryptase
β-Actin
24 h
time
BMMC
TGF-β1 [1 ng/mL]
SB431542 [5 µM]
Trametinib [100 nM]
kDa
39
39
39
ERK1/2
Mcpt1
β-Actin
24 h
time
BMMC
BMMC
BMMC
TGF-β1 [1 ng/mL]
SB431542 [5 µM]
Trametinib [100 nM]
kDa
191
97
64
51
38
28
TβRII
pERK1/2
Tryptase
β-Actin
ERK1/2
MCPT1
β-Actin

Supplemental Figure 1A

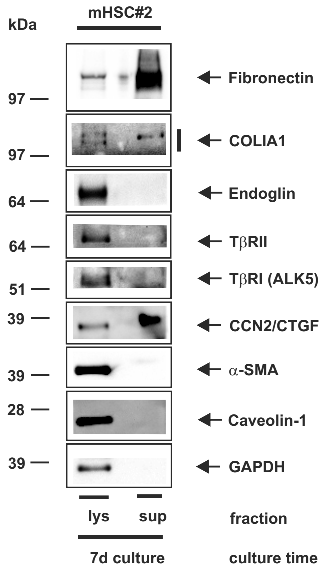


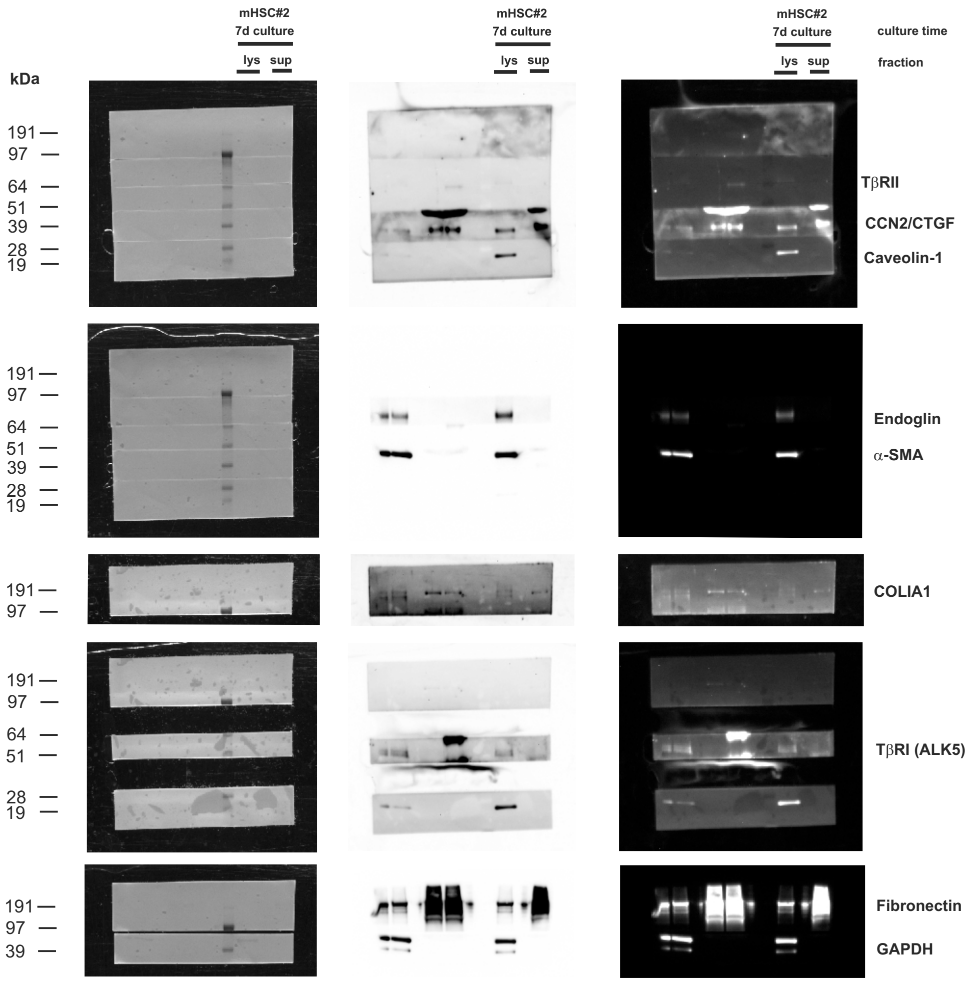

Supplemental Figure 1B

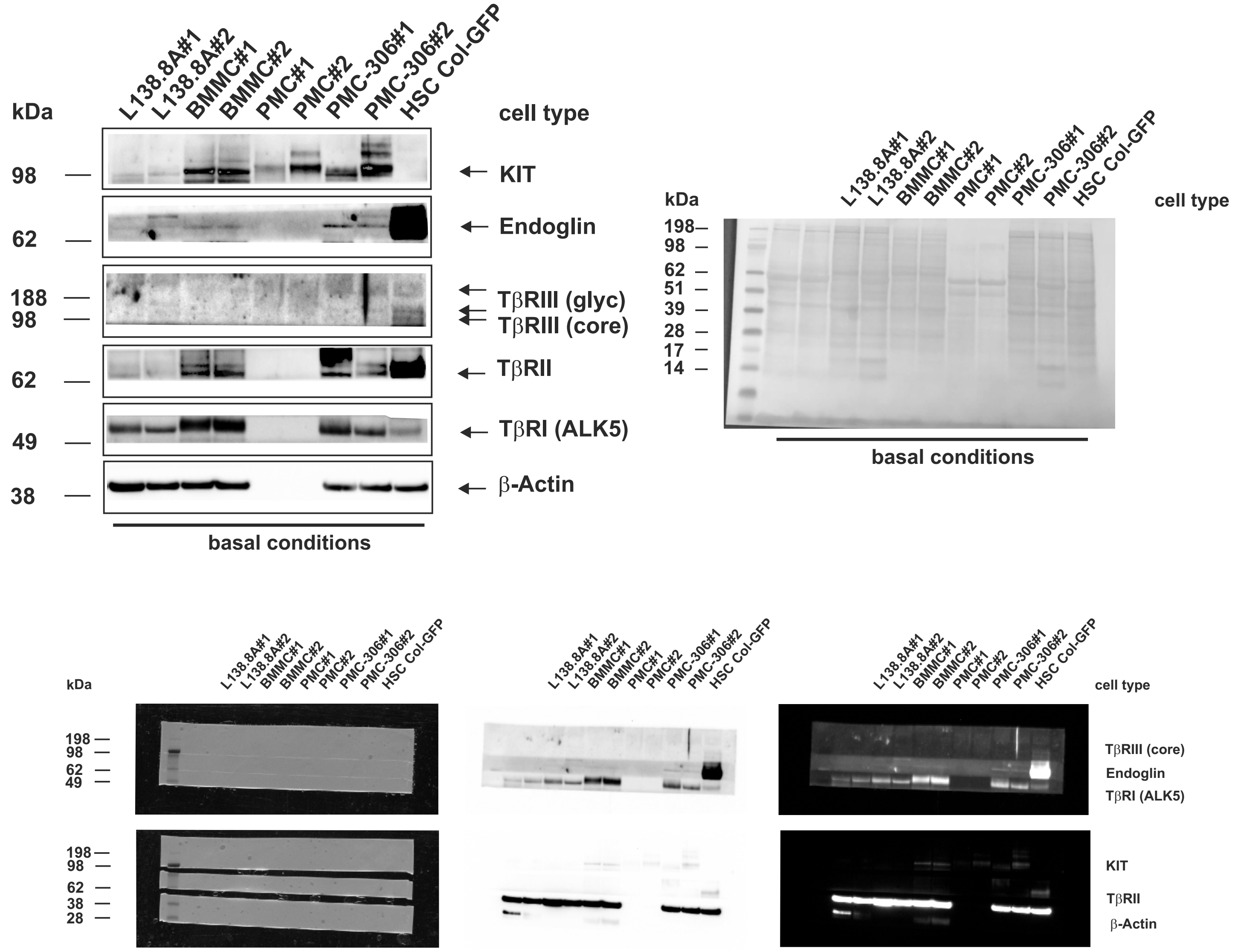

# Supplementary Figure 2C

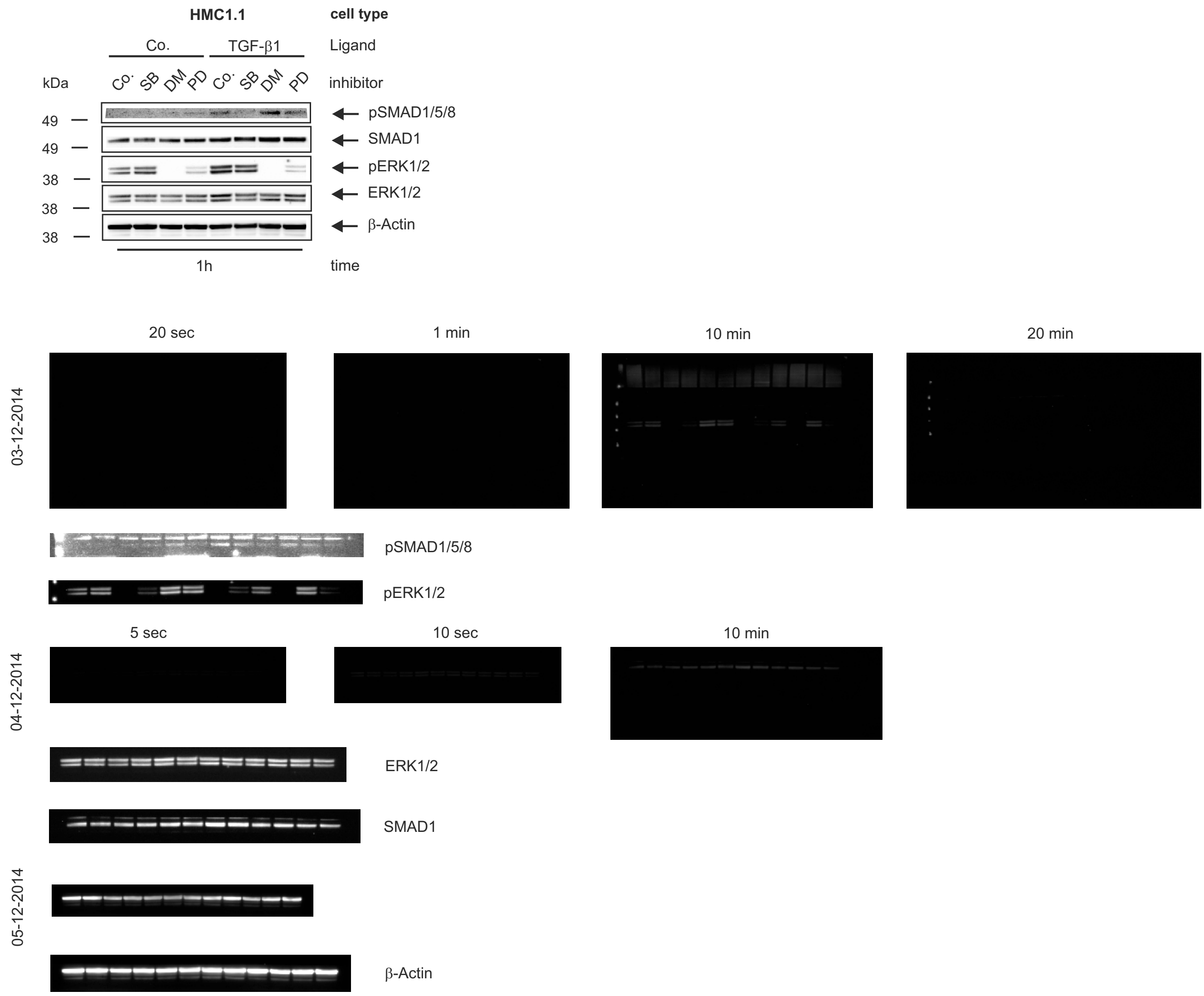


# Supplementary Figure 2A

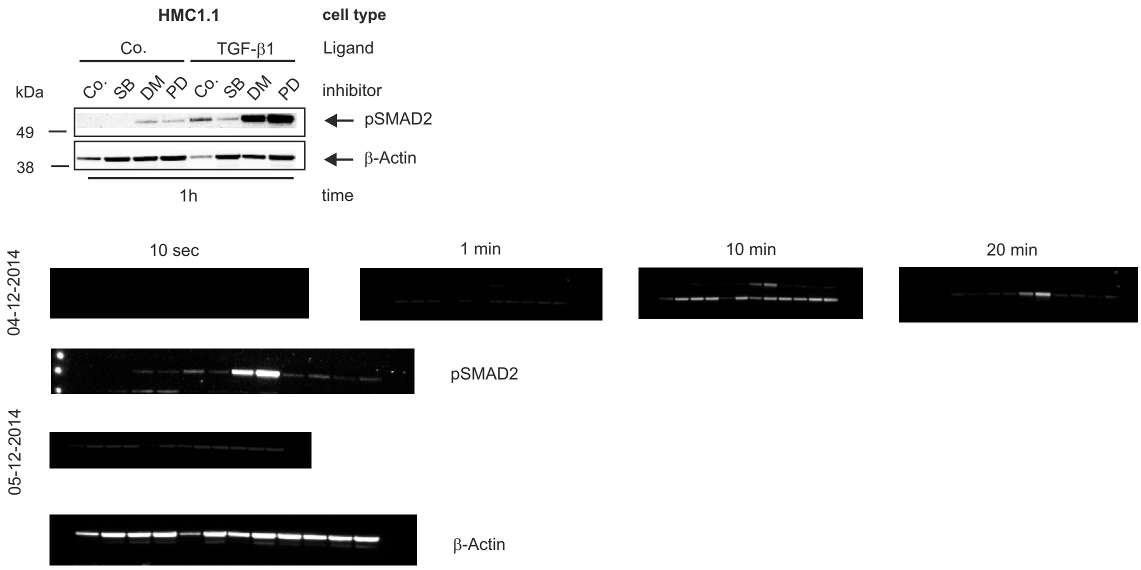

# Supplementary Figure 2B

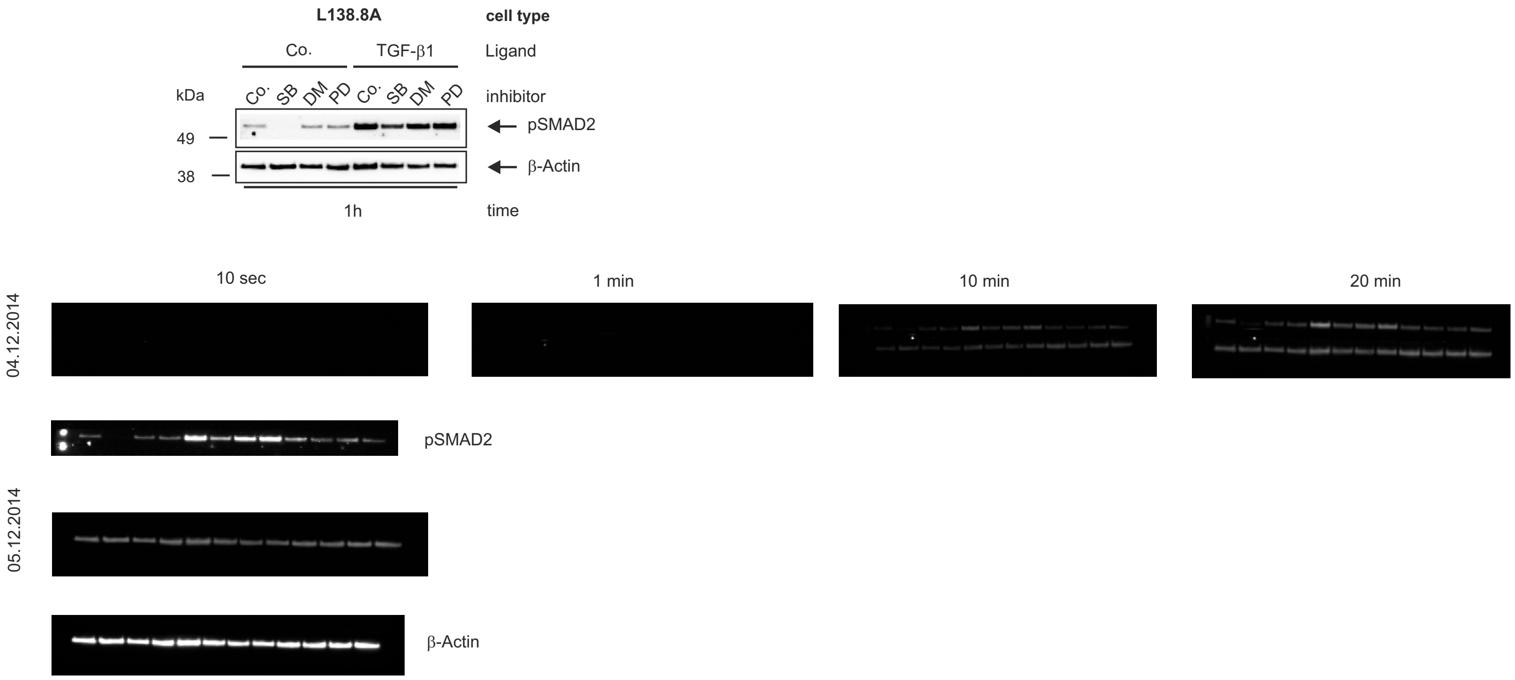

# Supplementary Figure 2E

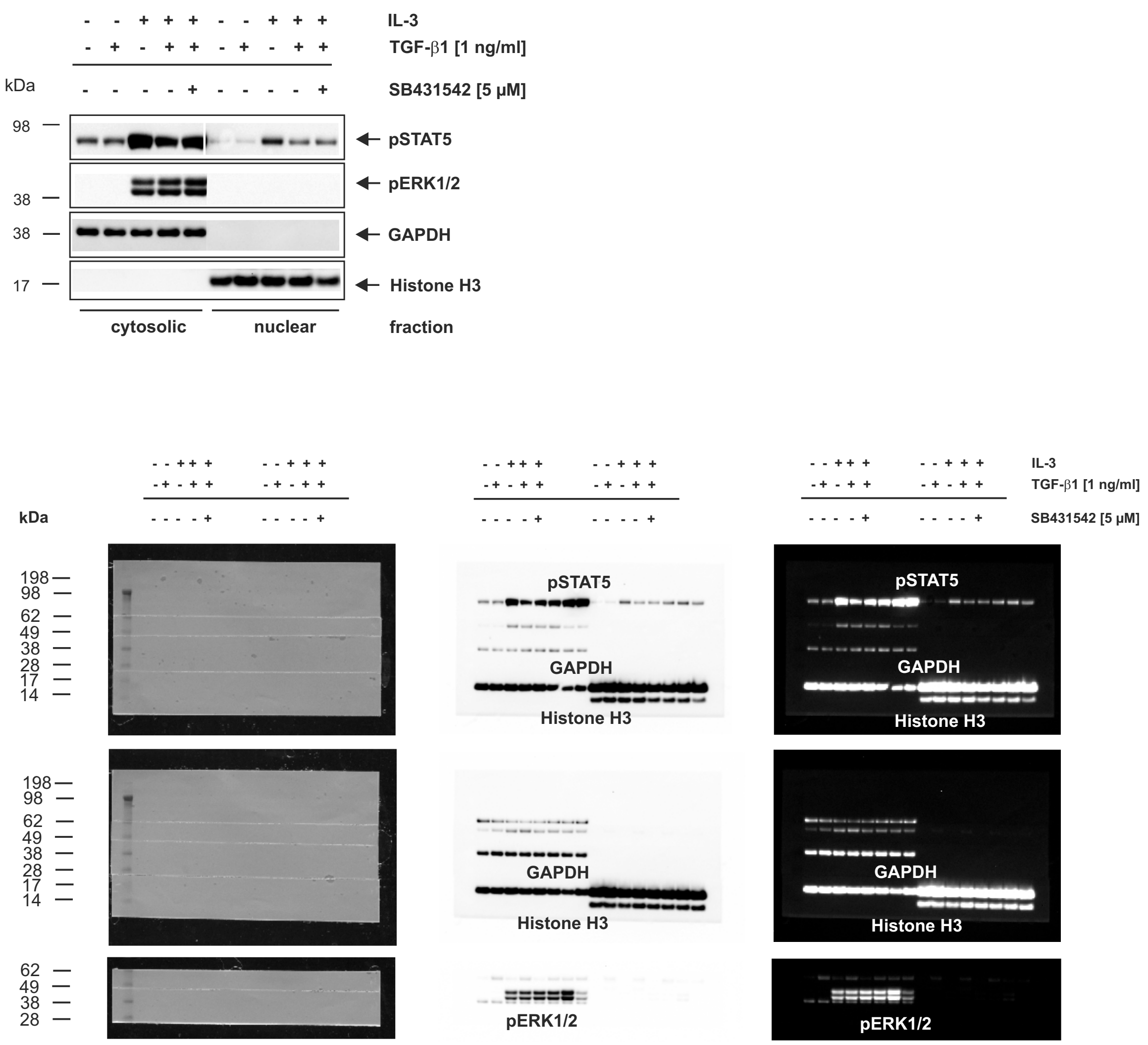

Suppl. Figure 3C

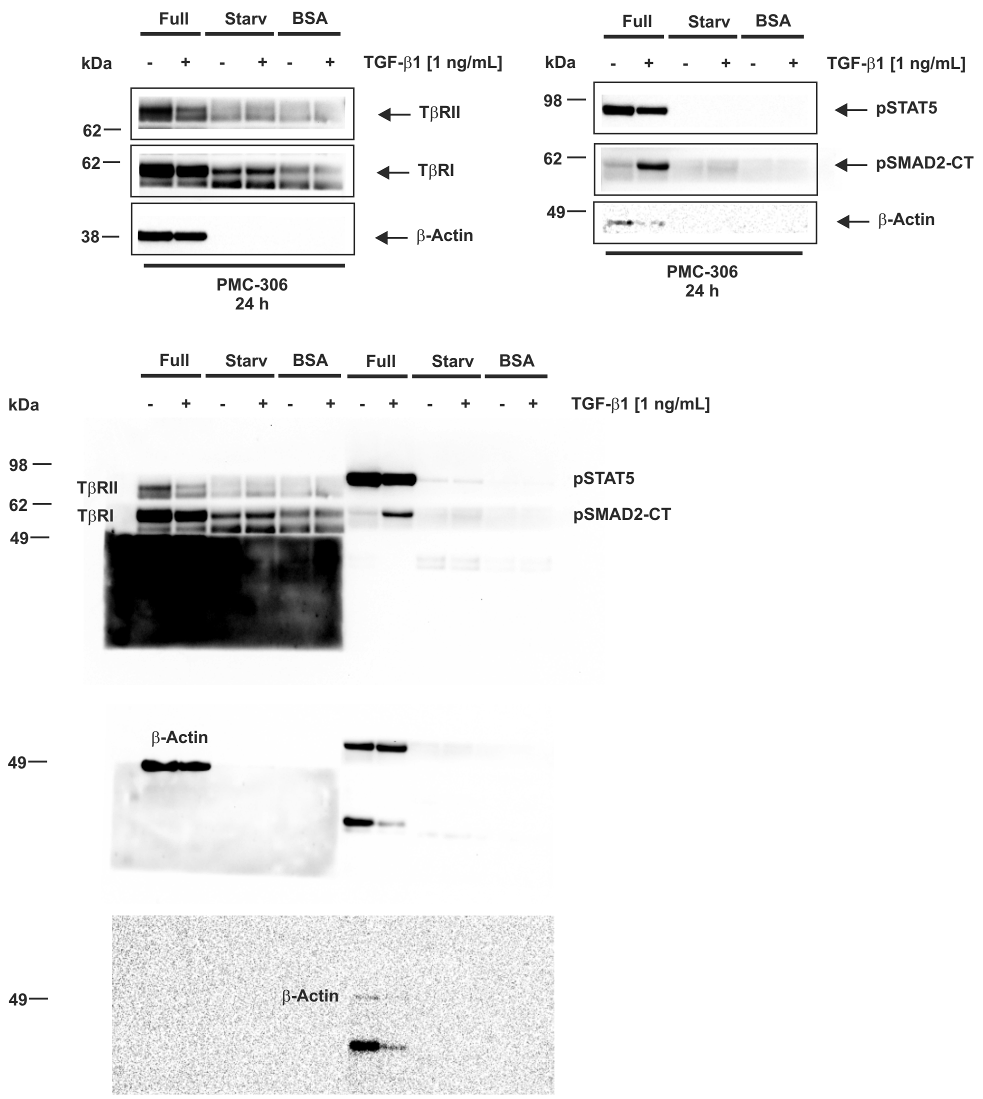

Suppl. Figure 3D

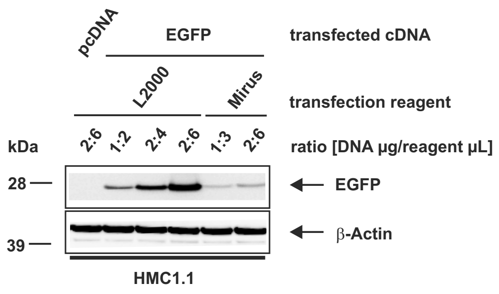


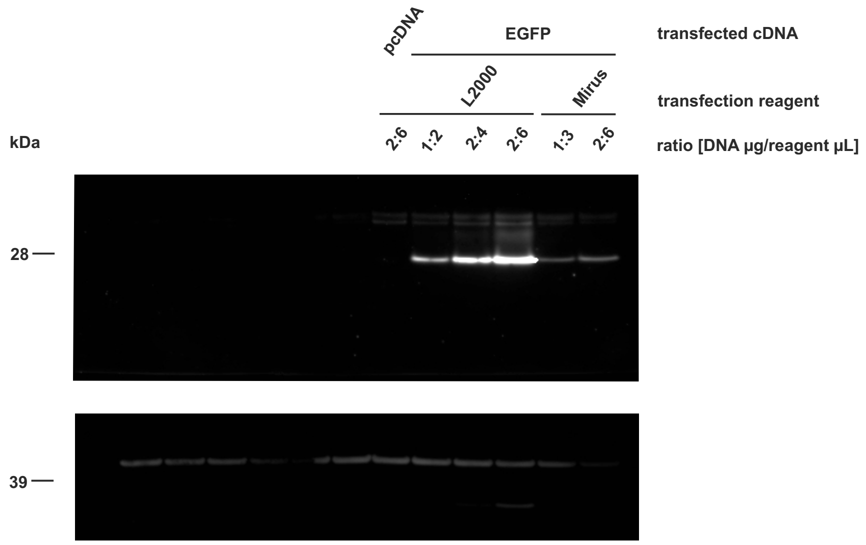

Supplement: Supplementary file 9 — Supplementary Material 9. [file 12964_2025_2048_MOESM9_ESM.pdf]
